# Seasonal and algal diet-driven patterns of the digestive microbiota of the European abalone *Haliotis tuberculata*, a generalist marine herbivore

**DOI:** 10.1186/s40168-018-0430-7

**Published:** 2018-03-27

**Authors:** Angélique Gobet, Laëtitia Mest, Morgan Perennou, Simon M Dittami, Claire Caralp, Céline Coulombet, Sylvain Huchette, Sabine Roussel, Gurvan Michel, Catherine Leblanc

**Affiliations:** 1 Sorbonne Universités, UPMC Université Paris 06, CNRS, UMR 8227, Integrative Biology of Marine Models, Station Biologique de Roscoff, CS 90074, F-29688 Roscoff Cedex, France; 2 Sorbonne Universités, UPMC Université Paris 06, CNRS, FR2424, Genomer, Station Biologique de Roscoff, CS 90074, F-29688 Roscoff Cedex, France; 3 LEMAR, UMR 6539, IUEM, Plouzané, France; 4 France Haliotis, Plouguerneau, France

**Keywords:** Microbe-host interactions, Digestive microbiota, Abalone, Macroalgae, Holobiont

## Abstract

**Background:**

Holobionts have a digestive microbiota with catabolic abilities allowing the degradation of complex dietary compounds for the host. In terrestrial herbivores, the digestive microbiota is known to degrade complex polysaccharides from land plants while in marine herbivores, the digestive microbiota is poorly characterized. Most of the latter are generalists and consume red, green, and brown macroalgae, three distinct lineages characterized by a specific composition in complex polysaccharides, which represent half of their biomass. Subsequently, each macroalga features a specific epiphytic microbiota, and the digestive microbiota of marine herbivores is expected to vary with a monospecific algal diet. We investigated the effect of four monospecific diets (*Palmaria palmata*, *Ulva lactuca*, *Saccharina latissima*, *Laminaria digitata*) on the composition and specificity of the digestive microbiota of a generalist marine herbivore, the abalone, farmed in a temperate coastal area over a year. The microbiota from the abalone digestive gland was sampled every 2 months and explored using metabarcoding.

**Results:**

Diversity and multivariate analyses showed that patterns of the microbiota were significantly linked to seasonal variations of contextual parameters but not directly to a specific algal diet. Three core genera: *Psychrilyobacter*, *Mycoplasma*, and *Vibrio* constantly dominated the microbiota in the abalone digestive gland. Additionally, a less abundant and diet-specific core microbiota featured genera representing aerobic primary degraders of algal polysaccharides.

**Conclusions:**

This study highlights the establishment of a persistent core microbiota in the digestive gland of the abalone since its juvenile state and the presence of a less abundant and diet-specific core community. While composed of different microbial taxa compared to terrestrial herbivores, the digestive gland constitutes a particular niche in the abalone holobiont, where bacteria (i) may cooperate to degrade algal polysaccharides to products assimilable by the host or (ii) may have acquired these functions through gene transfer from the aerobic algal microbiota.

**Electronic supplementary material:**

The online version of this article (10.1186/s40168-018-0430-7) contains supplementary material, which is available to authorized users.

## Background

A holobiont is defined as a meta-organism consisting of a host (e.g., animal, plant, alga) and its associated microbiota. A healthy holobiont is considered to have a resilient microbiota, which may adapt and respond differently depending on external disturbances [1]. The importance of the holobiont concept is increasingly coming into the focus of ecological and clinical research due to the major role of, for instance, digestive microbiota in host digestion, host health, and host development [2–5]. For instance, the ruminant gastro-intestinal tract contains specific bacterial groups able to degrade complex plant cell-wall polysaccharides such as cellulose, hemicelluloses, and pectins, allowing polymer assimilation during digestion and accounting for 70% of the host energy intake [6].

In the marine environment, herbivores feed on macroalgae which live associated with an epiphytic microbiota. These play major roles in health and physiology of macroalgae [7], such as host adaptation to varying environmental conditions [8], bacteria-induced morphogenesis [9, 10], or via their antifouling activity [11]. Reciprocally, algal biomass constitutes a large choice of carbon sources for heterotrophic bacteria, especially polysaccharides, which represent about 50% of the algal dry weight. Red, green, and brown algae each have a different polysaccharide composition and most of the macroalgal polysaccharides are absent from land plants [12, 13]. To feed on seaweeds, marine heterotrophic bacteria have evolved specific enzymes such as agarases, carrageenases, fucoidanases, or ulvan lyases [14, 15]. Therefore, the macroalgal microbiota is significantly enriched in algal polysaccharide-degrading bacteria (mainly *Bacteroidetes* and *Gammaproteobacteria*) in comparison to the water column [16]. Notably, the macroalga-associated bacterial community differs between red, green, and brown algae [17–20].

Most marine herbivores are generalists, meaning they are able to feed on the three macroalgal lineages, but only few studies have been performed on digestive microbiota in marine herbivores. Bacteria have been shown to help degrade macroalgal polysaccharides in the sea hare *Aplysia* spp., the snail *Tegula funebralis*, the limpet *Patella pellucida*, and the iguana *Amblyrhynchus cristatus* [21–24], but these studies considered neither different diet types nor seasonality. The abalone, a gastropod of high economic interest especially in China, Korea, South Africa, and Chile [25, 26], is also a generalist herbivore [27], but its digestive enzymes are not sufficient for macroalgal polysaccharide degradation [28]. This makes the abalone an interesting model to investigate the composition, seasonality, and role of the digestive microbiota associated with several diet types.

We selected four local monospecific macroalgal diets based on abalone food preferences, nutritional quality, and on their biochemical composition: the brown algae *Saccharina latissima* and *Laminaria digitata*, the red alga *Palmaria palmata*, and the green alga *Ulva lactuca*. We determined the composition of the microbiota from the abalone digestive gland, and we tested the following hypotheses: (i) the marine herbivore gastrointestinal tract offers a specific niche for specific marine bacteria, (ii) algal diet, host characteristics, and time influence microbiota composition and fluctuations, (iii) specific microbial groups are associated with the digestion of a monospecific macroalgal diet, allowing for the abalone’ polyphagy.

## Results

Here, we investigate the impact of four monospecific algal diets on the abalone digestive microbial community. For each of the four algal diets, abalone reared in 3 independent cages were sampled at 6 time points over a year and the abalone digestive microbiota from the resulting 72 samples was further studied through a metabarcoding analysis.

### Diet-specific seasonal diversity patterns of the digestive microbial community

Operational taxonomic unit (OTU, defined at 97% sequence identity) richness and evenness of the digestive microbiota were investigated for each algal treatment and sampling date. When all sampling dates were pooled, alpha-diversity did not significantly vary between algal diets on average, but when algal diets were considered separately, alpha-diversity followed different seasonal patterns. In the case of the *L. digitata* diet, samples from colder months (February–April 2012, January 2013) presented significantly lower alpha-diversity indices on average than those from warmer months (Wilcoxon test, *P* < 0.001, Additional file 1: Figure S1, Additional file 2: Table S1). In the case of the *S. latissima* and *U. lactuca* diets, diversity increased over the year and diversity seemed stable with time for the *P. palmata* treatment.

**Fig. 1:**
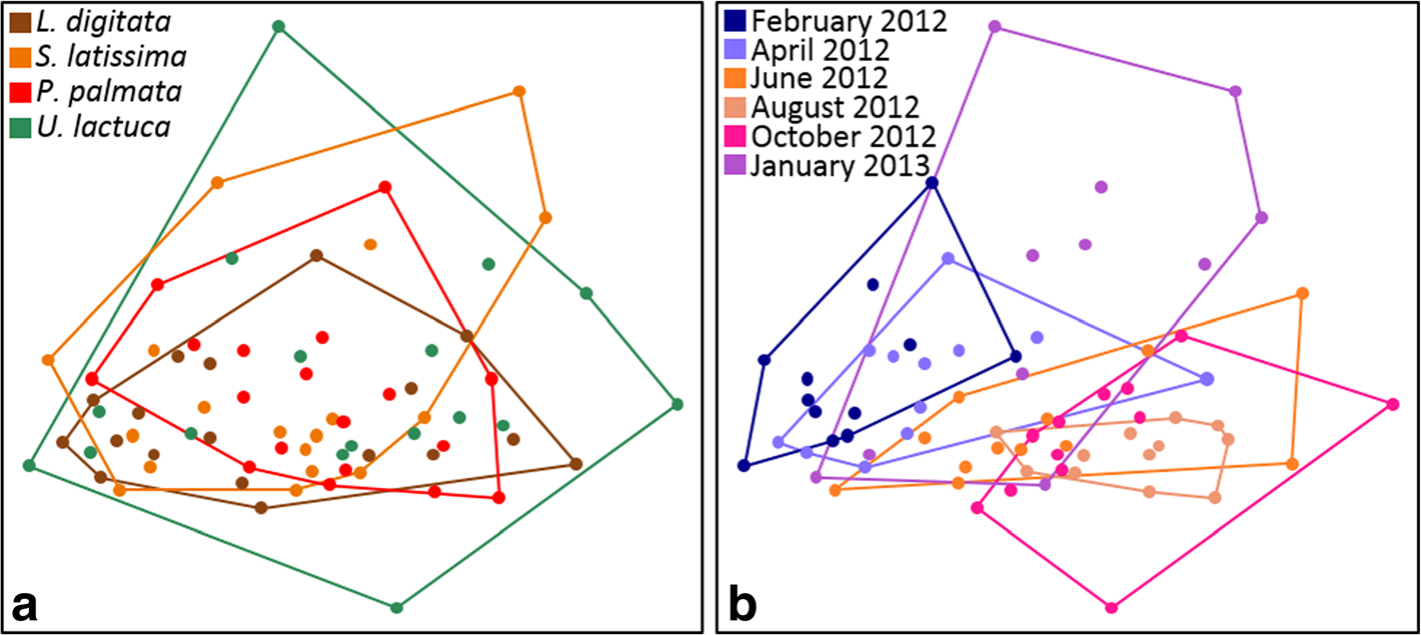
Community structure of the digestive microbiota of the abalone over 1 year. Samples were grouped a posteriori according to one of the four monospecific algal diets (**a**) or a sampling date (**b**). The Bray-Curtis dissimilarity index was calculated after Hellinger transformation of the metabarcoding data set at the OTU level. The low stress value of 18% validates the goodness-of-fit of the two-dimensional representation compared with the original matrix. Significance between groupings of samples according to algal diet, sampling date, or season was tested using ANOSIM (Additional file 3: Table S2)

Similarly, when the structure of the bacterial community associated with the abalone digestive gland was investigated at the OTU level (Fig. 1), diet had only a minor impact on the bacterial community (ANOSIM, *R* = 0.05, *P* < 0.01, Additional file 3: Table S2). However, there was a clear grouping of samples according to season and date (*R* = 0.36 and *R* = 0.43, respectively, *P* < 0.01). When considering each monospecific diet individually, bacterial communities associated with *P. palmata* digestion were more different between sampling dates than between the cage triplicates (ANOSIM: *R* = 0.41–1). Bacterial communities associated with the *U. lactuca* diet showed noticeable differences between the beginning of the experiment (February–April 2012) and January 2013 (*R* = 0.83). Seasonal effects were stronger for the *L. digitata* diet than for the other diets, with samples from colder months separated from those from warmer months. For the microbial communities associated with the *S. latissima* diet, bacterial communities sampled in January 2013 were significantly different from other dates (*R* > 0.8, *P* < 0.001). Overall, diversity patterns of the abalone digestive microbiota showed seasonal patterns specific to a given monospecific diet.

### Fluctuations of OTUs associated with each monospecific algal diet

The distribution of OTUs linked to a monospecific diet was investigated by measuring proportions of OTUs shared between two consecutive sampling dates. For instance, in the digestive microbiota associated with the *P. palmata* diet, there were 29–40% OTUs common to abalone sampled in February 2012 and those sampled in April 2012, meaning that there were about 60–71% new OTUs after 2 months of monospecific diet (Additional file 1: Figure S2A). Over the year, the proportions of OTUs common to two consecutive sampling dates varied between 16–59%, 18–70%, 23–61%, and 10–71% for the *P. palmata, U. lactuca, L. digitata*, and *S. latissima* diets, respectively. These common OTUs, however, accounted for 40–99% of the sequence reads (Additional file 1: Figure S2). About 3% OTUs were present the whole year for each monospecific diet and corresponded to a large proportion of sequence reads (from 88–97% in each cage). Altogether, this indicates that the rarest OTUs fluctuate with time while the most abundant OTUs remain in the digestive gland during the year, indicating the presence of a dominant core microbial community associated with each algal diet.

### Few bacterial genera dominate the core digestive microbiota at the start of the experiment

**Fig. 2:**
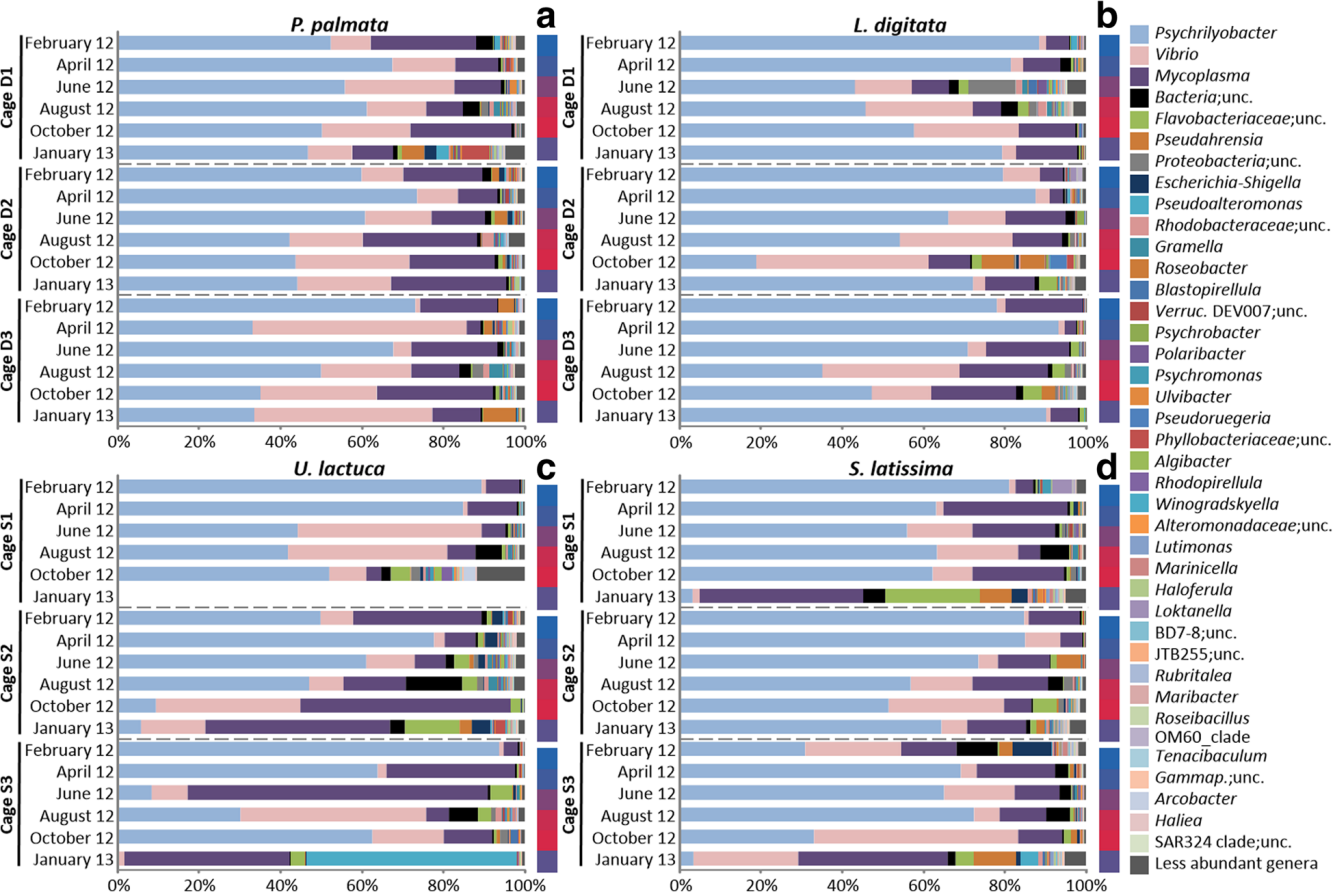
Relative abundance and taxonomical composition of abalone digestive microbiota at the genus level. Each panel represents the digestive bacterial community associated with feeding with *Palmaria palmata* (**a**), *Laminaria digitata* (**b**), *Ulva lactuca* (**c**), and *Saccharina latissima* (**d**). The taxonomical composition is represented for each cage triplicate over 1 year. Water temperature is indicated on the right side of each panel (values varying from 10 °C (blue) to 15 °C (red)). Less abundant genera: total number of sequence reads of these 651 genera for clearer representation in the figure. In **c**, the sample from cage U1 in January 2013 is missing. For unclassified genera, genera were assigned to the lowest taxonomic level identified. *Verruc.*, *Verrucomicrobiales*; BD7-8, BD7-8 marine group; JTB255, JTB255 marine benthic group; OM60_clade, *Alteromonadaceae* OM60_clade; *Gammap.*, *Gammaproteobacteria*; unc, unclassified

In February 2012, after abalone had not been fed for a week, the most abundant phyla were *Fusobacteria*, *Tenericutes*, and *Gammaproteobacteria* (average of 72, 14, and 9% of all sequence reads per cage, respectively, Additional file 1: Figure S3). The phylum *Fusobacteria* was mostly composed of the genus *Psychrilyobacter* (99.9% of sequences in the phylum; 31% to 94% of all sequence reads per cage) and the phylum *Tenericutes* of *Mycoplasma* (99.9% of sequences in the phylum; 3–32% of all sequence reads per cage). The third most abundant taxonomic group, the class *Gammaproteobacteria*, was composed mainly of *Vibrio* (68% of sequence reads in the phylum; 1–23% of all sequence reads per sample). Other genera present in all cages represented 2% or less of the sequence reads per cage (Fig. 2). These global patterns validate the dominance of few genera in the abalone digestive microbiota at the starting point of the experiment.

### Composition and seasonal and diet-driven fluctuations of the core digestive microbiota

**Fig. 3:**
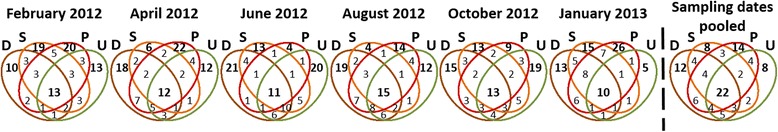
Comparison of the microbial OTU composition associated with the four algal diets. Comparisons are described by proportions of shared or unique OTUs and were made for each sampling date from February 2012 to January 2013 and for the six sampling dates pooled according to diet. D, *L. digitata*; S, *S. latissima*; P, *P. palmata*; U, *U. lactuca*

**Fig. 4:**
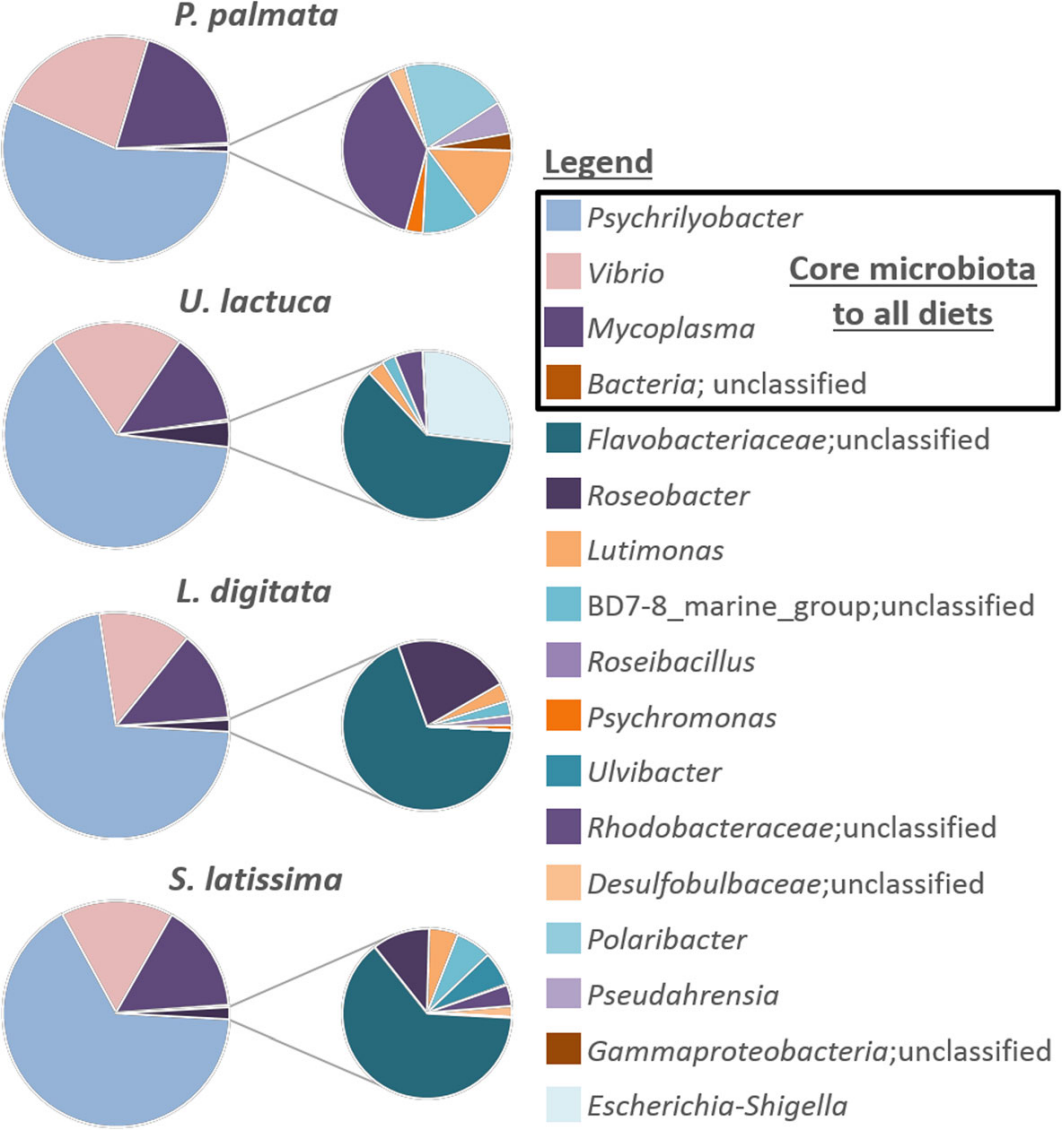
Composition of the digestive core microbiota at the genus level associated with a monospecific diet. Pie charts on the left represent proportions of sequence read abundances, at the genus level, representative of the core microbiota of a monospecific diet. Pie charts on the right represent detailed proportions of less abundant genera representative of the core microbiota of a monospecific diet

Over a year, 10–15% OTUs were shared by the 4 monospecific diets. For all sampling dates pooled per algal diet, 22% of the OTUs of the whole community corresponded to a core community shared by the 4 diets (Fig. 3). To better understand the potential functioning of this core community, we focused on temporal fluctuations of these OTUs constantly found in the digestive microbiota. We considered as the core microbiota only the OTUs occurring in all samples, considering each of the four algal diets, for each cage triplicate and during the whole year. Six OTUs belonging to the classes *Fusobacteriia*, *Gammaproteobacteria*, and *Mollicutes* fit this definition: one *Psychrilyobacter* OTU, two *Vibrio* OTUs, two *Mycoplasma* OTUs, and an unclassified OTU. In terms of sequence read abundance, these six OTUs represented about 86% of the microbiota (Fig. 4), although relative contribution of each of these OTUs was variable (Fig. 2). Apart from these 3 dominating core genera, 5 to 8 additional genera belonged to a core microbiota specific to each algal diet and they represented lower relative abundances of OTUs, ranging from 0.001 to 24.5% of sequence reads per cage (Fig. 4). Some OTUs belonged to genera found with at least two monospecific diets: *Lutimonas*, *Psychromonas*, *Roseobacter*, and four unclassified genera. Other genera were specific to a given diet. *Polaribacter* and *Pseudahrensia* were associated with the *P. palmata* diet, *Escherichia*-*Shigella* to the *U. lactuca* diet, *Roseibacillus* to the *L. digitata* diet, and *Ulvibacter* was associated with the *S. latissima* diet (Fig. 4).

### Algal polysaccharide digestion potential of the core digestive microbiota

To further explore the potential role of *Psychrilyobacter*, *Vibrio*, and *Mycoplasma* in algal digestion, we compared their carbohydrate-active enzymes (CAZymes) and sulfatase contents to that of *Zobellia galactanivorans* Dsij^T^, a model bacterium for the bioconversion of algal polysaccharides [29]. This was done based on the public CAZy and SulfAtlas databases (Table 1, Additional file 4: Table S3 and Additional file 5: Table S4). This approach is of course limited by the availability of genomes of some genera in public databases. Still, similar results were found in several available genomes of a given genus and are likely to reflect at least some functions present in abalone digestive microbiota. As there was no *Psychrilyobacter* genome available in the CAZy and SulfAtlas databases, we manually annotated the CAZyme- and sulfatase-coding sequences in the only genome of the genus available to date in NCBI Genome [30], i.e., that of *Psychrilyobacter atlanticus* DSM 19335, a strictly anaerobic bacterium isolated from marine sediment [31]. Compared to the genome of *Z. galactanivorans* Dsij^T^, the genome of *P. atlanticus* DSM 19335, contains far fewer GHs and sulfatases as well as no polysaccharide lyases (Table 1, Additional file 5: Table S4). These genes are likely to enable the degradation of extracellular oligosaccharides, but not the degradation of complex algal polysaccharides (Additional file 5: Table S4). To evaluate the ability of *Vibrio* to degrade algal polysaccharides in the digestive gland, we examined the CAZyme and sulfatase content in the 127 *Vibrio* genomes available in the CAZy and SulfAtlas databases to date (Additional file 4: Table S3 and Additional file 5: Table S4). The 127 genomes present lower numbers of CAZyme and sulfatase families than the *Z. galactanivorans* genome (Table 1, Additional file 4: Table S3 and Additional file 5: Table S4). However, all examined *Vibrio* genomes contain at least one enzyme involved in chitin and/or cellulose metabolism (GH9, GH18, GH19, GH20, GH94 families) and at least one enzyme degrading extracellular oligosaccharides. Some *Vibrio* genomes such as *V. crassostreae* 9CS106, *V. breoganii* FF50, *V. owensii* XSBZ03, *V. halioticoli* NBRC 102217, three strains of *V. alginolyticus* (ATCC 33787, K08M4, and ATCC 17749), and *V. harveyi* also are able to degrade the red algal polysaccharide agarose (*aga* genes from families GH16, GH50), laminarin (*lam* genes family GH3), and/or alginate (*alyA* genes from families PL6, PL7, PL15, PL17) from brown algae (Additional file 4: Table S3, [32]). For *Mycoplasma*, we examined 111 genomes, which show poor capacities to degrade complex carbohydrates and no ability to degrade algal polysaccharides (Additional file 5: Table S4, [33]).

**Table 1:** Total number of putative glycoside hydrolase (GH), polysaccharide lyase (PL), and sulfatase genes in selected genomes

	GH^a^	PL^a^	Sulfatases	References
*Psychrilyobacter atlanticus* DSM 19335	13^b^ 23^c^	0 0	0 2	[32]
127 *Vibrio* genomes	9–27 22–70	0–5 0–21	0–5 0–9	[33, 34]
111 *Mycoplasma* genomes	0–6 0–12	0–1 0–1	0 0	[31]
*Z. galactanivorans* Dsij^T^	44 141	8 15	17 71	[27]

This table also contains total number of GH, PL, and sulfatase families in selected genomes from the three genera of the core digestive microbiota of the abalone and of *Zobellia galactanivorans* DsijT, a model bacterium for algal polysaccharide degradation. For more details on the CAZyme and sulfatase content, see Additional file 4: Table S3 and Additional file 5: Table S4

^a^CAZyme class

^b^Total number of CAZyme or sulfatase families in the given class

^c^Total number of CAZyme or sulfatase genes in the given class

*Z. galactanivorans Zobellia galactanivorans*; *GH* glycoside hydrolase, *PL* polysaccharide lyase. Data retrieved from the CAZy and SulfAtlas databases (www.cazy.org, [35], http://abims.sb-roscoff.fr/sulfatlas/, [36])

Among the five to eight additional core genera per diet, we only found genomes of the genera *Roseobacter*, *Psychromonas* (two genomes each), and *Polaribacter* (six genome) in the CAZy and SulfAtlas databases, and we searched for their algal polysaccharide degradation abilities (Fig. 4, Additional file 4: Table S3). *Roseobacter* (a genus only found associated with the two brown algal diets) shows a low capacity to degrade algal polysaccharides with only some secreted exo-glycosidases. *Psychromonas* (associated with the *L. digitata* and *P. palmata* diets) is likely able to degrade brown algal polysaccharides with several enzymes acting on extracellular oligosaccharides and a laminarinase from family GH16, but there was no sign of enzymes acting on red algal polysaccharides. All six genomes of the genus *Polaribacter*, associated with the *P. palmata* diet, contain enzymes acting on red algal polysaccharides, such as agar or porphyran (e.g., enzymes from families GH16, GH86, or GH117), or more specifically on carrageenans (*P.* sp. KT25b contains 2 iota-carrageenases from family GH82). Most of these genomes also encode enzymes able to degrade major algal cell wall polymers such as cellulose and hemicelluloses (e.g., presence of an endo-1,4-β-xylanase from family GH10, a GH39 xylosidase, and enzymes from families GH43, GH51, GH53, GH115, GH127).

### Ecological patterns of the whole and the core digestive microbiota

Potential relationships between the structure of the digestive microbiota and contextual parameters were investigated by applying a multivariate variation partitioning approach. Biological variation of the digestive microbiota was significantly linked to the pure effect of diet composition (adjusted *R*^*2*^ = 5.4%, *P* < 0.05). Sampling dates, the combined effect of abalone and algal composition, the combined effect of algal composition and sampling dates, and the effect of the three explanatory variables together also explained the structuring of the microbial community (adj. *R*^*2*^ = 2.2%, 7.4%, 12.2%, 2.5%, respectively, Additional file 1: Figure S4). Overall, 30.8% of the biological variation of the microbial community structure was explained by abalone characteristics, algal composition, and sampling dates (*P* < 0.001, Additional file 1: Figure S4). The remaining 69.2% of the biological variation not explained by the selected model suggests the effect of additional variables not included in this analysis. Still, our analysis clearly demonstrates a relationship between algal diet composition, sampling dates, and the structure of the digestive microbiota.

**Fig. 5:**
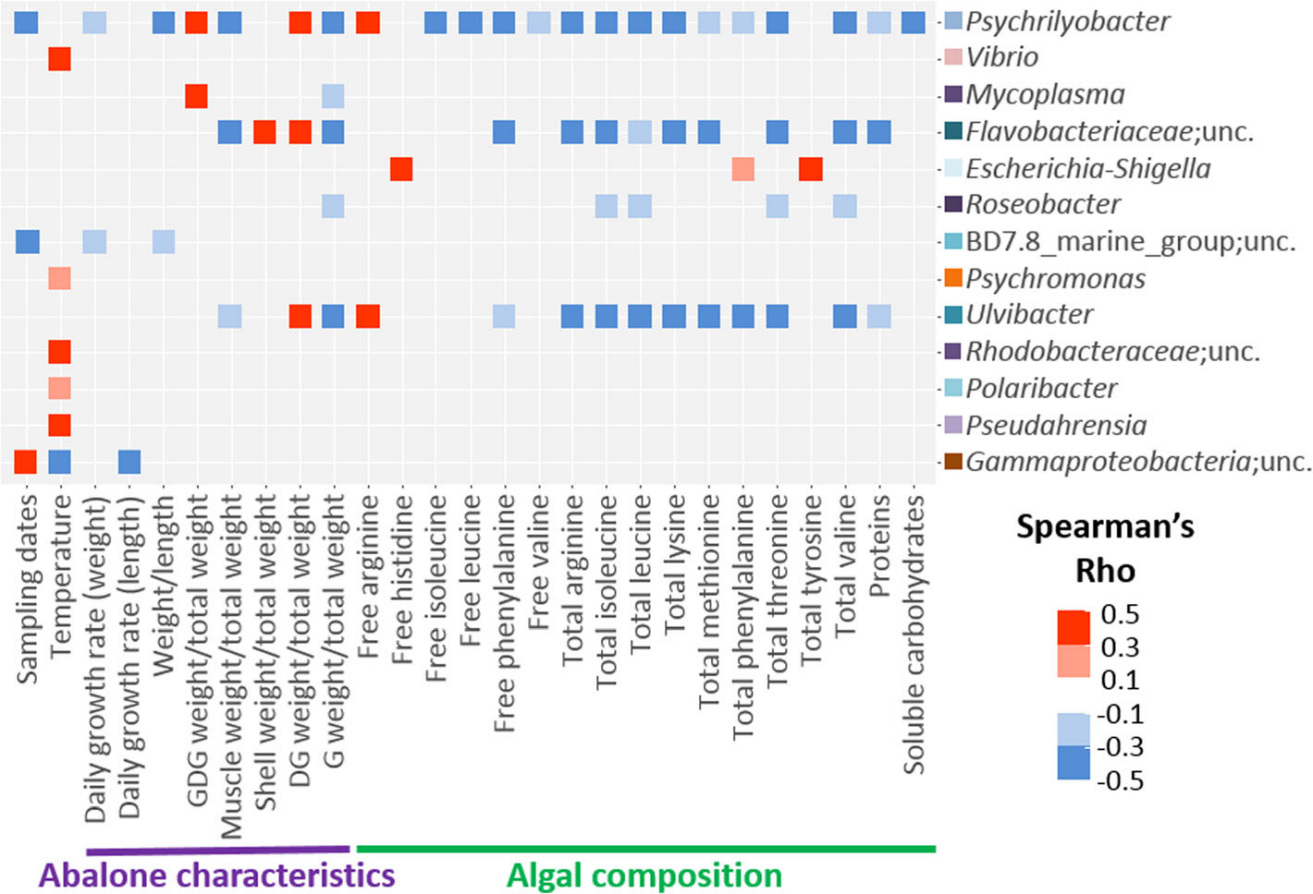
Pairwise comparison between genera of the core microbiota and contextual parameters. Pairwise comparisons were measured by Spearman correlation. The core microbial genera were ordered according to sequence read abundance; with *Psychrilyobacter* being the most abundant group. The strength of the correlation is indicated by a heatmap colored from red to blue. The horizontal axis corresponds to sampling dates, temperature, abalone characteristics and algal diet composition. Only significant correlations after application of the Benjamini-Hochberg correction for multiple testing were represented. GDG, gonado-digestive gland; DG, digestive gland; G, gonad. Unc, unclassified

Pairwise comparisons between individual bacterial genera from the core digestive microbiota (Fig. 4) and contextual parameters showed specific ecological patterns. The genera *Psychrilyobacter*, an unclassified *Flavobacteriaceae*, and *Ulvibacter* followed similar patterns. They increased in relative OTU abundances with a decreasing content in mostly the same algal biochemical compounds and with increasing abalone digestive gland weight. *Mycoplasma* also showed similar patterns related to abalone characteristics. OTU proportions of *Vibrio*, *Psychromonas*, an unclassified *Rhodobacteraceae*, *Polaribacter*, and *Pseudahrensia* likely increased with increasing temperature and no significant relationships were found with other parameters (Fig. 5).

## Discussion

In holobionts, the digestive enzymes of the host do not allow a complete degradation of complex polysaccharides which suggests the importance of a digestive microbiota. Marine generalist herbivores such as the abalone face the challenge of feeding on the three marine macroalgal lineages, which are composed of different combinations of neutral, carboxylated or carboxylic-bearing and sulfated polysaccharides [12]. Here, we determine the taxonomic composition and the seasonal structure of the digestive microbiota of the abalone holobiont, a marine gastropod grown in natural conditions on four monospecific diets over a year. This long-term study revealed that (i) the marine herbivore gastrointestinal tract offers a specific niche for a dominant core microbiota, constitutive of the abalone holobiont, (ii) microbiota composition and fluctuations are correlated to seasonal variations of contextual parameters, and that (iii) few specific microbial groups were associated with a monospecific macroalgal diet.

### The abalone holobiont features a characteristic core microbiota well established in the digestive gland

Three bacterial core genera, the *Fusobacteria Psychrilyobacter*, the *Tenericutes Mycoplasma*, and the *Gammaproteobacteria Vibrio*, dominated the abalone digestive microbiota for the whole year and for each of the monospecific algal diet. Bacteria from these genera are known as obligate (*Psychrilyobacter*) or facultative anaerobes (*Mycoplasma* and *Vibrio*) [31, 34, 35]. This unexpected dominance of a core microbiota through the entire year may not only be due to the initial diet composition but also to gut morphology and its physicochemical and biochemical conditions [2]. The abalone gastrointestinal tract offers stable environmental conditions, which are microaerophilic or anaerobic, with an acidic pH of 5.3–6 in the crop and the digestive gland [36, 37]. It has been previously shown that an acidic pH together with the presence of volatile fatty acid in the rumen is toxic for some bacteria [38] and thus likely to select for bacteria adapted to such conditions. Indeed, anaerobic bacteria from the classes *Fusobacteriia* and *Mollicutes* are only rarely found in coastal waters or associated with macroalgae [39–41]. However, these bacterial groups seem rather typical of digestive organs of marine holobionts as they are found in the sea squirt *Ciona intestinalis*, the limpet *Patella pellucida*, and marine carnivores [21, 42, 43]. Members of *Vibrio* are well known as pathogen of animals, and it is the most common bacterial genus in guts of marine animals such as the intestinal mucosa of marine fishes [35, 44–46]. *Vibrio* is also found associated with algae such as *Ascophyllum nodosum*, *S. latissima*, and decomposing fronds of *Laminaria* spp. [11, 16, 47]. Other gut microbiota also contain dominant core communities, e.g., the human gut microbiota is dominated by anaerobic bacteria belonging to *Firmicutes* and *Bacteroidetes*, representing more than 98% of 16S rRNA sequences, with few other anaerobes from other phyla and only a couple of aerobic *Actinobacteria* [6, 48, 49]. Gut microbiota of terrestrial gastropods are also dominated by a low diversity of anaerobes, including the *Gammaproteobacteria Enterobacter*, some *Firmicutes*, and *Bacteroidetes* [50, 51]. Therefore, the dominant digestive microbiota constitutive of the abalone holobiont comprises bacterial taxa typically found in marine environments and these taxa are not dominant in the gut of terrestrial holobionts.

Additionally, a less abundant core microbiota was specific to at least a monospecific diet and contained aerobic genera: the *Flavobacteriia Lutimonas*, *Ulvibacter*, and *Polaribacter*; the *Alphaproteobacteria Roseobacter* and *Pseudahrensia*; the *Gammaproteobacteria Psychromonas* and *Escherichia*-*Shigella*; and the *Verrucomicrobia Roseibacillus* [52–59]. Notably, all members of this diet-specific and less abundant core microbiota, with the exception of *Pseudahrensia*, were identified during a phytoplankton bloom in temperate coastal waters in the North Sea [41]. This suggests that these bacteria may not only be part of the epiphytic algal diet microbiota but could also be part of the bacterioplankton and be ingested along with the alga. For instance, members of the genera, *Roseobacter*, *Ulvibacter*, and *Roseibacillus*, were previously isolated from green and brown algae, and *Roseobacter* was found associated with the red alga *Gracilaria vermiculophylla* [19, 53, 55, 58]. Strains of *Polaribacter* and *Psychromonas* were previously found associated with *Laminaria* sp. and *S. latissima*, and both genera have members that can degrade red algal polysaccharides [54, 57, 60, 61]. *Lutimonas* and *Pseudahrensia*, however, were found only in marine invertebrates or seawater but not associated with algae [52, 56, 62–64]. The occurrence and maintenance over time of few diet- or plankton-derived specific bacteria suggests a selectivity of some genera, which could participate to the global functioning of the digestive gland in abalone. The remaining OTUs that are not part of the core microbiota are transient bacteria and represent a smaller fraction of the digestive microbiota over the year (up to 14% of sequence reads per cage). These rarer bacteria are probably ingested along with the algal diet and have either (i) died and left DNA remains in the digestive gland or (ii) survived the selection pressure and may participate in the algal recycling in the abalone digestive gland [65].

Such organization of the microbiota in abalone digestive gland consists of two groups of bacteria: one resident group comprising the dominant bacteria and another one comprising the less abundant diet-specific bacteria and rarer bacteria which are transient over the year. Dominance of few bacterial taxa in the digestive microbiota may contribute to holobiont health and, as proposed in the case of human gut microbiota, such stability may allow for a certain resiliency in response to invasive pathogens or other exogenous microbes [1, 3]. Further, the presence of less abundant taxa may also participate in abalone holobiont health. Previous studies on human lung- or plant-associated microbes showed that rare species were also involved in host protection against pathogens [66, 67]. On another hand, in a case of a disturbance strong enough to destabilize the core microbiota, these rarer OTUs may become more abundant and fill the niche previously occupied by the dominant bacterial community. They can either fill the same functions as the core microbiota and contribute to holobiont health or become pathogens [3, 68].

### Seasonal variations of abalone digestive microbiota

Our experiment was carried out in an abalone farm located at the French Brittany coast where waters are impacted by seasonal variations specific to this permanently mixed ecosystem [69, 70]. These temperate coastal waters were coldest in February–April 2012 and January 2013 (10–11.5 °C) and warmest from June to October 2012 (12.5–15 °C, Additional file 6: Table S5). Several studies in temperate coastal areas showed seasonal succession and structuring of the bacterial community [39, 41, 71]. Accordingly, even if three genera were dominating for the entire year, the community structure of abalone digestive microbiota was impacted by seasonal variability (Fig. 1), and 17.6% of the biological variation of the microbiota was explained by the effect of sampling dates alone and by its combined effect with abalone characteristics and algal biochemical content. We found one study on seasonality of intestinal microbiota in a marine organism, the farmed Atlantic salmon, which showed highest number of bacteria in the gut with the highest temperature in August but a community composition that was quite stable over the year [72]. More temporal studies were done in fresh water, where several examples of seasonality of fishes’ digestive microbiota could be observed either in terms of cell number or taxonomic composition [73]. In our study, however, the biochemistry of the four algae showed significant seasonal variations through the year, as tested with their content in soluble carbohydrates, dry algal matter and protein (Friedman test with post hoc test after Nemenyi, Roussel, personal communication), which had a significant effect on the digestive microbiota, explaining 5.4% of the biological variation of the microbiota (Additional file 1: Figure S4). Also, individual microbial genera of the dominant core and the less abundant microbiota were differently correlated with temporal fluctuations of contextual parameters (Fig. 5). For instance, the presence of *Vibrio*, *Psychromonas*, an unclassified *Rhodobacteraceae*, *Polaribacter*, and *Pseudahrensia* was positively correlated with increasing water temperature (Fig. 5). Seasonal variations have been previously described for *Vibrio* and *Polaribacter* in the plankton and in particle-attached fractions in coastal waters [41, 74], but no study could be found on seasonality in coastal waters of *Psychromonas* and of *Pseudahrensia*. Seasonal fluctuations of *Vibrio* and the less abundant and diet-specific core microbiota may thus be not only influenced by algal diet biochemical content but also by the seasonality of the water column composition.

### Establishment and potential functioning of abalone digestive microbiota

A major factor that may explain part of the unexplained 69.2% of biological variation is microbial interactions. Following niche construction theory, microbiota play a role in modifying physicochemical conditions of the digestive gland and apply selection pressure to themselves, their descendants, and immigrating cells [75]. Here, the selection of the holobiont microbiota may result in the cooperation between degraders of algal polysaccharides and fermenters of the resulting products in microaerophilic and acidic conditions. Indeed, aerobic bacteria ingested with the diet have been shown to rapidly consume all the available oxygen in the gastrointestinal tract, providing ideal conditions for fermentation of pyruvate from algal polysaccharide degradation [36, 76]. Genomes closely related to *Psychrilyobacter*, *Mycoplasma*, and *Vibrio* possess enzymes for the acetate pathway, allowing pyruvate fermentation to short chain fatty acids (SCFA) for further assimilation by the host (Additional file 5: Table S4, Additional file 8, Fig. 6). As hypothesized for gut colonization of the sea squirt *Ciona intestinalis* [42], these dominant fermenters, may be part of a resident anaerobic biofilm within the outer mucus layer at the epithelial cell surface and benefit from the host digestive properties such as the secretion of mucin-like glycoproteins [77].

**Fig. 6:**
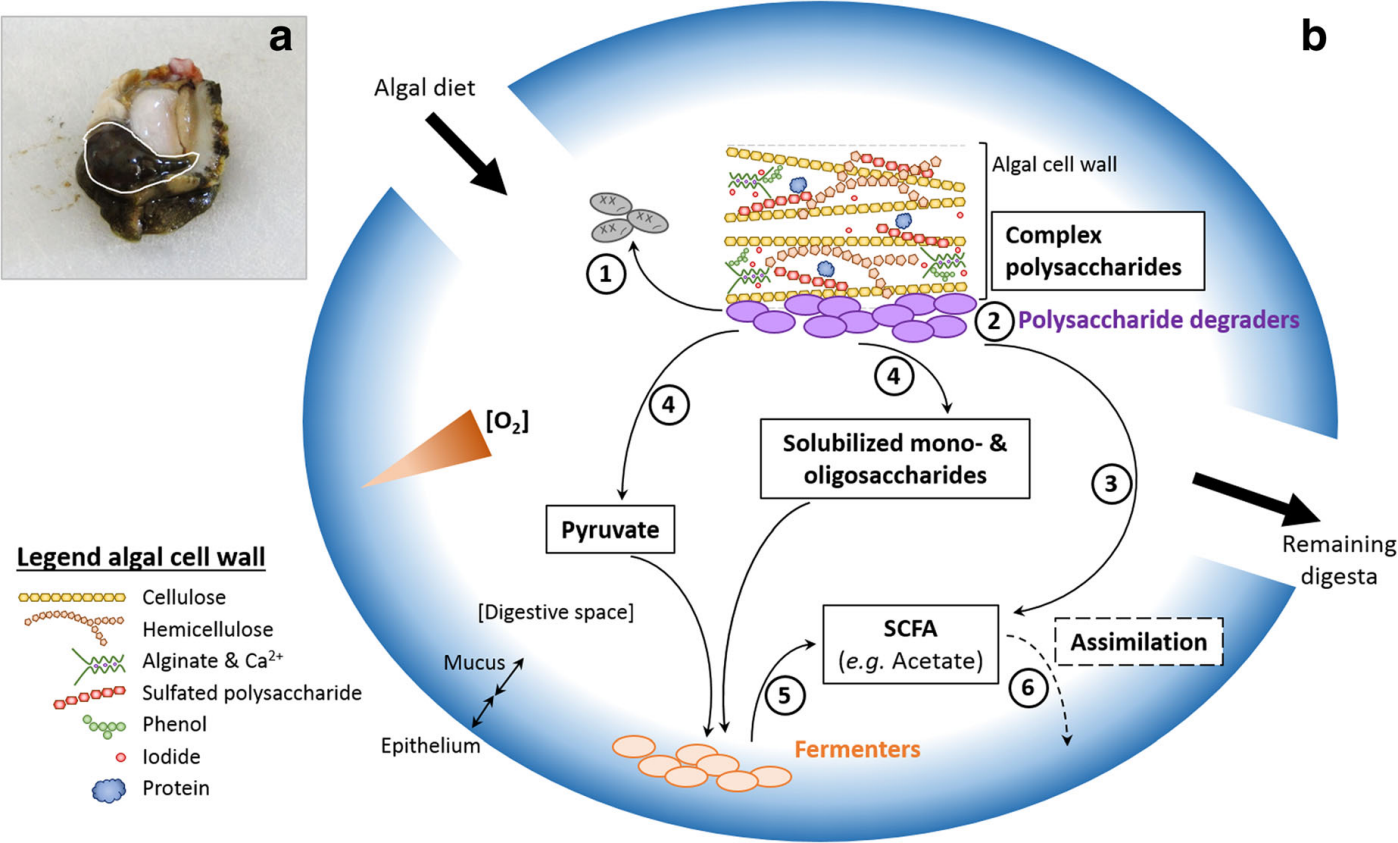
Proposed processes of algal polysaccharide degradation in the abalone digestive gland. For information, localization of the abalone digestive gland on an abalone without the shell (**a**). The schematic illustration recapitulates hypothetical processes of algal polysaccharide degradation in the case of a brown algal cell wall (**b**). The algal diet comes along with epiphytic bacteria, including (*1*) strictly aerobic bacteria which may die due to specific physiochemical conditions (low O_2_, low pH) and (*2*) facultative aerobic bacteria, which may act as primary degraders of complex algal polysaccharides. Primary degraders may (*3*) directly ferment polysaccharides to short-chain fatty acids (SCFA, e.g. *Vibrio*) or (*4*) transform polysaccharides into pyruvate or solubilized mono- and oligosaccharides (members of *Flavobacteriia*, *Alpha*-, and *Gammaproteobacteria*). These may then be (*5*) fermented by strictly or facultative anaerobic bacteria (e.g. *Psychrilyobacter*, *Mycoplasma*), which are probably localized in an anaerobic or microaerophilic part of the gland, such as the epithelial mucus. Resulting SCFA are then (*6*) assimilated by the host. Parts of the illustration are inspired from [6, 96]. Abalone picture: courtesy of Monique Ras

Regarding algal polysaccharide degradation in abalone digestive gland, two scenarios are possible, involving either (i) the core dominant community only, or (ii) the core dominant community together with the less abundant and the transient community (Fig. 6). The first scenario is based on putative lateral acquisitions of CAZymes by the three dominant genera of the abalone digestive microbiota, e.g., from algal epiphytic bacteria. Earlier studies have shown the possibility of horizontal transfer of CAZyme-genes (e.g., beta-porphyranases, GH117, alginate lyases) from algal epiphytic bacteria to intestinal bacteria from human or marine fishes in relation to the consumption of seaweeds [78–80]. In this scenario, the less abundant core microbiota and transient bacteria would play only a complementary role in algal degradation. The second scenario assumes that the dominant bacteria in the microbiota are largely incapable of degrading complex algal polysaccharides, as suggested by their low CAZyme and sulfatase content found in our survey. Only *Vibrio* may possess a limited capacity to degrade algal polysaccharides as suggested by the analysis of *V. crassostreae* and *V. breoganii* genome, which are often found in the gut of invertebrates, and genomes of the species *V. crassostreae*, *V. harveyi*, and *V. halioticoli* which were previously isolated from abalone gut and described as alginate degraders [44, 81]. Also, *V. halioticoli* isolated from abalone are able to produce SCFA such as acetate from alginate (Additional file 5: Table S4 and Additional file 8, [16, 81, 82]). The less abundant and diet-specific core microbiota may complement the capacities of *Vibrio* as they also have the potential to degrade algal polysaccharides, e.g., *Polaribacter*, *Roseobacter*, or *Psychromonas* [54, 55, 60, 81].

In either of the two scenarios, the abalone holobiont seems to provide the ideal conditions in the digestive gland to maintain a core microbiota which probably plays a major role in producing assimilable products for the abalone from complex dietary compounds such as algal polysaccharides.

## Conclusion

The most striking finding here is that the abalone digestive microbiota seems to be already established during the first year of life of the abalone and that seasonal variation, feed type, and quality only account for a small proportion of the variability of the microbiota. This is in agreement with the existence of a relatively stable digestive microbiota, inherent part of the holobiont. In the case of a healthy abalone holobiont, digestive gland conditions may control the composition of the microbiota, and in return, this microbiota may be adapted to seasonal variations. In addition, transient and rare algal epiphytic bacteria may remain in the niche, depending on their ability to assist in algal polysaccharide degradation. In the abalone holobiont, two non-exclusive scenarios may occur during digestion: (i) the dominant fermenters have acquired polysaccharide degrading genes by lateral gene transfer from aerobic epiphytic algal microbiota, and degrade and ferment algal polysaccharides themselves, (ii) the aerobic epiphytic algal microbiota cooperate with the dominant fermenters to transform algal polysaccharides to SCFA. To test these hypotheses and to elucidate further details on the functional plasticity of the dominant fermenters or the aerobic microbiota, we would need to investigate microbial genes associated with abalone digestion, using metagenomic tools or trying to isolate candidates and further analyze their genomes.

Altogether, these results on a marine generalist herbivore further highlight the specificity and durability of the holobiont association, between the host and its digestive microbiota, even in an open and mixing natural environment such as seawater. A better understanding of the functioning of this marine holobiont will contribute to increase our knowledge on abalone biology and to improve natural and sustainable cultivation methods.

## Methods

### Study site and sampling procedure

European abalone (*Haliotis tuberculata* Linnaeus, 1758), marine herbivorous gastropods, were bred at the sea-based abalone farm France Haliotis, on the coast of Brittany, France (48°36′46 N; 4°33′30 W). In summer 2010, juveniles were bred on land in nursery tanks for a year before being transferred to sea-based experimental cages and fed with a mixed algal diet composed of *P. palmata*, *S. latissima*, and *L. digitata* for about 6 months. Abalone were then not fed for a week before the beginning of the experiment (Additional file 1: Figure S5). The experiment started in February 2012, and three abalone were randomly sampled from each cage. Abalone were then fed ad libitum every month with one of the monospecific diets: *P. palmata*, *U. lactuca*, *L. digitata*, *S. latissima*, and this was replicated in three cages for each algal treatment. About every 2 months from February 2012 to January 2013, 3 abalone were taken randomly from each of the 12 cages and the resulting 216 abalone were frozen at − 80 °C until further analysis of the digestive bacterial microbiota (Additional file 1: Figure S6). On the same dates, additional abalone were sampled for measurements of growth and sexual maturation and the fresh algal diet was sampled before abalone feeding to subsequently analyze algal biochemical composition (see Additional file 8 for details).

### DNA extraction, metabarcoding, and sequence processing

As it is closely surrounded by the gonads, the digestive gland could not be separated from the gonads during dissection. The gonado-digestive gland (simplified as the digestive gland throughout the manuscript) of 216 abalone was thus dissected as a whole and was freeze-dried before tissue grinding (Additional file 1: Figure S7). DNA was extracted from ground lyophilized glands and the V3-V4 variable region of the 16S rRNA gene was amplified using primers targeting *Bacteria*. Amplicons from three abalone of the same conditions (same cage and same sampling date) were then pooled before MiSeq paired-end sequencing (Illumina, San Diego, CA, USA), which resulted in about 10 million sequence reads. Sequence quality check, curation, alignment, and chimera removal were done using publicly available scripts (fastx_toolkit, http://hannonlab.cshl.edu/fastx_toolkit/; get_paired.py, http://abims.sb-roscoff.fr, mothur v.1.34.4, [83]). Sequences were taxonomically classified with the RDP classifier [84] on the Silva release 119 [85] and OTUs were clustered at 97% sequence identity using the average neighbor algorithm (see Additional file 8 for details, [86]).

### Diversity analyses and ecological patterns of the digestive microbiota

Shannon and Simpson indices were calculated from 1000 resamplings of the original table to the lowest number of sequence reads per sample (12,997 sequence reads). Dissimilarities in community structure were studied for all taxonomic levels using the Bray-Curtis dissimilarity index after Hellinger transformation of the data set [87]. The analysis of similarity (ANOSIM) allowed testing the grouping of samples according to sampling date, season, or algal diet.

Contextual parameters were measured in a parallel study, and the best combinations of these to explain the variation in the microbial community matrix were determined by forward selection (Additional file 8 and Roussel, personal communication). Parameters were log_10_-transformed, and the community matrix was standardized by Hellinger transformation [87]. Variation partitioning was then used on the community matrix to investigate the effects of the selected parameters (abalone characteristics, algal composition, sampling date) and of their covariation on the structure of the microbiota [88, 89].

Data analyses were performed using PAST version 2.17 [90] and the R version 3.2.2 programming environment as well as the vegan package and custom R scripts ([71], http://www.mpi-bremen.de/en/Software_4.html).

### Gene annotation of the core genera

Carbohydrate active enzymes (CAZymes) and sulfatases contents from 127 genomes of the genus *Vibrio*, 111 genomes of *Mycoplasma*, 6 genomes of *Polaribacter*, 2 genomes of *Roseobacter*, and 2 genomes of *Psychromonas* were retrieved from the CAZY and SulfAtlas websites [32, 91–93]. No *Psychrilyobacter* genome was available on the CAZY website, so the *Psychrilyobacter atlanticus* DSM 19335 genome was downloaded from NCBI, project accession PRJNA195804 [94] as well as genomes from the fish-associated mycoplasma *Mycoplasma mobile* 163K [33] as an example of marine *Mycoplasma* and *Vibrio halioticoli*, project accession BAUJ00000000 [95], a *Vibrio* isolated from abalone [94]. CAZyme and sulfatase annotations of each sequence were performed manually by homology with sequences from the CAZy and SulfAtlas databases, from which fasta files can be downloaded, using a minimum sequence identity of 25%, and a minimum query and hit coverage of 70% between amino-acid sequences. To investigate pyruvate to acetate fermentation, a database including sequences of the enzymes involved in the according pathways was manually created to further annotate genes from *P. atlanticus*, *M. mobile*, and *V. halioticoli* by homology, following the same conditions as for CAZyme gene annotation (for details on the pyruvate to acetate fermentation database, see Additional file 6: Table S5, Additional file 7: Table S6 and Additional file 8). Gene annotations were carried out using the ngKLAST software (ngKLAST release 4.2, 2007–2013, Korilog SARL, France).

## Additional files

Additional file 1: **Figure S1.** Alpha-diversity of the digestive microbiota of abalone, as described by the Shannon (left panels) and Simpson (right panels) indices. **Figure S2.** Fluctuations of the digestive microbiota between consecutive dates over 1 year. **Figure S3.** Relative abundance and taxonomical composition of the digestive microbiota at the phylum level for abalone fed on *Palmaria palmata* (A), *Laminaria digitata* (B), *Ulva lactuca* (C), and *Saccharina latissima* (D). **Figure S4.** Ecological patterns of the digestive microbiota explained by a model of contextual parameters. **Figure S5.** Experimental site and set up. **Figure S6.** In situ sampling procedure of abalone for each algal treatment. **Figure S7.** Experimental procedure for DNA extraction and library preparation for studying the abalone digestive microbiota. (DOCX 1944 kb)

Additional file 2: **Table S1.** Wilcoxon test to compare Shannon or Simpson indices between algal diets for the whole data set or between sampling dates or seasons for each algal diet (multiple comparisons were corrected using FDR). (XLSX 22 kb)

Additional file 3: **Table S2.** R value of the ANOSIM calculated for the whole dataset and for each algal diet dataset separately between sample groups according to season, sampling date, or algal diet. Numbers in bold indicate the significance of the comparison after Bonferroni correction. (XLSX 15 kb)

Additional file 4: **Table S3.** Number of annotated genes for each glycoside hydrolase, polysaccharide lyase, and sulfatase family in all *Vibrio*, *Mycoplasma*, *Polaribacter*, *Roseobacter*, and *Psychromonas* genomes available at http://www.cazy.org/ and/or at http://abims.sb-roscoff.fr/sulfatlas/ on 19 May 2017 and in *Zobellia galactanivorans* DsijT (Barbeyron et al. 2017). GH, Glycoside hydrolases; PL, Polysaccharide lyases, CAZyme, Carbohydrate active enzyme. Sulfatase gene counts are indicated when available in the SulfAtlas database. (XLSX 160 kb)

Additional file 5: **Table S4.** Genes belonging to families of glycoside hydrolases, polysaccharide lyases, or sulfatases and genes putatively involved in the pyruvate fermentation to acetate pathway in the genomes of *Psychrilyobacter atlanticus* DSM19335 (A), *Vibrio halioticoli* NBRC 102217 (B), and *Mycoplasma mobile* 163 K (C), when present. Annotations include gene identifiers (locus tags), closest characterized homologs with their UniProtKB ID number when available, EC numbers, and information on their functioning. The percentage of amino-acid sequence identity is indicated in parentheses. (XLSX 66 kb)

Additional file 6: **Table S5.** Measures of abalone characteristics and algal composition from April 2012 to January 2013. See supplementary text for details. GDG, gonado-digestive gland; DG, digestive gland. (XLSX 33 kb)

Additional file 7: **Table S6.** Fasta sequences of the pyruvate to acetate formation pathways, I, II, and IV, commonly found in *Bacteria* according to MetaCyc (https://metacyc.org/META/NEW-IMAGE?object=Super-Pathways&detail-level=3). (DOCX 61 kb)

Additional file 8: Supplementary text. Supplementary information on methods used and results analysed for the study. (DOCX 43 kb)
