# Supplementary material for: Seasonal and algal diet-driven patterns of the digestive microbiota of the European abalone Haliotis tuberculata, a generalist marine herbivore
Source: Microbiome. 2018 Mar 27;6:60. doi: 10.1186/s40168-018-0430-7 (PMC5870069; doi:10.1186/s40168-018-0430-7)
Supplement: Supplementary file 1 — Figure S1. Alpha-diversity of the digestive microbiota of abalone, as described by the Shannon (left panels) and Simpson (right panels) indices. Figure S2. Fluctuations of the digestive microbiota between consecutive dates over 1 year. Figure S3. Relative abundance and taxonomical composition of the digestive microbiota at the phylum level for abalone fed on Palmaria palmata (A), Laminaria digitata (B), Ulva lactuca (C), and Saccharina latissima (D). Figure S4. Ecological patterns of the digestive microbiota explained by a model of contextual parameters. Figure S5. Experimental site and set up. Figure S6. In situ sampling procedure of abalone for each algal treatment. Figure S7. Experimental procedure for DNA extraction and library preparation for studying the abalone digestive microbiota. (DOCX 1944 kb) [file 40168_2018_430_MOESM1_ESM.docx]

**Seasonal and algal diet-driven patterns of the digestive microbiota of the European abalone *Haliotis tuberculata*, a generalist marine herbivore**

Angélique Gobet^1*^, Laëtitia Mest^1^, Morgan Perennou^2^, Simon Dittami^1^, Claire Caralp^3^, Céline Coulombet^4^, Sylvain Huchette^4^, Sabine Roussel^3^, Gurvan Michel^1*^ and Catherine Leblanc^1*^

**Additional file 1**

**Figure S1. Alpha-diversity of the digestive microbiota of abalone, as described by the Shannon (left panels) and Simpson (right panels) indices.**

**Figure S2. Fluctuations of the digestive microbiota between consecutive dates over one year.**

**Figure S3. Relative abundance and taxonomical composition of the digestive microbiota at the phylum level for abalone fed on *Palmaria* *palmata* (A), *Laminaria* *digitata* (B), *Ulva* *lactuca* (C) and *Saccharina* *latissima* (D).**

**Figure S4. Ecological patterns of the digestive microbiota explained by a model of contextual parameters.**

**Figure S5. Experimental site and set up.**

**Figure S6. *In situ* sampling procedure of abalone for each algal treatment.**

**Figure S7. Experimental procedure for DNA extraction and library preparation for studying the abalone digestive microbiota.**


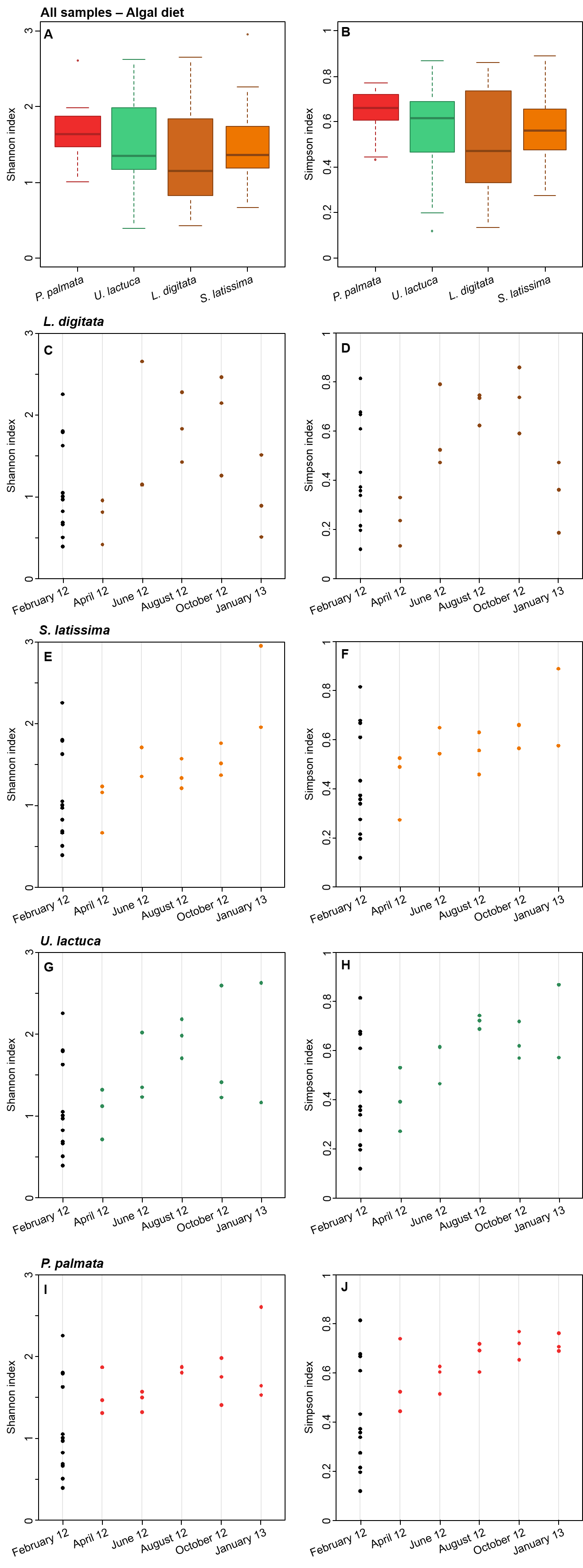


**Figure S1. Alpha-diversity of the digestive microbiota of abalone, as described by the Shannon (left panels) and Simpson (right panels) indices.** Alpha-diversity indices were calculated when the whole dataset was considered, according to each algal diet (**A**-**B**), and for each algal diet dataset individually, over one year (**C**-**J**). Alpha-diversity indices were calculated from an average of 1,000 subsamplings to a total of 12,997 sequences per sample. February 2012 corresponds to the microbial community in the 12 cages at the beginning of the experiment (T0, black dots). Mean difference was tested for significance after FDR correction (*P* < 0,048, Wilcoxon test).


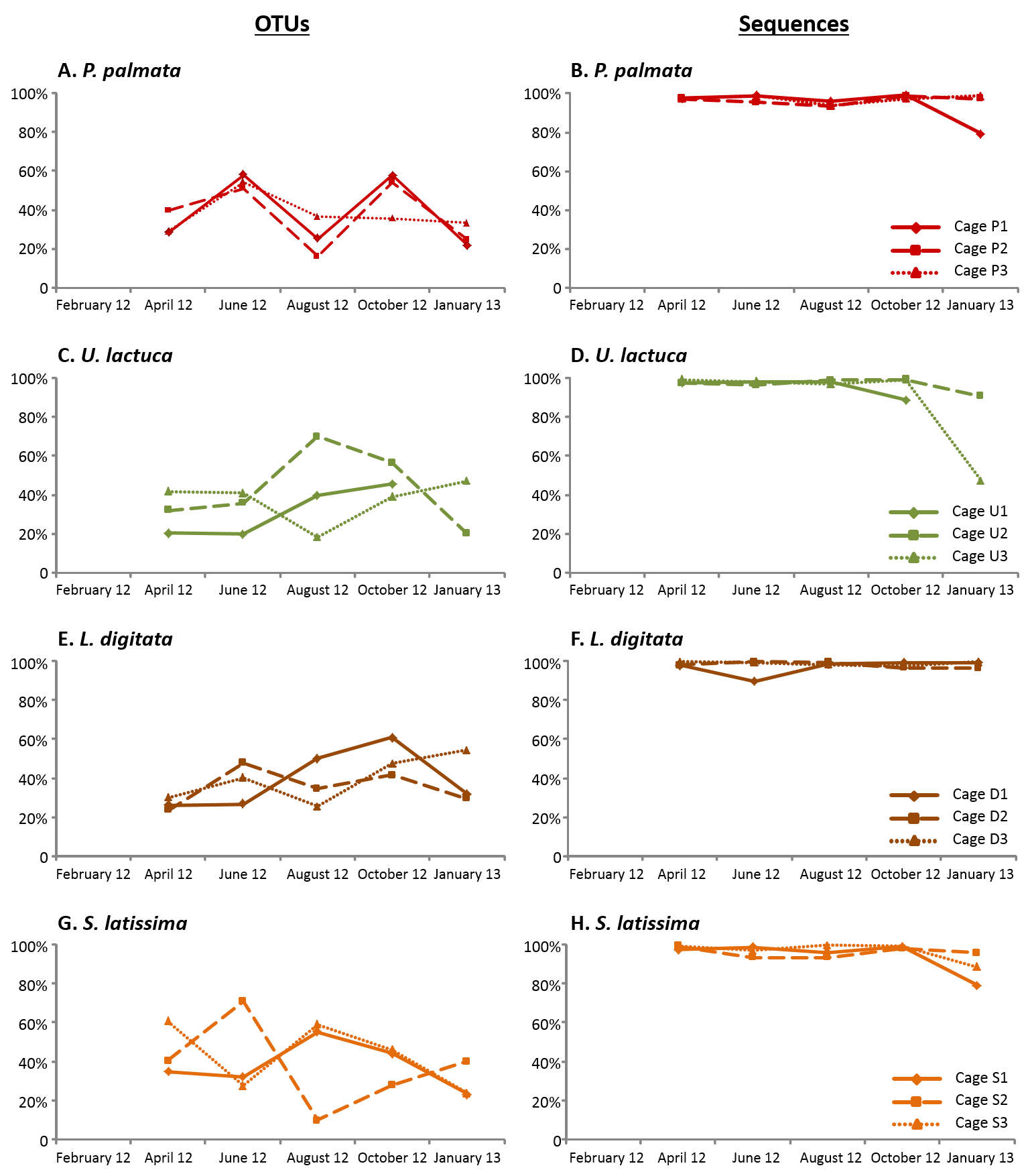


**Figure S2. Fluctuations of the digestive microbiota between consecutive dates over one year.** Proportions of OTUs (**A**, **C**, **E**, **G**) and of sequence abundance (**B**, **D**, **F**, **H**) shared between 2 consecutive sampling dates in each cage for a given algal diet: *P. palmata* (**A**, **B**), *U. lactuca* (**C**, **D**), *L. digitata* (**E**, **F**), *S. latissima* (**G**, **H**). In the case of the cage U1, the sample from January 2013 is missing.

**
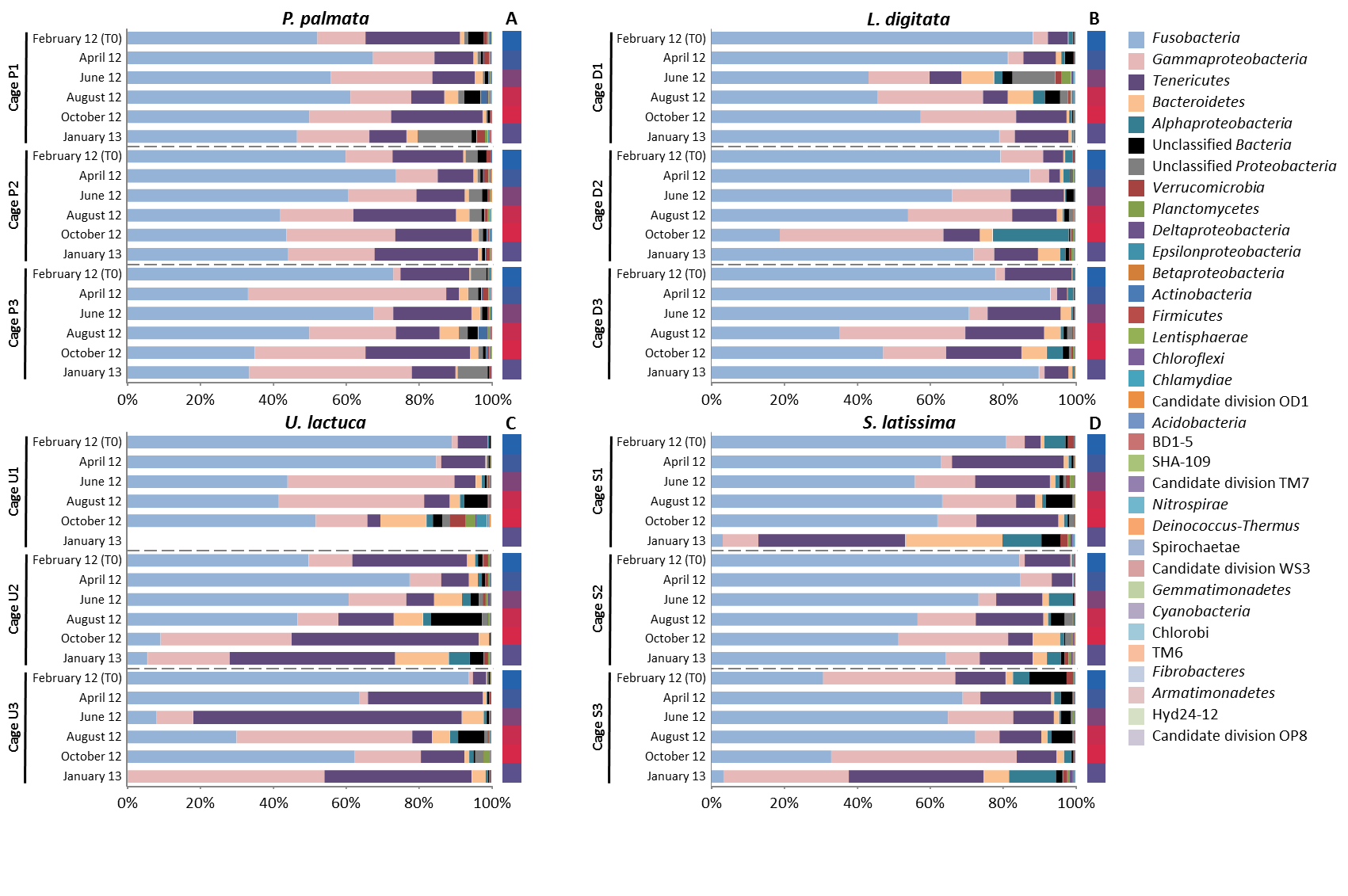
**

**Figure S3. Relative abundance and taxonomical composition of the digestive microbiota at the phylum level for abalone fed on *Palmaria* *palmata* (A), *Laminaria* *digitata* (B), *Ulva* *lactuca* (C) and *Saccharina* *latissima* (D).** Taxonomical composition is represented for each cage triplicate over one year. Water temperature is indicated on the right side of each panel (Values varying from 10°C (blue) to 15°C (red)). In **C**, the sample from cage U1 in January 2013 is missing. The phylum *Proteobacteria* was split into its corresponding classes. For unclassified phyla, phyla were assigned to the lowest taxonomic level identified.


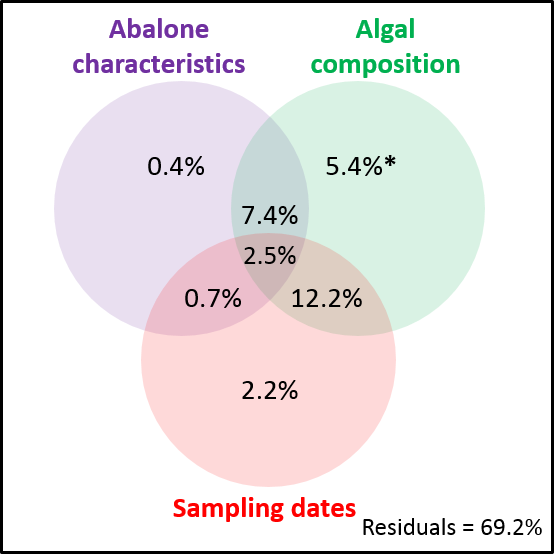


**Figure S4. Ecological patterns of the digestive microbiota explained by a model of contextual parameters.** For each argument of the model considered, parameters were chosen *a priori* using forward selection. Abalone characteristics included daily growth rate measured as shell length, abalone weight/size ratio, and digestive gland weight. Algal composition included proportions of soluble carbohydrates in algal dry weight, % dry algal matter, total (methionine, arginine, valine, leucine) and free aminoacids (lysine, threonine). Proportions of soluble carbohydrates in algal dry weight and % dry algal matter significantly showed seasonal variation (as tested using the Friedman test with post-hoc test after Nemenyi, Roussel, personal communication). Water temperature was not included in the model as it was highly collinear to the three other arguments. The star indicates *P* < 0.05.


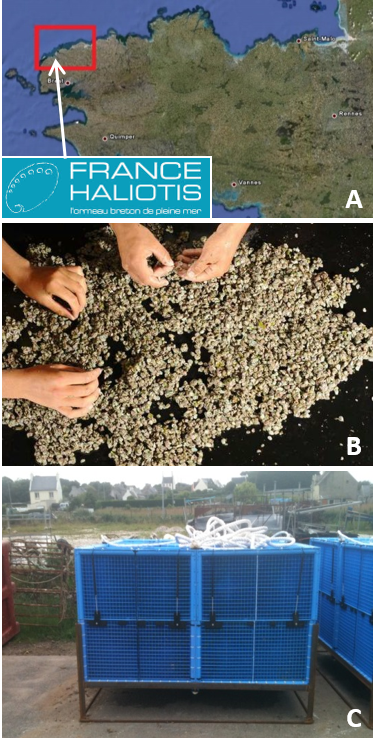


**Figure S5. Experimental site and set up.** The abalone farm France Haliotis is located in the North Western part of France, in Brittany (**A**). Random collection of 1,000 abalone juveniles before breeding in cages in the sea (**B**). Abblox structure composed of 4 cages of 1 m^3^ with a squared mesh size of 5 mm, allowing water flow and ensuring no significant input from outside of the cages (C). Photos A, B, C from GoogleMaps and France Haliotis.


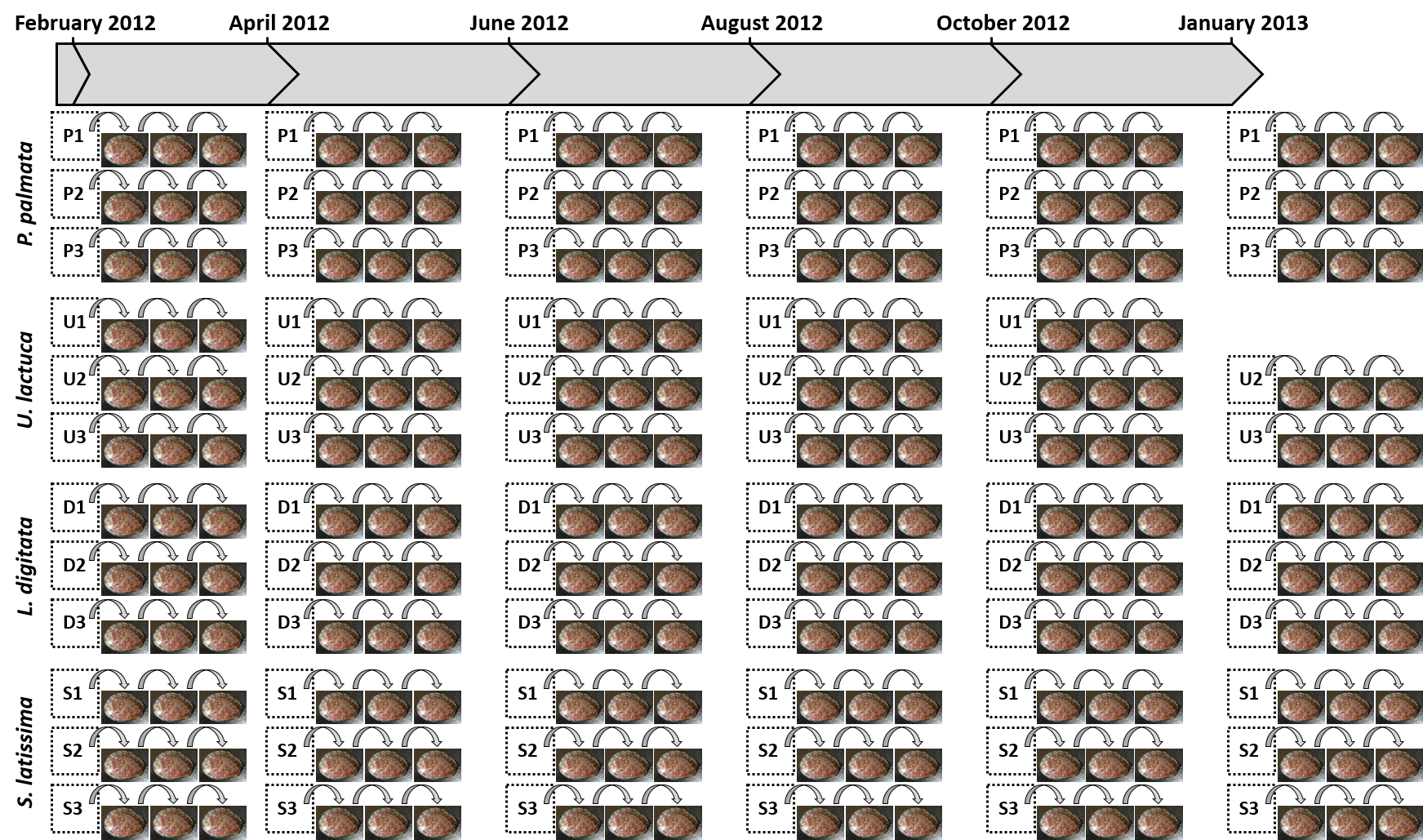


**Figure S6. *In situ* sampling procedure of abalone for each algal treatment.** From February 2012 to January 2013, every 2 to 3 months, 3 abalone were sampled from each of the 12 cages (cage triplicates for each of the four algal diets). This resulted in a total of 216 abalone sampled (3 abalone x 3 cages x 4 algal diets x 6 sampling dates). Samples from the cage U1 taken in January 2013 were removed from the figure as the construction of the corresponding DNA library was unsuccessful.


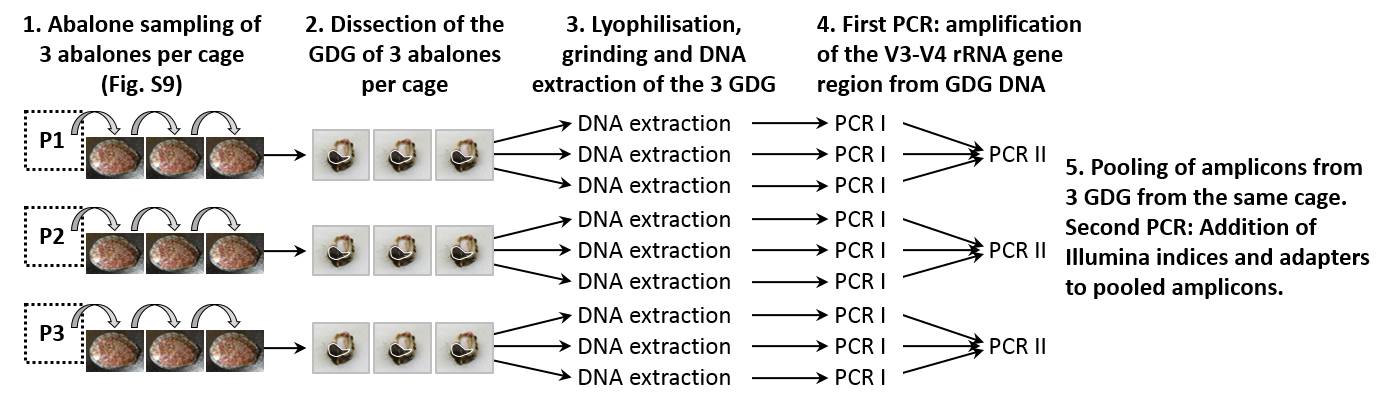


**Figure S7. Experimental procedure for DNA extraction and library preparation for studying the abalone digestive microbiota.** Three abalone were sampled per cage and their gonado-digestive gland (GDG) was successively dissected. The GDG were then freeze-dried and ground before DNA extraction. A first PCR on DNA from each GDG sample (PCR I) allowed amplifying the V3-V4 region of the 16S rRNA gene targeting *Bacteria*. The resulting amplicons from the 3 GDG samples from the same cage were then pooled before a second PCR allowing addition of Illumina indices and adapters (PCR II). A total of 72 libraries were then sequenced using the Illumina technology on a MiSeq sequencer.
