# Supplementary material for: Seasonal and algal diet-driven patterns of the digestive microbiota of the European abalone Haliotis tuberculata, a generalist marine herbivore
Source: Microbiome. 2018 Mar 27;6:60. doi: 10.1186/s40168-018-0430-7 (PMC5870069; doi:10.1186/s40168-018-0430-7)
Supplement: Supplementary file 7 — Table S6. Fasta sequences of the pyruvate to acetate formation pathways, I, II, and IV, commonly found in Bacteria according to MetaCyc (https://metacyc.org/META/NEW-IMAGE?object=Super-Pathways&detail-level=3). (DOCX 61 kb) [file 40168_2018_430_MOESM7_ESM.docx]

**Table S6.** Fasta sequences of the pyruvate to acetate formation pathways I, II and IV, commonly found in Bacteria according to MetaCyc (https://metacyc.org/META/NEW-IMAGE?object=Super-Pathways&detail-level=3).

*1. Acetate formation pathway I.*

>sp|P94692|POR_DESAF Pyruvate synthase OS=Desulfovibrio africanus GN=por PE=1 SV=1

MGKKMMTTDGNTATAHVAYAMSEVAAIYPITPSSTMGEEADDWAAQGRKNIFGQTLTIRE

MQSEAGAAGAVHGALAAGALTTTFTASQGLLLMIPNMYKISGELLPGVFHVTARAIAAHA

LSIFGDHQDIYAARQTGFAMLASSSVQEAHDMALVAHLAAIESNVPFMHFFDGFRTSHEI

QKIEVLDYADMASLVNQKALAEFRAKSMNPEHPHVRGTAQNPDIYFQGREAANPYYLKVP

GIVAEYMQKVASLTGRSYKLFDYVGAPDAERVIVSMGSSCETIEEVINHLAAKGEKIGLI

KVRLYRPFVSEAFFAALPASAKVITVLDRTKEPGAPGDPLYLDVCSAFVERGEAMPKILA

GRYGLGSKEFSPAMVKSVYDNMSGAKKNHFTVGIEDDVTGTSLPVDNAFADTTPKGTIQC

QFWGLGADGTVGANKQAIKIIGDNTDLFAQGYFSYDSKKSGGITISHLRFGEKPIQSTYL

VNRADYVACHNPAYVGIYDILEGIKDGGTFVLNSPWSSLEDMDKHLPSGIKRTIANKKLK

FYNIDAVKIATDVGLGGRINMIMQTAFFKLAGVLPFEKAVDLLKKSIHKAYGKKGEKIVK

MNTDAVDQAVTSLQEFKYPDSWKDAPAETKAEPMTNEFFKNVVKPILTQQGDKLPVSAFE

ADGRFPLGTSQFEKRGVAINVPQWVPENCIQCNQCAFVCPHSAILPVLAKEEELVGAPAN

FTALEAKGKELKGYKFRIQINTLDCMGCGNCADICPPKEKALVMQPLDTQRDAQVPNLEY

AARIPVKSEVLPRDSLKGSQFQEPLMEFSGACSGCGETPYVRVITQLFGERMFIANATGC

SSIWGASAPSMPYKTNRLGQGPAWGNSLFEDAAEYGFGMNMSMFARRTHLADLAAKALES

DASGDVKEALQGWLAGKNDPIKSKEYGDKLKKLLAGQKDGLLGQIAAMSDLYTKKSVWIF

GGDGWAYDIGYGGLDHVLASGEDVNVFVMDTEVYSNTGGQSSKATPTGAVAKFAAAGKRT

GKKDLARMVMTYGYVYVATVSMGYSKQQFLKVLKEAESFPGPSLVIAYATCINQGLRKGM

GKSQDVMNTAVKSGYWPLFRYDPRLAAQGKNPFQLDSKAPDGSVEEFLMAQNRFAVLDRS

FPEDAKRLRAQVAHELDVRFKELEHMAATNIFESFAPAGGKADGSVDFGEGAEFCTRDDT

PMMARPDSGEACDQNRAGTSEQQGDLSKRTKK

>sp|Q51804|PORA_PYRFU Pyruvate synthase subunit PorA OS=Pyrococcus furiosus (strain ATCC 43587 / DSM 3638 / JCM 8422 / Vc1) GN=porA PE=1 SV=2

MPIRKVMKANEAAAWAAKLAKPKVIAAFPITPSTLIPEKISEFVANGELDAEFIKVESEH

SAISACVGAAAAGVRTFTATASQGLALMHEILFIAAGMRLPIVMAIGNRALSAPINIWND

WQDTISQRDTGWMQFYAENNQEALDLILIAYKVAEDERVLLPAMVGFDAFILTHTVEPVE

IPDQEVVDEFLGEYEPKHAYIDPARPITQGSLAFPAHYMESRYTVWEAMERAKKVIDEAF

AEFEKKFGRKYQKIEEYKTEDADIIFVTMGSLAGTLKEWIDKKREEGYKVGAAKITVYRP

FPVEEIRELAKKAKVLAFLEKNITIGLYGAVFTDASAALINESEKPLMVDFIVGLGGRDV

TFNQLDEALEIAEKALKEGKVENPINWIGLRWELVK

>sp|P80521|PORA_METBF Pyruvate synthase subunit PorA OS=Methanosarcina barkeri (strain Fusaro / DSM 804) GN=porA PE=1 SV=2

MIDPAYRKKMVVVEGSYAVAHSAKVCRPNVISAYPITPQTHIVEHLSQFMADGEIPNCEY

VNVEAEFSAISALIGASAVGARTYSATTSQGLLLMHEALFNTSGMRLPVVMTVANRAVSA

PINIWNDHQDAIAQRDTGWMQLYVEDVQEACDTLPQLYKIAEDNEIMVPGMVCMDGFILS

HVYEPVVLLEQDLTDNFLPPFQPEDILDPEDPKTFGAFASPDTYEEFRYLHEQAMQKALP

KIEATAKEFEEVYGRYHGGLIDGYMLDDAEIVVMAMGSILGTVKDVVDKYRAKGEKIGVL

KVRSFRPFPKEQICKAVKNAHAVVVLDKNISIGTNEGALFTETKSCLYNSKVRVPVIGYT

IGHGGRDIPVESIAKVIEETKKVAKSGITIESQFMDLKEELL

>sp|P80900|PORA_METTM Pyruvate synthase subunit PorA OS=Methanothermobacter marburgensis (strain DSM 2133 / 14651 / NBRC 100331 / OCM 82 / Marburg) GN=porA PE=1 SV=2

MVLKVISANQAVAEAAKLAKPKVIPVYPITPQTSISEYLAKYVADGELDAEYIRVESEHS

AMSACVGASGAGVRVFTATSSQGLALMHEIVYAAAGLRNPIVMANANRALSAPLSIWNDQ

QDSIAERDSGWMQIYAESGQEALDSVLLSYRVSEDRDVLLPSMVCLDGFILTHTVEPVDI

PSQDEVDTFLPEFQPQAVLDPDEPMSLGTFTDPNYYMEARYEVERAMERSRKVIAKACQE

FSEMFRREYGFVEDYRCEDAEIILVAMGSVCSTLREVIDDMRDEGKPVGLLKVRIHRPFP

AEEIKKAVSNAHKIAVLDKNITFSVGGALHTELKALLPDKEVYGFIVGLGGRDITPEHIM

EIVRKTENPERTVSWIGLKEESQ

>sp|O05651|PORA_THEMA Pyruvate synthase subunit PorA OS=Thermotoga maritima (strain ATCC 43589 / MSB8 / DSM 3109 / JCM 10099) GN=porA PE=1 SV=2

MERVVERVAVTGAEAVANAMRQIEPDVVAAYPITPQTPIVEYFARFVADGVVRTEMIPVE

SEHSAMSAVVGAAAAGARAMTATSANGLALMHEIVYIAASYRLPIVMPVVNRALSGPINI

HCDHSDAMAERDSGWIQLFAETNQEAYDFTILAVRLAEHEDVRLPVMVNLDGFILSHGVE

PVEFYPDELVKKFVGELKPMYPLLDTEHPVTWGPLDLYDYYFEHKRQQIEAMENVKKVFP

EIAKEFEETFGRKYWFVEPYRMEDAEHVMVALGSTNSTIKYVVDELREEGYKVGSLKIWM

FRPFPKEQLQELLNGRKSVVVLDRAVSFGAEAPLYEAVKSALYEVAARPMLGSYVYGLGG

RDIKPEHIRKAFEDAINGNLIADEQRYLGLRE

>sp|P80522|PORB_METBF Pyruvate synthase subunit PorB OS=Methanosarcina barkeri (strain Fusaro / DSM 804) GN=porB PE=1 SV=3

MSKTAPKTYITSGHSGCAGCCDAFAAKFTLMGAGPNTIVINPTGCLEVMSTPFPYSSWQV

PWIHSLFENAGAVASGVEAALKALGKKDDVKVVSIGGDGSTMDIGLGALSGAFERGHDFT

YVCMDNEAYMNTGVQRSSGTPFDASTTTTPAGKVSFGNPRPKKNMPAIMAAHGSPYVATT

SIGFPRDMIRKVKKATEIVGPTYIHAQAPCPTGWGFDTSKTLEIAKLAVETCLWPMYEME

NGEITQVRKVKNPRPVEEYLRAQKRFKHLFTMEGGEEEIKKIQAIADWNIKHFELQ

>sp|P80901|PORB_METTM Pyruvate synthase subunit PorB OS=Methanothermobacter marburgensis (strain DSM 2133 / 14651 / NBRC 100331 / OCM 82 / Marburg) GN=porB PE=1 SV=2

MKIPEEEFLAPGHRGCAGCGATVGVRLALKVLGKNTVAVSSTGCLEVITTPYPETAWEIP

WIHVAFENAAAVASGVERALRARGRGEVNVVAFAGDGGTADIGLQSLSGAMERGHNIIYI

CYDNEAYMNTGIQRSASTPYGASTTTSPHGKESFGEDRPKKNMPLIMAAHGVPYVATASI

SYPEDFMEKVRKARDIEGPAYIHLHQPCTTGWGFDPSKTVELGRLAVETGSWILYEIEDG

DFRVTYRPVQRKPVEEYLNAQKRFRHLTEEQKAKIQEYVDSVCQELRI

>sp|Q51805|PORB_PYRFU Pyruvate synthase subunit PorB OS=Pyrococcus furiosus (strain ATCC 43587 / DSM 3638 / JCM 8422 / Vc1) GN=porB PE=1 SV=3

MAVRKPPITTREYWAPGHAACAGCGCATALRLATKALSEAMEEKYGDPNAFAIAHATGCM

EVVSAVFPYTAWKAPWIHVAFENAAAVASGIEAAWKKLGRKGKILAIGGDGGTADIGLQA

LSGMLERWHNVLYLMYDNEAYMNTGIQRSSSTPYGAWTTTSPPGKYSVGEDKPKKWVALI

AAAHQIPYVATASIGNPLDFVRKIKKAGKIDGPAFVQVLCTCPTGWRSPLEKGVEIARLA

IETGIWPLFEIENGDIWNIKIQPPGGGAKVYKEGNRVVRIEFKKPIEEYLKLQGRFKHLF

KRPEAIEELRNQVKAMWKVLGVEAILPRPEE

>sp|Q56317|PORB_THEMA Pyruvate synthase subunit PorB OS=Thermotoga maritima (strain ATCC 43589 / MSB8 / DSM 3109 / JCM 10099) GN=porB PE=1 SV=3

MPVNIKQLAQEFDKKEIGITQGHRLCPGCGAPITVKFVMMIARHLGYEPVVGLATGCLEV

STSIYPYTAWSVPYIHNAFENVAATMSGVETAYKALKNKGKIPEDKKYAFIAFGGDGGTY

DIGLQSLSGMLERGHKVLYVLYDNEGYMNTGNQRSGSTPPGSDTTTAPVGKKLPGKVQLK

KNIVEIVAAHENVYAATASLSEPMDFFAKVEKALNFDGPSFLAVFSPCVRFWRVNDDKTV

EISKLAVETKYWPLYEVERGVYRVTRKPRQFKPVEEFLKAQGRFRKLLSRPDAKEIVDEL

QEYVDRRWERLLTLEEVTKDKPIR

>sp|P80902|PORC_METTM Pyruvate synthase subunit PorC OS=Methanothermobacter marburgensis (strain DSM 2133 / 14651 / NBRC 100331 / OCM 82 / Marburg) GN=porC PE=1 SV=2

MIEIRFHGRGGQGAVTAAEILAKAAFEDGKYSQAFPFFGVERRGAPVMAFTRINDEPIRR

RYQVYNPDYVVVLDEGLVDVVDVFSGLKEDGVVLLNTAGTFTSENAKIHTIDATGIALEN

LGRPIVNTVMLGAFAGVTGLVSIDSLIKIIKETFPGKIGDKNAEAARIAYEKMKHSG

>sp|P80523|PORC_METBF Pyruvate synthase subunit PorC OS=Methanosarcina barkeri (strain Fusaro / DSM 804) GN=porC PE=1 SV=2

MKEIRIHGRGGQGSVTAAEMLSVAAFEDGKFSQAFPAFGVERRGAPVQAFTRINNNPIRL

RSQVYTPDYVIVQDATLLETVDVASGVKDDGIIIVNTTENPESLKLNTKARVMTVDATKV

AMDIIGVPIVNTVLLGAFAGATGEINVESIQHAIRARFSGKVGEKNANAIQKAYKLIRGE

EA

>sp|Q51799|PORC_PYRFU Pyruvate/ketoisovalerate oxidoreductases common subunit gamma OS=Pyrococcus furiosus (strain ATCC 43587 / DSM 3638 / JCM 8422 / Vc1) GN=porG PE=1 SV=1

MIEVRFHGRGGQGAVTAANILAEAAFLEGKYVQAFPFFGVERRGAPVTAFTRIDNKPIRI

KTQIYEPDVVVVLDPSLLDAVDVTAGLKDEGIVIVNTEKSKEEVLEKLKKKPKKLAIVDA

TTIALEILGLPITNTAILGAVAKATGLVKIESIEEAIKDTFSGELGEKNARAAREAYEKT

EVFEL

>sp|O05650|PORC_THEMA Pyruvate synthase subunit PorC OS=Thermotoga maritima (strain ATCC 43589 / MSB8 / DSM 3109 / JCM 10099) GN=porC PE=1 SV=4

MPVAKKYFEIRWHGRAGQGAKSASQMLAEAALEAGKYVQAFPEYGAERTGAPMRAFNRIG

DEYIRVRSAVENPDVVVVIDETLLSPAIVEGLSEDGILLVNTVKDFEFVRKKTGFNGKIC

VVDATDIALQEIKRGIPNTPMLGALVRVTGIVPLEAIEKRIEKMFGKKFPQEVIDANKRA

LRRGYEEVKCSE

>sp|P84820|PORC_THELN Pyruvate/ketoisovalerate oxidoreductases common subunit gamma OS=Thermococcus litoralis (strain ATCC 51850 / DSM 5473 / JCM 8560 / NS-C) GN=porG PE=1 SV=2

MIEIRFHGRGGQGAVTAANILAEAAFLEGKYVQAFPFFGVERRGAPVTAFTRIDDKPIRI

KTQIYEPDVVVVLDPSLLDTVDVTAGLKEGGMVIVNTEKTKEEVLEKLKKKPAKLALVDA

TTIALEILGLPITNTSILGAVAKATGIVKIESVEEAIKDTFSGELGKKNAKAAREAFEKT

VVYEL

>sp|P84819|PORC_PYREN Pyruvate/ketoisovalerate oxidoreductases common subunit gamma (Fragment) OS=Pyrococcus endeavori GN=porG PE=1 SV=1

MIEIRFHGRGGQGAV

>sp|P0A9M8|PTA_ECOLI Phosphate acetyltransferase OS=Escherichia coli (strain K12) GN=pta PE=1 SV=2

MSRIIMLIPTGTSVGLTSVSLGVIRAMERKGVRLSVFKPIAQPRTGGDAPDQTTTIVRAN

SSTTTAAEPLKMSYVEGLLSSNQKDVLMEEIVANYHANTKDAEVVLVEGLVPTRKHQFAQ

SLNYEIAKTLNAEIVFVMSQGTDTPEQLKERIELTRNSFGGAKNTNITGVIVNKLNAPVD

EQGRTRPDLSEIFDDSSKAKVNNVDPAKLQESSPLPVLGAVPWSFDLIATRAIDMARHLN

ATIINEGDINTRRVKSVTFCARSIPHMLEHFRAGSLLVTSADRPDVLVAACLAAMNGVEI

GALLLTGGYEMDARISKLCERAFATGLPVFMVNTNTWQTSLSLQSFNLEVPVDDHERIEK

VQEYVANYINADWIESLTATSERSRRLSPPAFRYQLTELARKAGKRIVLPEGDEPRTVKA

AAICAERGIATCVLLGNPAEINRVAASQGVELGAGIEIVDPEVVRESYVGRLVELRKNKG

MTETVAREQLEDNVVLGTLMLEQDEVDGLVSGAVHTTANTIRPPLQLIKTAPGSSLVSSV

FFMLLPEQVYVYGDCAINPDPTAEQLAEIAIQSADSAAAFGIEPRVAMLSYSTGTSGAGS

DVEKVREATRLAQEKRPDLMIDGPLQYDAAVMADVAKSKAPNSPVAGRATVFIFPDLNTG

NTTYKAVQRSADLISIGPMLQGMRKPVNDLSRGALVDDIVYTIALTAIQSAQQQ

>sp|P38503|PTAS_METTE Phosphate acetyltransferase OS=Methanosarcina thermophila GN=pta PE=1 SV=3

MVTFLEKISERAKKLNKTIALPETEDIRTLQAAAKILERGIADIVLVGNEADIKALAGDL

DLSKAKIVDPKTYEKKDEYINAFYELRKHKGITLENAAEIMSDYVYFAVMMAKLGEVDGV

VSGAAHSSSDTLRPAVQIVKTAKGAALASAFFIISVPDCEYGSDGTFLFADSGMVEMPSV

EDVANIAVISAKTFELLVQDVPKVAMLSYSTKGSAKSKLTEATIASTKLAQELAPDIAID

GELQVDAAIVPKVAASKAPGSPVAGKANVFIFPDLNCGNIAYKIAQRLAKAEAYGPITQG

LAKPINDLSRGCSDEDIVGAVAITCVQAAAQDK

>sp|P39646|PTAS_BACSU Phosphate acetyltransferase OS=Bacillus subtilis (strain 168) GN=pta PE=1 SV=3

MADLFSTVQEKVAGKDVKIVFPEGLDERILEAVSKLAGNKVLNPIVIGNENEIQAKAKEL

NLTLGGVKIYDPHTYEGMEDLVQAFVERRKGKATEEQARKALLDENYFGTMLVYKGLADG

LVSGAAHSTADTVRPALQIIKTKEGVKKTSGVFIMARGEEQYVFADCAINIAPDSQDLAE

IAIESANTAKMFDIEPRVAMLSFSTKGSAKSDETEKVADAVKIAKEKAPELTLDGEFQFD

AAFVPSVAEKKAPDSEIKGDANVFVFPSLEAGNIGYKIAQRLGNFEAVGPILQGLNMPVN

DLSRGCNAEDVYNLALITAAQAL

>sp|Q8ZND6|PTA_SALTY Phosphate acetyltransferase OS=Salmonella typhimurium (strain LT2 / SGSC1412 / ATCC 700720) GN=pta PE=1 SV=1

MSRIIMLIPTGTSVGLTSVSLGVIRAMERKGVRLSVFKPIAQPRAGGDAPDQTTTIVRAN

STLPAAEPLKMSHVESLLSSNQKDVLMEEIIANYHANTKDAEVVLVEGLVPTRKHQFAQS

LNYEIAKTLNAEIVFVMSQGTDTPEQLNERIELTRSSFGGAKNTNITGVIINKLNAPVDE

QGRTRPDLSEIFDDSSKAQVIKIDPAKLQESSPLPVLGAVPWSFDLIATRAIDMARHLNA

TIINEGDIKTRRVKSVTFCARSIPHMLEHFRAGSLLVTSADRPDVLVAACLAAMNGVEIG

ALLLTGGYEMDARISKLCERAFATGLPVFMVNTNTWQTSLSLQSFNLEVPVDDHERIEKV

QEYVANYVNAEWIESLTATSERSRRLSPPAFRYQLTELARKAGKRVVLPEGDEPRTVKAA

AICAERGIATCVLLGNPDEINRVAASQGVELGAGIEIVDPEVVRESYVARLVELRKSKGM

TEPVAREQLEDNVVLGTLMLEQDEVDGLVSGAVHTTANTIRPPLQLIKTAPGSSLVSSVF

FMLLPEQVYVYGDCAINPDPTAEQLAEIAIQSADSAIAFGIEPRVAMLSYSTGTSGAGSD

VEKVREATRLAQEKRPDLMIDGPLQYDAAVMADVAKSKAPNSPVAGRATVFIFPDLNTGN

TTYKAVQRSADLISIGPMLQGMRKPVNDLSRGALVDDIVYTIALTAIQASQQQQ

>sp|P99092|PTAS_STAAN Phosphate acetyltransferase OS=Staphylococcus aureus (strain N315) GN=pta PE=1 SV=1

MADLLNVLKDKLSGKNVKIVLPEGEDERVLTAATQLQATDYVTPIVLGDETKVQSLAQKL

DLDISNIELINPATSELKAELVQSFVERRKGKATEEQAQELLNNVNYFGTMLVYAGKADG

LVSGAAHSTGDTVRPALQIIKTKPGVSRTSGIFFMIKGDEQYIFGDCAINPELDSQGLAE

IAVESAKSALSFGMDPKVAMLSFSTKGSAKSDDVTKVQEAVKLAQQKAEEEKLEAIIDGE

FQFDAAIVPGVAEKKAPGAKLQGDANVFVFPSLEAGNIGYKIAQRLGGYDAVGPVLQGLN

SPVNDLSRGCSIEDVYNLSIITAAQALQ

>sp|Q6GJ80|PTAS_STAAR Phosphate acetyltransferase OS=Staphylococcus aureus (strain MRSA252) GN=pta PE=1 SV=1

MADLLNVLKDKLSGKNVKIVLPEGEDERVLTAATQLQATDYVTPIVLGDETKVQSLAQKL

NLDISNIELINPATSELKAELVQSFVERRKGKTTEEQAQELLNNVNYFGTMLVYAGKADG

LVSGAAHSTGDTVRPALQIIKTKPGVSRTSGIFFMIKGDEQYIFGDCAINPELDSQGLAE

IAVESAKSALSFGMDPKVAMLSFSTKGSAKSDDVTKVQEAVKLAQQKAEEEKLEAIIDGE

FQFDAAIVPGVAEKKAPGAKLQGDANVFVFPSLEAGNIGYKIAQRLGGYDAVGPVLQGLN

SPVNDLSRGCSIEDVYNLSFITAAQALQ

>sp|Q9X0L4|PTAS_THEMA Phosphate acetyltransferase OS=Thermotoga maritima (strain ATCC 43589 / MSB8 / DSM 3109 / JCM 10099) GN=pta PE=1 SV=1

MFLEKLVEMARGKGKKLAVAAANDDHVIEAVYRAWRERVCEPVLFGPEEEITRIIEELVP

EWKNPQIIDCPPEEAGRLAVEAVSKGECDFLMKGKIKTGDLMKIYLDERYGLRTGKTMAM

VSVMEIPDFPRPLIISDPGMLISPTLEQKVDMIEHCVRVANVMGLETPKVAVVGAIEVVN

PKMPITMEAAILSKMNQRGQIKGCIVDGPFALDNVVSEEAAKKKGIQSPVAGKADILILP

DIEAANILYKALVFLAKAKSASTILGGKVPVVLTSRADSEETKFYSIALSAVFA

>sp|P9WHP1|PTA_MYCTU Phosphate acetyltransferase OS=Mycobacterium tuberculosis (strain ATCC 25618 / H37Rv) GN=pta PE=1 SV=1

MADSSAIYLAAPESQTGKSTIALGLLHRLTAMVAKVGVFRPITRLSAERDYILELLLAHT

SAGLPYERCVGVTYQQLHADRDDAIAEIVDSYHAMADECDAVVVVGSDYTDVTSPTELSV

NGRIAVNLGAPVLLTVRAKDRTPDQVASVVEVCLAELDTQRAHTAAVVANRCELSAIPAV

TDALRRFTPPSYVVPEEPLLSAPTVAELTQAVNGAVVSGDVALREREVMGVLAAGMTADH

VLERLTDGMAVITPGDRSDVVLAVASAHAAEGFPSLSCIVLNGGFQLHPAIAALVSGLRL

RLPVIATALGTYDTASAAASARGLVTATSQRKIDTALELMDRHVDVAGLLAQLTIPIPTV

TTPQMFTYRLLQQARSDLMRIVLPEGDDDRILKSAGRLLQRGIVDLTILGDEAKVRLRAA

ELGVDLDGATVIEPCASELHDQFADQYAQLRKAKGITVEHAREIMNDATYFGTMLVHNCH

ADGMVSGAAHTTAHTVRPALEIIKTVPGISTVSSIFLMCLPDRVLAYGDCAIIPNPTVEQ

LADIAICSARTAAQFGIEPRVAMLSYSTGDSGKGADVDKVRAATELVRAREPQLPVEGPI

QYDAAVEPSVAATKLRDSPVAGRATVLIFPDLNTGNNTYKAVQRSAGAIAIGPVLQGLRK

PVNDLSRGALVDDIVNTVAITAIQAQGVHE

>sp|Q9I5A5|PTA_PSEAE Phosphate acetyltransferase OS=Pseudomonas aeruginosa (strain ATCC 15692 / PAO1 / 1C / PRS 101 / LMG 12228) GN=pta PE=1 SV=1

MHTFFIAPTGFGVGLTSISLGLLRALERAGLKVGFFKPIAQLHPGDLGPERSSELVARTH

GLDTPKPLPLAQVERMLGDGQLDELLEEIISLYQRAAADKDVVIVEGMVPTRHASYAARV

NFHLAKSLDAEVILVSAPENETLTELTDRIEIQAQLFGGPRDPKVLGVILNKVRGEADAA

NAEDGVADFARRLTEHSPLLRDDFRLIGCIPWQDELNAARTRDIADLLSARVINAGDYEQ

RRVQKIVLCARAVPNTVQLLKPGVLVVTPGDRDDIILAASLAAMNGVPLAGLLLCSDFPP

DPRIMELCRGALQGGLPVLSVATGSYDTATNLNRMNKEIPVDDRERAERVTEFVAGHIDF

EWLKQRCGTPRELRLSPPAFRYQVVQRAQKAGKRIVLPEGSEPRTVQAAAICQARGIARC

VLLAKPEEVQAVAQAQGIVLPEGLEIIDPDLVRQRYVEPMVELRKGKGLNAPMAEQQLED

SVVLATMMLALDEVDGLVSGAIHTTASTIRPALQLIKTAPGYNLVSSVFFMLLPDQVLVY

GDCAVNPDPSASDLAEIAVQSAASAQAFGIPARVAMISYSTGDSGSGVDVDKVREATRLA

REQRPDLLIDGPLQYDAAAIASVGRQKAPNSPVAGQATVFIFPDLNTGNTTYKAVQRSAD

CVSVGPMLQGLRKPVNDLSRGALVEDIVYTIALTAIQADAQAPA

>sp|P0A6A3|ACKA_ECOLI Acetate kinase OS=Escherichia coli (strain K12) GN=ackA PE=1 SV=1

MSSKLVLVLNCGSSSLKFAIIDAVNGEEYLSGLAECFHLPEARIKWKMDGNKQEAALGAG

AAHSEALNFIVNTILAQKPELSAQLTAIGHRIVHGGEKYTSSVVIDESVIQGIKDAASFA

PLHNPAHLIGIEEALKSFPQLKDKNVAVFDTAFHQTMPEESYLYALPYNLYKEHGIRRYG

AHGTSHFYVTQEAAKMLNKPVEELNIITCHLGNGGSVSAIRNGKCVDTSMGLTPLEGLVM

GTRSGDIDPAIIFHLHDTLGMSVDAINKLLTKESGLLGLTEVTSDCRYVEDNYATKEDAK

RAMDVYCHRLAKYIGAYTALMDGRLDAVVFTGGIGENAAMVRELSLGKLGVLGFEVDHER

NLAARFGKSGFINKEGTRPAVVIPTNEELVIAQDASRLTA

>sp|P38502|ACKA_METTE Acetate kinase OS=Methanosarcina thermophila GN=ackA PE=1 SV=1

MKVLVINAGSSSLKYQLIDMTNESALAVGLCERIGIDNSIITQKKFDGKKLEKLTDLPTH

KDALEEVVKALTDDEFGVIKDMGEINAVGHRVVHGGEKFTTSALYDEGVEKAIKDCFELA

PLHNPPNMMGISACAEIMPGTPMVIVFDTAFHQTMPPYAYMYALPYDLYEKHGVRKYGFH

GTSHKYVAERAALMLGKPAEETKIITCHLGNGSSITAVEGGKSVETSMGFTPLEGLAMGT

RCGSIDPAIVPFLMEKEGLTTREIDTLMNKKSGVLGVSGLSNDFRDLDEAASKGNRKAEL

ALEIFAYKVKKFIGEYSAVLNGADAVVFTAGIGENSASIRKRILTGLDGIGIKIDDEKNK

IRGQEIDISTPDAKVRVFVIPTNEELAIARETKEIVETEVKLRSSIPV

>sp|P37877|ACKA_BACSU Acetate kinase OS=Bacillus subtilis (strain 168) GN=ackA PE=1 SV=1

MSKIIAINAGSSSLKFQLFEMPSETVLTKGLVERIGIADSVFTISVNGEKNTEVTDIPDH

AVAVKMLLNKLTEFGIIKDLNEIDGIGHRVVHGGEKFSDSVLLTDETIKEIEDISELAPL

HNPANIVGIKAFKEVLPNVPAVAVFDTAFHQTMPEQSYLYSLPYEYYEKFGIRKYGFHGT

SHKYVTERAAELLGRPLKDLRLISCHLGNGASIAAVEGGKSIDTSMGFTPLAGVAMGTRS

GNIDPALIPYIMEKTGQTADEVLNTLNKKSGLLGISGFSSDLRDIVEATKEGNERAETAL

EVFASRIHKYIGSYAARMSGVDAIIFTAGIGENSVEVRERVLRGLEFMGVYWDPALNNVR

GEEAFISYPHSPVKVMIIPTDEEVMIARDVVRLAK

>sp|A0QQK1|ACKA_MYCS2 Acetate kinase OS=Mycobacterium smegmatis (strain ATCC 700084 / mc(2)155) GN=ackA PE=1 SV=1

MTVLVVNSGSSSLKYAVVRPASGEFLADGIIEEIGSGAVPDHDAALRAAFDELAAAGLHL

EDLDLKAVGHRMVHGGKTFYKPSVVDDELIAKARELSPLAPLHNPPAIKGIEVARKLLPD

LPHIAVFDTAFFHDLPAPASTYAIDRELAETWHIKRYGFHGTSHEYVSQQAAIFLDRPLE

SLNQIVLHLGNGASASAVAGGKAVDTSMGLTPMEGLVMGTRSGDIDPGVIMYLWRTAGMS

VDDIESMLNRRSGVLGLGGASDFRKLRELIESGDEHAKLAYDVYIHRLRKYIGAYMAVLG

RTDVISFTAGVGENVPPVRRDALAGLGGLGIEIDDALNSAKSDEPRLISTPDSRVTVLVV

PTNEELAIARACVGVV

>sp|P63411|ACKA_SALTY Acetate kinase OS=Salmonella typhimurium (strain LT2 / SGSC1412 / ATCC 700720) GN=ackA PE=1 SV=1

MSSKLVLVLNCGSSSLKFAIIDAVNGDEYLSGLAECFHLPEARIKWKMDGSKQEAALGAG

AAHSEALNFIVNTILAQKPELSAQLTAIGHRIVHGGEKYTSSVVIDESVIQGIKDSASFA

PLHNPAHLIGIAEALKSFPQLKDKNVAVFDTAFHQTMPEESYLYALPYSLYKEHGVRRYG

AHGTSHFYVTQEAAKMLNKPVEELNIITCHLGNGGSVSAIRNGKCVDTSMGLTPLEGLVM

GTRSGDIDPAIIFHLHDTLGMSVDQINKMLTKESGLLGLTEVTSDCRYVEDNYATKEDAK

RAMDVYCHRLAKYIGSYTALMDGRLDAVVFTGGIGENAAMVRELSLGKLGVLGFEVDHER

NLAARFGKSGFINKEGTRPAVVIPTNEELVIAQDASRLTA

>sp|Q9WYB1|ACKA_THEMA Acetate kinase OS=Thermotoga maritima (strain ATCC 43589 / MSB8 / DSM 3109 / JCM 10099) GN=ackA PE=1 SV=1

MRVLVINSGSSSIKYQLIEMEGEKVLCKGIAERIGIEGSRLVHRVGDEKHVIERELPDHE

EALKLILNTLVDEKLGVIKDLKEIDAVGHRVVHGGERFKESVLVDEEVLKAIEEVSPLAP

LHNPANLMGIKAAMKLLPGVPNVAVFDTAFHQTIPQKAYLYAIPYEYYEKYKIRRYGFHG

TSHRYVSKRAAEILGKKLEELKIITCHIGNGASVAAVKYGKCVDTSMGFTPLEGLVMGTR

SGDLDPAIPFFIMEKEGISPQEMYDILNKKSGVYGLSKGFSSDMRDIEEAALKGDEWCKL

VLEIYDYRIAKYIGAYAAAMNGVDAIVFTAGVGENSPITREDVCSYLEFLGVKLDKQKNE

ETIRGKEGIISTPDSRVKVLVVPTNEELMIARDTKEIVEKIGR

>sp|A0QLU8|ACKA_MYCA1 Acetate kinase OS=Mycobacterium avium (strain 104) GN=ackA PE=1 SV=1

MDGSDGARRVLVINSGSSSLKFQLVDPESGVAASTGIVERIGEESSPVPDHDAALRRAFD

MLAGDGVDLNTAGLVAVGHRVVHGGNTFYRPTVLDDAVIARLHELSELAPLHNPPALLGI

EVARRLLPGIAHVAVFDTGFFHDLPPAAATYAIDRELADRWQIRRYGFHGTSHRYVSEQA

AAFLDRPLRGLKQIVLHLGNGCSASAIAGTRPLDTSMGLTPLEGLVMGTRSGDIDPSVVS

YLCHTAGMGVDDVESMLNHRSGVVGLSGVRDFRRLRELIESGDGAAQLAYSVFTHRLRKY

IGAYLAVLGHTDVISFTAGIGENDAAVRRDAVSGMEELGIVLDERRNLPGAKGARQISAD

DSPITVLVVPTNEELAIARDCVRVLGG

>sp|B2HPZ3|ACKA_MYCMM Acetate kinase OS=Mycobacterium marinum (strain ATCC BAA-535 / M) GN=ackA PE=1 SV=1

MSASRPNRVVLVLNSGSSSLKFQLVEPDSGMSRATGNIERIGEESSSVPDHDAALRRVFE

ILAEDDIDLQSCGLVAVGHRVVHGGKDFYEPTLLNDAVIGKLDELSPLAPLHNPPAVLCI

RVARALLPDVPHIAVFDTAFFHQLPPAAATYAIDRELADVWKIRRYGFHGTSHEYVSQQA

AEFLGKPIGDLNQIVLHLGNGASASAVAGGRPVETSMGLTPLEGLVMGTRSGDLDPGVIG

YLWRTAKLGVDEIESMLNHRSGMLGLAGERDFRRLRAMIDDGDPAAELAYDVFIHRLRKY

VGAYLAVLGHTDVVSFTAGIGEHDAAVRRDTLAGMAELGISLDERRNACPSGGARRISAD

DSPVTVLVIPTNEELAIARHCCSVLVAV

>sp|Q73T33|ACKA_MYCPA Acetate kinase OS=Mycobacterium paratuberculosis (strain ATCC BAA-968 / K-10) GN=ackA PE=1 SV=1

MDGSDGARRVLVINSGSSSLKFQLVDPEFGVAASTGIVERIGEESSPVPDHDAALRRAFD

MLAGDGVDLNTAGLVAVGHRVVHGGNTFYRPTVLDDAVIARLHELSELAPLHNPPALQGI

EVARRLLPDIAHVAVFDTGFFHDLPPAAATYAIDRELADRWQIRRYGFHGTSHRYVSEQA

AAFLDRPLRGLKQIVLHLGNGCSASAIAGTRPLDTSMGLTPLEGLVMGTRSGDIDPSIVS

YLCHTAGMGVDDVESMLNHRSGVVGLSGVRDFRRLRELIESGDGAAQLAYSVFTHRLRKY

IGAYLAVLGHTDVISFTAGIGENDAAVRRDAVSGMEELGIVLDERRNLAGGKGARQISAD

DSPITVLVVPTNEELAIARDCVRVLGG

>sp|P75245|ACKA_MYCPN Acetate kinase OS=Mycoplasma pneumoniae (strain ATCC 29342 / M129) GN=ackA PE=1 SV=1

MNDNKILVVNAGSSSIKFQLFDYHKKVLAKALCERIFVDGFFKLEFNEQKVEEKVAFPDH

HAAVTHFLNTLKKHKIIQELSDIILVGHRVVQGANYFKDSVIVDAEALAKIKEFIKLAPL

HNKPEADVIEIFFKEVPSAKNVAVFDTTFHTTIPQENYLYAVPRSWEQKHLVRRYGFHGT

SYKFINNYLEKHLNKQNLNLIVCHLGNGASVCAIKNGKSFNTSMGFTPLEGLIMGTRSGD

LDPAIIGYVAEQENMSASDVVNALNKKSGMLALTGASDMRDVFAKPQENAVAIKMYVNRV

ADYIAKYLNQLEGNIDGLVFTGGIGENASDCVELFINAVKSLGFATDLKLFVKYGDSCVV

STPQSKYKIYRVRTNEELMIVEDSIRLTQK

>sp|P9WQH1|ACKA_MYCTU Acetate kinase OS=Mycobacterium tuberculosis (strain ATCC 25618 / H37Rv) GN=ackA PE=1 SV=1

MSSTVLVINSGSSSLKFQLVEPVAGMSRAAGIVERIGERSSPVADHAQALHRAFKMLAED

GIDLQTCGLVAVGHRVVHGGTEFHQPTLLDDTVIGKLEELSALAPLHNPPAVLGIKVARR

LLANVAHVAVFDTAFFHDLPPAAATYAIDRDVADRWHIRRYGFHGTSHQYVSERAAAFLG

RPLDGLNQIVLHLGNGASASAIARGRPVETSMGLTPLEGLVMGTRSGDLDPGVISYLWRT

ARMGVEDIESMLNHRSGMLGLAGERDFRRLRLVIETGDRSAQLAYEVFIHRLRKYLGAYL

AVLGHTDVVSFTAGIGENDAAVRRDALAGLQGLGIALDQDRNLGPGHGARRISSDDSPIA

VLVVPTNEELAIARDCLRVLGGRRA

>sp|Q99TF2|ACKA_STAAN Acetate kinase OS=Staphylococcus aureus (strain N315) GN=ackA PE=1 SV=1

MSKLILAINAGSSSLKFQLIRMPEEELVTKGLIERIGLKDSIFTIEVNGEKVKTVQDIKD

HVEAVDIMLDAFKAHNIINDINDIDGTGHRVVHGGEKFPESVAITDEVEKEIEELSELAP

LHNPANLMGIRAFRKLLPNIPHVAIFDTAFHQTMPEKAYLYSLPYHYYKDYGIRKYGFHG

TSHKFVSQRAAEMLDKPIEDLRIISCHIGNGASIAAIDGGKSIDTSMGFTPLAGVTMGTR

SGNIDPALIPFIMEKTGKTAEQVLEILNKESGLLGLSGTSSDLRDLSEEAESGKARSQMA

LDVFASKIHKYIGSYAARMHGVDVIVFTAGIGENSVEIRAKVLEGLEFMGVYWDPKKNEN

LLRGKEGFINYPHSPVKVVVIPTDEESMIARDVMTFGGLK

*2. Acetate formation pathway II.*

>sp|P08559|ODPA_HUMAN Pyruvate dehydrogenase E1 component subunit alpha, somatic form, mitochondrial OS=Homo sapiens GN=PDHA1 PE=1 SV=3

MRKMLAAVSRVLSGASQKPASRVLVASRNFANDATFEIKKCDLHRLEEGPPVTTVLTRED

GLKYYRMMQTVRRMELKADQLYKQKIIRGFCHLCDGQEACCVGLEAGINPTDHLITAYRA

HGFTFTRGLSVREILAELTGRKGGCAKGKGGSMHMYAKNFYGGNGIVGAQVPLGAGIALA

CKYNGKDEVCLTLYGDGAANQGQIFEAYNMAALWKLPCIFICENNRYGMGTSVERAAAST

DYYKRGDFIPGLRVDGMDILCVREATRFAAAYCRSGKGPILMELQTYRYHGHSMSDPGVS

YRTREEIQEVRSKSDPIMLLKDRMVNSNLASVEELKEIDVEVRKEIEDAAQFATADPEPP

LEELGYHIYSSDPPFEVRGANQWIKFKSVS

>sp|P35486|ODPA_MOUSE Pyruvate dehydrogenase E1 component subunit alpha, somatic form, mitochondrial OS=Mus musculus GN=Pdha1 PE=1 SV=1

MRKMLAAVSRVLAGSAQKPASRVLVASRNFANDATFEIKKCDLHRLEEGPPVTTVLTRED

GLKYYRMMQTVRRMELKADQLYKQKIIRGFCHLCDGQEACCVGLEAGINPTDHLITAYRA

HGFTFTRGLPVRAILAELTGRRGGCAKGKGGSMHMYAKNFYGGNGIVGAQVPLGAGIALA

CKYNGKDEVCLTLYGDGAANQGQIFEAYNMAALWKLPCIFICENNRYGMGTSVERAAAST

DYYKRGDFIPGLRVDGMDILCVREATKFAAAYCRSGKGPILMELQTYRYHGHSMSDPGVS

YRTREEIQEVRSKSDPIMLLKDRMVNSNLASVEELKEIDVEVRKEIEDAAQFATADPEPP

LEELGYHIYSSDPPFEVRGANQWIKFKSVS

>sp|P11177|ODPB_HUMAN Pyruvate dehydrogenase E1 component subunit beta, mitochondrial OS=Homo sapiens GN=PDHB PE=1 SV=3

MAAVSGLVRRPLREVSGLLKRRFHWTAPAALQVTVRDAINQGMDEELERDEKVFLLGEEV

AQYDGAYKVSRGLWKKYGDKRIIDTPISEMGFAGIAVGAAMAGLRPICEFMTFNFSMQAI

DQVINSAAKTYYMSGGLQPVPIVFRGPNGASAGVAAQHSQCFAAWYGHCPGLKVVSPWNS

EDAKGLIKSAIRDNNPVVVLENELMYGVPFEFPPEAQSKDFLIPIGKAKIERQGTHITVV

SHSRPVGHCLEAAAVLSKEGVECEVINMRTIRPMDMETIEASVMKTNHLVTVEGGWPQFG

VGAEICARIMEGPAFNFLDAPAVRVTGADVPMPYAKILEDNSIPQVKDIIFAIKKTLNI

>sp|P0AFG8|ODP1_ECOLI Pyruvate dehydrogenase E1 component OS=Escherichia coli (strain K12) GN=aceE PE=1 SV=2

MSERFPNDVDPIETRDWLQAIESVIREEGVERAQYLIDQLLAEARKGGVNVAAGTGISNY

INTIPVEEQPEYPGNLELERRIRSAIRWNAIMTVLRASKKDLELGGHMASFQSSATIYDV

CFNHFFRARNEQDGGDLVYFQGHISPGVYARAFLEGRLTQEQLDNFRQEVHGNGLSSYPH

PKLMPEFWQFPTVSMGLGPIGAIYQAKFLKYLEHRGLKDTSKQTVYAFLGDGEMDEPESK

GAITIATREKLDNLVFVINCNLQRLDGPVTGNGKIINELEGIFEGAGWNVIKVMWGSRWD

ELLRKDTSGKLIQLMNETVDGDYQTFKSKDGAYVREHFFGKYPETAALVADWTDEQIWAL

NRGGHDPKKIYAAFKKAQETKGKATVILAHTIKGYGMGDAAEGKNIAHQVKKMNMDGVRH

IRDRFNVPVSDADIEKLPYITFPEGSEEHTYLHAQRQKLHGYLPSRQPNFTEKLELPSLQ

DFGALLEEQSKEISTTIAFVRALNVMLKNKSIKDRLVPIIADEARTFGMEGLFRQIGIYS

PNGQQYTPQDREQVAYYKEDEKGQILQEGINELGAGCSWLAAATSYSTNNLPMIPFYIYY

SMFGFQRIGDLCWAAGDQQARGFLIGGTSGRTTLNGEGLQHEDGHSHIQSLTIPNCISYD

PAYAYEVAVIMHDGLERMYGEKQENVYYYITTLNENYHMPAMPEGAEEGIRKGIYKLETI

EGSKGKVQLLGSGSILRHVREAAEILAKDYGVGSDVYSVTSFTELARDGQDCERWNMLHP

LETPRVPYIAQVMNDAPAVASTDYMKLFAEQVRTYVPADDYRVLGTDGFGRSDSRENLRH

HFEVDASYVVVAALGELAKRGEIDKKVVADAIAKFNIDADKVNPRLA

>sp|P29803|ODPAT_HUMAN Pyruvate dehydrogenase E1 component subunit alpha, testis-specific form, mitochondrial OS=Homo sapiens GN=PDHA2 PE=1 SV=1

MLAAFISRVLRRVAQKSARRVLVASRNSSNDATFEIKKCDLYLLEEGPPVTTVLTRAEGL

KYYRMMLTVRRMELKADQLYKQKFIRGFCHLCDGQEACCVGLEAGINPSDHVITSYRAHG

VCYTRGLSVRSILAELTGRRGGCAKGKGGSMHMYTKNFYGGNGIVGAQGPLGAGIALACK

YKGNDEICLTLYGDGAANQGQIAEAFNMAALWKLPCVFICENNLYGMGTSTERAAASPDY

YKRGNFIPGLKVDGMDVLCVREATKFAANYCRSGKGPILMELQTYRYHGHSMSDPGVSYR

TREEIQEVRSKRDPIIILQDRMVNSKLATVEELKEIGAEVRKEIDDAAQFATTDPEPHLE

ELGHHIYSSDSSFEVRGANPWIKFKSVS

>sp|P16387|ODPA_YEAST Pyruvate dehydrogenase E1 component subunit alpha, mitochondrial OS=Saccharomyces cerevisiae (strain ATCC 204508 / S288c) GN=PDA1 PE=1 SV=2

MLAASFKRQPSQLVRGLGAVLRTPTRIGHVRTMATLKTTDKKAPEDIEGSDTVQIELPES

SFESYMLEPPDLSYETSKATLLQMYKDMVIIRRMEMACDALYKAKKIRGFCHLSVGQEAI

AVGIENAITKLDSIITSYRCHGFTFMRGASVKAVLAELMGRRAGVSYGKGGSMHLYAPGF

YGGNGIVGAQVPLGAGLAFAHQYKNEDACSFTLYGDGASNQGQVFESFNMAKLWNLPVVF

CCENNKYGMGTAASRSSAMTEYFKRGQYIPGLKVNGMDILAVYQASKFAKDWCLSGKGPL

VLEYETYRYGGHSMSDPGTTYRTRDEIQHMRSKNDPIAGLKMHLIDLGIATEAEVKAYDK

SARKYVDEQVELADAAPPPEAKLSILFEDVYVKGTETPTLRGRIPEDTWDFKKQGFASRD

>sp|Q8H1Y0|ODPA2_ARATH Pyruvate dehydrogenase E1 component subunit alpha-2, mitochondrial OS=Arabidopsis thaliana GN=IAR4 PE=1 SV=2

MALSRLSSRSNTFLKPAITALPSSIRRHVSTDSSPITIETAVPFTSHLCESPSRSVETSS

EEILAFFRDMARMRRMEIAADSLYKAKLIRGFCHLYDGQEALAVGMEAAITKKDAIITSY

RDHCTFIGRGGKLVDAFSELMGRKTGCSHGKGGSMHFYKKDASFYGGHGIVGAQIPLGCG

LAFAQKYNKDEAVTFALYGDGAANQGQLFEALNISALWDLPAILVCENNHYGMGTATWRS

AKSPAYFKRGDYVPGLKVDGMDALAVKQACKFAKEHALKNGPIILEMDTYRYHGHSMSDP

GSTYRTRDEISGVRQVRDPIERVRKLLLTHDIATEKELKDMEKEIRKEVDDAVAQAKESP

IPDASELFTNMYVKDCGVESFGADRKELKVTLP

>sp|P35487|ODPAT_MOUSE Pyruvate dehydrogenase E1 component subunit alpha, testis-specific form, mitochondrial OS=Mus musculus GN=Pdha2 PE=1 SV=1

MRKMLTAVLSHVFSGMVQKPALRGLLSSLKFSNDATCDIKKCDLYRLEEGPPTSTVLTRA

EALKYYRTMQVIRRMELKADQLYKQKFIRGFCHLCDGQEACCVGLEAGINPTDHVITSYR

AHGFCYTRGLSVKSILAELTGRKGGCAKGKGGSMHMYGKNFYGGNGIVGAQVPLGAGVAF

ACKYLKNGQVCLALYGDGAANQGQVFEAYNMSALWKLPCVFICENNLYGMGTSNERSAAS

TDYHKKGFIIPGLRVNGMDILCVREATKFAADHCRSGKGPIVMELQTYRYHGHSMSDPGI

SYRSREEVHNVRSKSDPIMLLRERIISNNLSNIEELKEIDADVKKEVEDAAQFATTDPEP

AVEDIANYLYHQDPPFEVRGAHKWLKYKSHS

>sp|P26284|ODPA_RAT Pyruvate dehydrogenase E1 component subunit alpha, somatic form, mitochondrial OS=Rattus norvegicus GN=Pdha1 PE=1 SV=2

MRKMLAAVSRVLAGAAQKPASRVLVASRNFANDATFEIKKCDLHRLEEGPPVTTVLTRED

GLKYYRMMQTVRRMELKADQLYKQKIIRGFCHLCDGQEACCVGLEAGINPTDHLITAYRA

HGFTFNRGHAVRAILAELTGRRGGCAKGKGGSMHMYAKNFYGGNGIVGAQVPLGAGIALA

CKYNGKDEVCLTLYGDGAANQGQIFEAYNMAALWKLPCIFICENNRYGMGTSVERAAAST

DYYKRGDFIPGLRVDGMDILCVREATKFAAAYCRSGKGPILMELQTYRYHGHSMSDPGVS

YRTREEIQEVRSKSDPIMLLKDRMVNSNLASVEELKEIDVEVRKEIEDAAQFATADPEPP

LEELGYHIYSSDPPFEVRGANQWIKFKSVS

>sp|Q38799|ODPB1_ARATH Pyruvate dehydrogenase E1 component subunit beta-1, mitochondrial OS=Arabidopsis thaliana GN=PDH2 PE=1 SV=2

MLGILRQRAIDGASTLRRTRFALVSARSYAAGAKEMTVRDALNSAIDEEMSADPKVFVMG

EEVGQYQGAYKITKGLLEKYGPERVYDTPITEAGFTGIGVGAAYAGLKPVVEFMTFNFSM

QAIDHIINSAAKSNYMSAGQINVPIVFRGPNGAAAGVGAQHSQCYAAWYASVPGLKVLAP

YSAEDARGLLKAAIRDPDPVVFLENELLYGESFPISEEALDSSFCLPIGKAKIEREGKDV

TIVTFSKMVGFALKAAEKLAEEGISAEVINLRSIRPLDRATINASVRKTSRLVTVEEGFP

QHGVCAEICASVVEESFSYLDAPVERIAGADVPMPYAANLERLALPQIEDIVRASKRACY

RSK

>sp|Q9D051|ODPB_MOUSE Pyruvate dehydrogenase E1 component subunit beta, mitochondrial OS=Mus musculus GN=Pdhb PE=1 SV=1

MAVVAGLVRGPLRQASGLLKRRFHRSAPAAVQLTVREAINQGMDEELERDEKVFLLGEEV

AQYDGAYKVSRGLWKKYGDKRIIDTPISEMGFAGIAVGAAMAGLRPICEFMTFNFSMQAI

DQVINSAAKTYYMSAGLQPVPIVFRGPNGASAGVAAQHSQCFAAWYGHCPGLKVVSPWNS

EDAKGLIKSAIRDNNPVVMLENELMYGVAFELPAEAQSKDFLIPIGKAKIERQGTHITVV

AHSRPVGHCLEAAAVLSKEGIECEVINLRTIRPMDIEAIEASVMKTNHLVTVEGGWPQFG

VGAEICARIMEGPAFNFLDAPAVRVTGADVPMPYAKVLEDNSVPQVKDIIFAVKKTLNI

>sp|P32473|ODPB_YEAST Pyruvate dehydrogenase E1 component subunit beta, mitochondrial OS=Saccharomyces cerevisiae (strain ATCC 204508 / S288c) GN=PDB1 PE=1 SV=2

MFSRLPTSLARNVARRAPTSFVRPSAAAAALRFSSTKTMTVREALNSAMAEELDRDDDVF

LIGEEVAQYNGAYKVSKGLLDRFGERRVVDTPITEYGFTGLAVGAALKGLKPIVEFMSFN

FSMQAIDHVVNSAAKTHYMSGGTQKCQMVFRGPNGAAVGVGAQHSQDFSPWYGSIPGLKV

LVPYSAEDARGLLKAAIRDPNPVVFLENELLYGESFEISEEALSPEFTLPYKAKIEREGT

DISIVTYTRNVQFSLEAAEILQKKYGVSAEVINLRSIRPLDTEAIIKTVKKTNHLITVES

TFPSFGVGAEIVAQVMESEAFDYLDAPIQRVTGADVPTPYAKELEDFAFPDTPTIVKAVK

EVLSIE

>sp|Q8NNF6|ODP1_CORGL Pyruvate dehydrogenase E1 component OS=Corynebacterium glutamicum (strain ATCC 13032 / DSM 20300 / JCM 1318 / LMG 3730 / NCIMB 10025) GN=aceE PE=1 SV=1

MADQAKLGGKPSDDSNFAMIRDGVASYLNDSDPEETNEWMDSLDGLLQESSPERARYLML

RLLERASAKRVSLPPMTSTDYVNTIPTSMEPEFPGDEEMEKRYRRWIRWNAAIMVHRAQR

PGIGVGGHISTYAGAAPLYEVGFNHFFRGKDHPGGGDQIFFQGHASPGMYARAFMEGRLS

EDDLDGFRQEVSREQGGIPSYPHPHGMKDFWEFPTVSMGLGPMDAIYQARFNRYLENRGI

KDTSDQHVWAFLGDGEMDEPESRGLIQQAALNNLDNLTFVVNCNLQRLDGPVRGNTKIIQ

ELESFFRGAGWSVIKVVWGREWDELLEKDQDGALVEIMNNTSDGDYQTFKANDGAYVREH

FFGRDPRTAKLVENMTDEEIWKLPRGGHDYRKVYAAYKRALETKDRPTVILAHTIKGYGL

GHNFEGRNATHQMKKLTLDDLKLFRDKQGIPITDEQLEKDPYLPPYYHPGEDAPEIKYMK

ERRAALGGYLPERRENYDPIQVPPLDKLRSVRKGSGKQQIATTMATVRTFKELMRDKGLA

DRLVPIIPDEARTFGLDSWFPTLKIYNPHGQNYVPVDHDLMLSYREAPEGQILHEGINEA

GSVASFIAAGTSYATHGKAMIPLYIFYSMFGFQRTGDSIWAAADQMARGFLLGATAGRTT

LTGEGLQHMDGHSPVLASTNEGVETYDPSFAYEIAHLVHRGIDRMYGPGKGEDVIYYITI

YNEPTPQPAEPEGLDVEGLHKGIYLYSRGEGTGHEANILASGVGMQWALKAASILEADYG

VRANIYSATSWVNLARDGAARNKAQLRNPGADAGEAFVTTQLKQTSGPYVAVSDFSTDLP

NQIREWVPGDYTVLGADGFGFSDTRPAARRFFNIDAESIVVAVLNSLAREGKIDVSVAAQ

AAEKFKLDDPTSVSVDPNAPEE

>sp|P0AFG9|ODP1_ECO57 Pyruvate dehydrogenase E1 component OS=Escherichia coli O157:H7 GN=aceE PE=1 SV=2

MSERFPNDVDPIETRDWLQAIESVIREEGVERAQYLIDQLLAEARKGGVNVAAGTGISNY

INTIPVEEQPEYPGNLELERRIRSAIRWNAIMTVLRASKKDLELGGHMASFQSSATIYDV

CFNHFFRARNEQDGGDLVYFQGHISPGVYARAFLEGRLTQEQLDNFRQEVHGNGLSSYPH

PKLMPEFWQFPTVSMGLGPIGAIYQAKFLKYLEHRGLKDTSKQTVYAFLGDGEMDEPESK

GAITIATREKLDNLVFVINCNLQRLDGPVTGNGKIINELEGIFEGAGWNVIKVMWGSRWD

ELLRKDTSGKLIQLMNETVDGDYQTFKSKDGAYVREHFFGKYPETAALVADWTDEQIWAL

NRGGHDPKKIYAAFKKAQETKGKATVILAHTIKGYGMGDAAEGKNIAHQVKKMNMDGVRH

IRDRFNVPVSDADIEKLPYITFPEGSEEHTYLHAQRQKLHGYLPSRQPNFTEKLELPSLQ

DFGALLEEQSKEISTTIAFVRALNVMLKNKSIKDRLVPIIADEARTFGMEGLFRQIGIYS

PNGQQYTPQDREQVAYYKEDEKGQILQEGINELGAGCSWLAAATSYSTNNLPMIPFYIYY

SMFGFQRIGDLCWAAGDQQARGFLIGGTSGRTTLNGEGLQHEDGHSHIQSLTIPNCISYD

PAYAYEVAVIMHDGLERMYGEKQENVYYYITTLNENYHMPAMPEGAEEGIRKGIYKLETI

EGSKGKVQLLGSGSILRHVREAAEILAKDYGVGSDVYSVTSFTELARDGQDCERWNMLHP

LETPRVPYIAQVMNDAPAVASTDYMKLFAEQVRTYVPADDYRVLGTDGFGRSDSRENLRH

HFEVDASYVVVAALGELAKRGEIDKKVVADAIAKFNIDADKVNPRLA

>sp|P9WIS9|ODP1_MYCTU Pyruvate dehydrogenase E1 component OS=Mycobacterium tuberculosis (strain ATCC 25618 / H37Rv) GN=aceE PE=1 SV=2

MTTDFARHDLAQNSNSASEPDRVRVIREGVASYLPDIDPEETSEWLESFDTLLQRCGPSR

ARYLMLRLLERAGEQRVAIPALTSTDYVNTIPTELEPWFPGDEDVERRYRAWIRWNAAIM

VHRAQRPGVGVGGHISTYASSAALYEVGFNHFFRGKSHPGGGDQVFIQGHASPGIYARAF

LEGRLTAEQLDGFRQEHSHVGGGLPSYPHPRLMPDFWEFPTVSMGLGPLNAIYQARFNHY

LHDRGIKDTSDQHVWCFLGDGEMDEPESRGLAHVGALEGLDNLTFVINCNLQRLDGPVRG

NGKIIQELESFFRGAGWNVIKVVWGREWDALLHADRDGALVNLMNTTPDGDYQTYKANDG

GYVRDHFFGRDPRTKALVENMSDQDIWNLKRGGHDYRKVYAAYRAAVDHKGQPTVILAKT

IKGYALGKHFEGRNATHQMKKLTLEDLKEFRDTQRIPVSDAQLEENPYLPPYYHPGLNAP

EIRYMLDRRRALGGFVPERRTKSKALTLPGRDIYAPLKKGSGHQEVATTMATVRTFKEVL

RDKQIGPRIVPIIPDEARTFGMDSWFPSLKIYNRNGQLYTAVDADLMLAYKESEVGQILH

EGINEAGSVGSFIAAGTSYATHNEPMIPIYIFYSMFGFQRTGDSFWAAADQMARGFVLGA

TAGRTTLTGEGLQHADGHSLLLAATNPAVVAYDPAFAYEIAYIVESGLARMCGENPENIF

FYITVYNEPYVQPPEPENFDPEGVLRGIYRYHAATEQRTNKAQILASGVAMPAALRAAQM

LAAEWDVAADVWSVTSWGELNRDGVAIETEKLRHPDRPAGVPYVTRALENARGPVIAVSD

WMRAVPEQIRPWVPGTYLTLGTDGFGFSDTRPAARRYFNTDAESQVVAVLEALAGDGEID

PSVPVAAARQYRIDDVAAAPEQTTDPGPGA

>sp|Q06437|ODPAT_RAT Pyruvate dehydrogenase E1 component subunit alpha, testis-specific form, mitochondrial OS=Rattus norvegicus GN=Pdha2 PE=1 SV=1

MRKMLATVLSQVFSGMVQKPALRGLLSSLKFSNDATCDIKKCDLYLLEQGPPTSTVLTRE

EALKYYRNMQVIRRMELKADQLYKQKFIRGFCHLCDGQEACNVGLEAGINPTDHIITSYR

AHGLCYTRGLSVKSILAELTGRKGGCAKGKGGSMHMYAKNFYGGNGIVGAQVPLGAGVAL

ACKYLKNGQICLALYGDGAANQGQVFEAYNMSALWKLPCVFICENNRYGMGTAIERSAAS

TDYHKKGFVIPGLRVNGMDILSVREATKFAADHCRSGKGPIVMELQTYRYHGHSMSDPGI

SYRTREEVQNVRSKSDPIMLLRERMISNNLSSVEELKEIDADVKKEVEEAAQFATTDPEP

PLEDLANYLYHQNPPFEVRGAHKWLKFKSVS

>sp|P21881|ODPA_BACSU Pyruvate dehydrogenase E1 component subunit alpha OS=Bacillus subtilis (strain 168) GN=pdhA PE=1 SV=3

MAAKTKKAIVDSKKQFDAIKKQFETFQILNEKGEVVNEAAMPDLTDDQLKELMRRMVFTR

VLDQRSISLNRQGRLGFYAPTAGQEASQIATHFALEKEDFVLPGYRDVPQLIWHGLPLYQ

AFLFSRGHFRGNQMPDDVNALSPQIIIGAQYIQTAGVALGLKKRGKKAVAITYTGDGGAS

QGDFYEGINFAGAYKAPAIFVVQNNRYAISTPVEKQSAAETIAQKAVAAGIVGVQVDGMD

PLAVYAATAEARERAINGEGPTLIETLTFRYGPHTMAGDDPTKYRTKEIENEWEQKDPLV

RFRAFLENKGLWSEEEEAKVIEDAKEEIKQAIKKADAEPKQKVTDLMKIMYEKMPHNLEE

QFEIYTQKESK

>sp|P21874|ODPB_GEOSE Pyruvate dehydrogenase E1 component subunit beta OS=Geobacillus stearothermophilus GN=pdhB PE=1 SV=2

MAQMTMVQAITDALRIELKNDPNVLIFGEDVGVNGGVFRATEGLQAEFGEDRVFDTPLAE

SGIGGLAIGLALQGFRPVPEIQFFGFVYEVMDSICGQMARIRYRTGGRYHMPITIRSPFG

GGVHTPELHSDSLEGLVAQQPGLKVVIPSTPYDAKGLLISAIRDNDPVIFLEHLKLYRSF

RQEVPEGEYTIPIGKADIKREGKDITIIAYGAMVHESLKAAAELEKEGISAEVVDLRTVQ

PLDIETIIGSVEKTGRAIVVQEAQRQAGIAANVVAEINERAILSLEAPVLRVAAPDTVYP

FAQAESVWLPNFKDVIETAKKVMNF

>sp|P49432|ODPB_RAT Pyruvate dehydrogenase E1 component subunit beta, mitochondrial OS=Rattus norvegicus GN=Pdhb PE=1 SV=2

MAAVAGLVRGPLRQASGLLKRRFHRSAPAAVQLTVREAINQGMDEELERDEKVFLLGEEV

AQYDGAYKVSRGLWKKYGDKRIIDTPISEMGFAGIAVGAAMAGLRPICEFMTFNFSMQAI

DQVINSAAKTYYMSAGLQPVPIVFRGPNGASAGVAAQHSQCFAAWYGHCPGLKVVSPWNS

EDAKGLIKSAIRDDNPVVMLENELMYGVAFELPTEAQSKDFLIPIGKAKIERQGTHITVV

AHSRPVGHCLEAAAVLSKEGIECEVINLRTIRPMDIEAIEASVMKTNHLVTVEGGWPQFG

VGAEICARIMEGPAFNFLDAPAVRVTGADVPMPYAKILEDNSIPQVKDIIFAIKKTLNI

>sp|A0R0B0|ODP1_MYCS2 Pyruvate dehydrogenase E1 component OS=Mycobacterium smegmatis (strain ATCC 700084 / mc(2)155) GN=aceE PE=1 SV=1

MTTEFVRQDLAQNSSTAAEPDRVRVIREGVASYLPDIDTEETAEWLESFDELLERSGPAR

ARYLMLRLLERAGEQRVAIPALTSTDYVNTIPTELEPWFPGDEDVERRYRAWIRWNAAIM

VHRAQRPGVGVGGHISTYASSATLYEVGFNHFFRGKSHPGGGDHVFIQGHASPGIYARAF

LEGRLTTDQLDGFRQEHSHSGGGLPSYPHPRLMPDFWEFPTVSMGLGPMNAIYQARFNHY

LHDRGIKDTSDQHVWAFLGDGEMDEPESRGLIQVAANEALDNLTFVINCNLQRLDGPVRG

NGKIIQELESFFRGAGWNVIKVVWGREWDVLLHADRDGALVNLMNSTPDGDYQTYKANDG

AYVRDHFFGRDPRTKALVADMSDQEIWNLKRGGHDYRKVYAAYRAAMEHKGQPTVILAKT

IKGYTLGQHFEGRNATHQMKKLALEDLKNFRDVTRVPVSDAQLEEDPYLPPYYHPGPEAP

EIRYLLERRRALGGFVPSRRTKSKPLALPGSDTYKALKKGSGSQAVATTMATVRTFKELL

RDKNIGPRIVPIIPDEARTFGMDSWFPSLKIYNRNGQLYTSVDSELMLAYKESEVGQILH

EGINEAGSTSSFTAVGTSYSTHDEPMIPIYIFYSMFGFQRTGDGLWAAADQMARGFVLGA

TAGRTTLTGEGLQHADGHSLLLASTNPAAVTYDPAFAYEIAHIIESGLQRMYGEDPENVF

FYLTIYNEPYQQPAEPENLDVEALLKGLYLYRPAPEKRAKSAQILASGVAMPEALRAADL

LASDWDVAADVWSVTSWGELNREGVAIEKHRLRHPDEPAGTPHVTSALADAAGPVIAVSD

WMRAVPEQIRPWVPGTYVTLGTDGFGFSDTRPAARRYFNTDAESVVVAVLQGLARDGEID

ASVAAQAAEQYRIDDVSAAGVSYADTGSA

>sp|Q4MTG0|ODPA_BACCE Pyruvate dehydrogenase E1 component subunit alpha OS=Bacillus cereus GN=pdhA PE=1 SV=3

MGTKTKKTLFNVDEQMKAIAAQFETLQILNEKGEVVNEAAMPELSDDQLKELMRRMVYTR

VLDQRSISLNRQGRLGFYAPTAGQEASQLASHFALEAEDFILPGYRDVPQLVWHGLPLYQ

AFLFSRGHFMGNQMPENVNALAPQIIIGAQIIQTAGVALGMKLRGKKSVAITYTGDGGAS

QGDFYEGMNFAGAFKAPAIFVVQNNRYAISTPVEKQSAAKTVAQKAVAAGIYGIQVDGMD

PLAVYAATAFARERAVNGEGPTLIETLTFRYGPHTMAGDDPTRYRTKDIENEWEQKDPIV

RFRAFLENKGLWSQEVEEKVIEEAKEDIKQAIAKADQAPKQKVTDLMEIMYEKMPYNLAE

QYEIYKEKESK

>sp|P26267|ODPA_ASCSU Pyruvate dehydrogenase E1 component subunit alpha type I, mitochondrial OS=Ascaris suum PE=1 SV=1

MIFVFANIFKVPTVSPSVMAISVRLASTEATFQTKPFKLHKLDSGPDINVHVTKEDAVHY

YTQMLTIRRMESAAGNLYKEKKVRGFCHLYSGQEACAVGTKAAMDAGDAAVTAYRCHGWT

YLSGSSVAKVLCELTGRITGNVYGKGGSMHMYGENFYGGNGIVGAQQPLGTGIAFAMKYR

KEKNVCITMFGDGATNQGQLFESMNMAKLWDLPVLYVCENNGYGMGTAAARSSASTDYYT

RGDYVPGIWVDGMDVLAVRQAVRWAKEWCNAGKGPLMIEMATYRYSGHSMSDPGTSYRTR

EEVQEVRKTRDPITGFKDKIVTAGLVTEDEIKEIDKQVRKEIDAAVKQAHTDKESPVELM

LTDIYYNTPAQYVRCTTDEVLQKYLTSEEAVKALAK

>sp|P21873|ODPA_GEOSE Pyruvate dehydrogenase E1 component subunit alpha OS=Geobacillus stearothermophilus GN=pdhA PE=1 SV=2

MGVKTFQFPFAEQLEKVAEQFPTFQILNEEGEVVNEEAMPELSDEQLKELMRRMVYTRIL

DQRSISLNRQGRLGFYAPTAGQEASQIASHFALEKEDFILPGYRDVPQIIWHGLPLYQAF

LFSRGHFHGNQIPEGVNVLPPQIIIGAQYIQAAGVALGLKMRGKKAVAITYTGDGGTSQG

DFYEGINFAGAFKAPAIFVVQNNRFAISTPVEKQTVAKTLAQKAVAAGIPGIQVDGMDPL

AVYAAVKAARERAINGEGPTLIETLCFRYGPHTMSGDDPTRYRSKELENEWAKKDPLVRF

RKFLEAKGLWSEEEENNVIEQAKEEIKEAIKKADETPKQKVTDLISIMFEELPFNLKEQY

EIYKEKESK

>sp|Q54C70|ODPA_DICDI Pyruvate dehydrogenase E1 component subunit alpha, mitochondrial OS=Dictyostelium discoideum GN=pdhA PE=1 SV=1

MLSNFLKVNSKALGHIRTFASKSGEIKHNFKKADTYLCDGPSDSTVTNKDELISFFTEMS

RFRRLETVCDGLYKKKLIRGFCHLYTGQEAVCAGLESAITKDDHIITAYRDHTYMLSRGA

TPEEIFAELLMKETGCSKGKGGSMHMFTKNFYGGNGIVGAQCPLGAGIAFAQKYNKTGNV

CLAMYGDGAANQGQLFEAFNMASLWKLPVIFICENNKYGMGTSQKRSTAGHDFYTRGHYV

AGLKVDGMDVFAVKEAGKYAAEWCRAGNGPIILEMDTYRYVGHSMSDPGITYRTREEVNH

VRQTRDPIENIRQIILDNKIATEDQLAAIEETVRDEMEKASEKAIAAPLPQARELFTNVY

LQEVPVRGVEFVNSFKP

>sp|P29804|ODPA_PIG Pyruvate dehydrogenase E1 component subunit alpha, somatic form, mitochondrial (Fragment) OS=Sus scrofa GN=PDHA1 PE=1 SV=1

GKMLAAVSRVLSGVAQKPASRVLVASRTFANDATFEIKKCDLHRLEEGPPVTTVLTREDG

LKYYRMMQTVRRMELKADQLYKQKIIRGFCHLCDGQEACCVGLEAGINPTDHLITAYRAH

GFTFTRGLSVREILAELTGRRGGCGKGKGGSMHMYAKNFYGGNGIVGAQVPLGAGIALAC

KYNGKDEVCLTLYGDGAANQGQIFEAYNMAALWKLPCVFICENNRYGMGTSVERAAASTD

YYKRGDFIPGLRVDGMDILCVREATRFAAAYCRSGKGPILMELQTYRYHGHSMSDPGVSY

RTREEIQEVRSKSDPIMLLKDRMVNSNLASVEELKEIDVEVRKEIEDAAQFATADPEPPL

EELGYHIYCNDPPFEVRGANQWIKFKSIS

>sp|Q10489|ODPA_SCHPO Pyruvate dehydrogenase E1 component subunit alpha, mitochondrial OS=Schizosaccharomyces pombe (strain 972 / ATCC 24843) GN=pda1 PE=1 SV=1

MFRTCTKIGTVPKVLVNQKGLIDGLRRVTTDATTSRANPAHVPEEHDKPFPVKLDDSVFE

GYKIDVPSTEIEVTKGELLGLYEKMVTIRRLELACDALYKAKKIRGFCHLSIGQEAVAAG

IEGAITLDDSIITSYRCHGFAYTRGLSIRSIIGELMGRQCGASKGKGGSMHIFAKNFYGG

NGIVGAQIPLGAGIGFAQKYLEKPTTTFALYGDGASNQGQAFEAFNMAKLWGLPVIFACE

NNKYGMGTSAERSSAMTEFYKRGQYIPGLLVNGMDVLAVLQASKFAKKYTVENSQPLLME

FVTYRYGGHSMSDPGTTYRSREEVQKVRAARDPIEGLKKHIMEWGVANANELKNIEKRIR

GMVDEEVRIAEESPFPDPIEESLFSDVYVAGTEPAYARGRNSLEYHQYK

>sp|P52903|ODPA_SOLTU Pyruvate dehydrogenase E1 component subunit alpha, mitochondrial OS=Solanum tuberosum PE=1 SV=1

MALSTSRAINHIMKPLSAAVCATRRLSSDSTATITVETSLPFTSHNIDPPSRSVETSPKE

LMTFFKDMTEMRRMEIAADSLYKAKLIRGFCHLYDGQEAVAVGMEAAITKKDCIITAYRD

HCIFLGRGGTLVEAFAELMGRRDGCSRGKGGSMHFYKKESGFYGGHGIVGAQVPLGIGLA

FAQKYKKEDYVTFAMYGDGAANQGQLFEALNMAALWDLPAILVCENNHYGMGTAEWRAAK

SPAYYKRGDYVPGLRVDGMDVFAVKQACTFAKQHALKNGPIILEMDTYRYHGHSMSDPGS

TYRTRDEISGVRQERDPVERIRSLILAHNIATEAELKDIEKENRKVVDEAIAKAKESPMP

DPSELFTNVYVKGFGVEAYGADRKELRATLP

>sp|Q820A6|ODPA_STAAN Pyruvate dehydrogenase E1 component subunit alpha OS=Staphylococcus aureus (strain N315) GN=pdhA PE=1 SV=1

MAPKLQAQFDAVKVLNDTQSKFEMVQILDENGNVVNEDLVPDLTDEQLVELMERMVWTRI

LDQRSISLNRQGRLGFYAPTAGQEASQLASQYALEKEDYILPGYRDVPQIIWHGLPLTEA

FLFSRGHFKGNQFPEGVNALSPQIIIGAQYIQAAGVAFALKKRGKNAVAITYTGDGGSSQ

GDFYEGINFAAAYKAPAIFVIQNNNYAISTPRSKQTAAETLAQKAIAVGIPGIQVDGMDA

LAVYQATKEARDRAVAGEGPTLIETMTYRYGPHTMAGDDPTRYRTSDEDAEWEKKDPLVR

FRKFLENKGLWNEDKENEVIERAKADIKAAIKEADNTEKQTVTSLMEIMYEDMPQNLAEQ

YEIYKEKESK

>sp|O44451|ODPB_CAEEL Pyruvate dehydrogenase E1 component subunit beta, mitochondrial OS=Caenorhabditis elegans GN=pdhb-1 PE=1 SV=2

MALRKCGNLFVARLAGTSTRAASTMTVRDALNQAMDEEIKRDDRVFLMGEEVAQYDGAYK

ISKGLWKKHGDKRVVDTPITEMGFAGIAVGAAFAGLRPICEFMTFNFSMQAIDQIINSAA

KTYYMSAGRVPVPIVFRGPNGAAAGVAAQHSQDFSAWYAHCPGLKVVCPYSAEDAKGLLK

AAIRDDNPVVFLENEILYGQSFPVGDEVLSDDFVVPIGKAKIERAGDHVTIVSYSRGVEF

SLEAAKQLEAIGVSAEVINLRSLRPFDFESIRQSVHKTHHLVSVETGWPFAGIGSEIAAQ

VMESDVFDQLDAPLLRVTGVDVPMPYTQTLEAAALPTAEHVVKAVKKSLNIA

>sp|P11966|ODPB_BOVIN Pyruvate dehydrogenase E1 component subunit beta, mitochondrial OS=Bos taurus GN=PDHB PE=1 SV=2

MAVVAVLVRKPLEQVSGLLRRRFHRTAPAALQVTVREAINQGMDEELERDEKVFLLGEEV

AQYDGAYKVSRGLWKKYGDKRIIDTPISEMGFAGIAVGAAMAGLRPICEFMTFNFSMQAI

DQVINSAAKTYYMSGGLQSVPIVFRGPNGASAGVAAQHSQCFAAWYGHCPGLKVVSPWSS

EDAKGLIKSAIRDNNPVVVLENELMYGVPFELPSEAQSKDFLIPIGKAKIERQGTHVTIV

AHSRPVGHCLEAATVLSKEGIECEVINLRTIRPMDIETIEGSVMKTNHLVTVEGGWPQFG

VGAEICARIMEGPAFNFLDAPAVRVTGADVPMPYAKILEDNSVPQVKDIIFAIKKTLNI

>sp|Q86HX0|ODPB_DICDI Pyruvate dehydrogenase E1 component subunit beta, mitochondrial OS=Dictyostelium discoideum GN=pdhB PE=1 SV=1

MLSSILKKIQPSLLVNFRIITRTYATKEVTVRDAINSALDEELARDEKVFIMGEEVAQYN

GAYKITKGLFDKYGGDRIIDTPITEAGFAGIGVGAAMAGTRPIIEFMTFNFAMQAIDHII

NSSAKTHYMSGGKVFNPIVWRGPNGPPTAVGAQHSQCFAAWYGSVPGLKVVAPWSAADHR

GLLKSAIRDDNPVVYLESELLYNYKFDLSDQEQDKEYLVPIGKAKVEREGKDVTIVGFSR

IVSNCMEAAEILAKEGISAEVINLRTIRPIDAETIVNSLKKTNKLVTVEEGWAQSGIGAE

ISALMMEHAFDYLDAPIERICGADVPMPYASNLENAAMVQTQNIVNAAKRVTQRNK

>sp|P99063|ODPB_STAAN Pyruvate dehydrogenase E1 component subunit beta OS=Staphylococcus aureus (strain N315) GN=pdhB PE=1 SV=1

MAQMTMVQAINDALKTELKNDQDVLIFGEDVGVNGGVFRVTEGLQKEFGEDRVFDTPLAE

SGIGGLAMGLAVEGFRPVMEVQFLGFVFEVFDAIAGQIARTRFRSGGTKTAPVTIRSPFG

GGVHTPELHADNLEGILAQSPGLKVVIPSGPYDAKGLLISSIRSNDPVVYLEHMKLYRSF

REEVPEEEYTIDIGKANVKKEGNDISIITYGAMVQESMKAAEELEKDGYSVEVIDLRTVQ

PIDVDTIVASVEKTGRAVVVQEAQRQAGVGAAVVAELSERAILSLEAPIGRVAAADTIYP

FTQAENVWLPNKNDIIEKAKETLEF

>sp|P26269|ODPB_ASCSU Pyruvate dehydrogenase E1 component subunit beta, mitochondrial OS=Ascaris suum PE=1 SV=1

MAVNGCMRLLRNGLTSACALEQSVRRLASGTLNVTVRDALNAALDEEIKRDDRVFLIGEE

VAQYDGAYKISKGLWKKYGDGRIWDTPITEMAIAGLSVGAAMNGLRPICEFMSMNFSMQG

IDHIINSAAKAHYMSAGRFHVPIVFRGANGAAVGVAQQHSQDFTAWFMHCPGVKVVVPYD

CEDARGLLKAAVRDDNPVICLENEILYGMKFPVSPEAQSPDFVLPFGQAKIQRPGKDITI

VSLSIGVDVSLHAADELAKSGIDCEVINLRCVRPLDFQTVKDSVIKTKHLVTVESGWPNC

GVGAEISARVTESDAFGYLDGPILRVTGVDVPMPYAQPLETAALPQPADVVKMVKKCLNV

Q

>sp|P35488|ODPB_ACHLA Pyruvate dehydrogenase E1 component subunit beta OS=Acholeplasma laidlawii GN=pdhB PE=1 SV=1

MAIITLLEAINQAIDQAMEKDESIVVFGEDAGFEGGVFRVTAGLQKKYGETRVFDTPIAE

SAIVGSAVGMAINGLKPIAEIQFDGFIFPGYTDLVTHAARMRNRSRGQFTVPMVLRLPHG

GGIRALEHHSEALEVLFGSIPGLKVVTPSTPYDAKGLLLAAINDPDPVVFLEPKRIYRAG

KQEVPAEMYEIPIGKAKVVKQGTDMTVVAWGSIVREVEKAVKLVEAEGISVEIIDLRTIS

PIDEETILNSVKKTGKFMVVTEAVKSYGPAAELITMVNEKAFFHLEAAPVRFTGFDITVP

LARGEHYHFPQPEKIAAYIRKLAKARP

>sp|P86222|ODPB_MESAU Pyruvate dehydrogenase E1 component subunit beta, mitochondrial (Fragments) OS=Mesocricetus auratus GN=PDHB PE=1 SV=1

EAINQGMDEELERDEKVFLLGEEVAQYDGAYKVSRTYYMSAGLQPVPIVFRGPNGASAGV

AAQHSQCFAAWYGHCPGLKVVSPWNSEDAKGLIKSAIRDDNPVVMLENELMYGVAFELPT

EAQSKDFLIPIGKEGIECEVINLRTIRPMDIEAIEASVMKTNHLVTVEGGWPQFGVGAEI

CARIMEGPAFNFLDAPAVRVTGADVPMPYAK

>sp|P0A0A3|ODPB_STAAU Pyruvate dehydrogenase E1 component subunit beta OS=Staphylococcus aureus GN=pdhB PE=1 SV=1

MAQMTMVQAINDALKTELKNDQDVLIFGEDVGVNGGVFRVTEGLQKEFGEDRVFDTPLAE

SGIGGLAMGLAVEGFRPVMEVQFLGFVFEVFDAIAGQIARTRFRSGGTKTAPVTIRSPFG

GGVHTPELHADNLEGILAQSPGLKVVIPSGPYDAKGLLISSIRSNDPVVYLEHMKLYRSF

REEVPEEEYTIDIGKANVKKEGNDISIITYGAMVQESMKAAEELEKDGYSVEVIDLRTVQ

PIDVDTIVASVEKTGRAVVVQEAQRQAGVGAAVVAELSERAILSLEAPIGRVAAADTIYP

FTQAENVWLPNKNDIIEKAKETLEF

>sp|P49823|ODPA_CANFA Pyruvate dehydrogenase E1 component subunit alpha, somatic form (Fragment) OS=Canis familiaris GN=PDHA1 PE=1 SV=1

XXDATFEIKKXDL

>sp|P81419|ODPB_SOLTU Pyruvate dehydrogenase E1 component subunit beta, mitochondrial (Fragment) OS=Solanum tuberosum PE=1 SV=1

ISAVKEMTVRDALNSA

>sp|P10515|ODP2_HUMAN Dihydrolipoyllysine-residue acetyltransferase component of pyruvate dehydrogenase complex, mitochondrial OS=Homo sapiens GN=DLAT PE=1 SV=3

MWRVCARRAQNVAPWAGLEARWTALQEVPGTPRVTSRSGPAPARRNSVTTGYGGVRALCG

WTPSSGATPRNRLLLQLLGSPGRRYYSLPPHQKVPLPSLSPTMQAGTIARWEKKEGDKIN

EGDLIAEVETDKATVGFESLEECYMAKILVAEGTRDVPIGAIICITVGKPEDIEAFKNYT

LDSSAAPTPQAAPAPTPAATASPPTPSAQAPGSSYPPHMQVLLPALSPTMTMGTVQRWEK

KVGEKLSEGDLLAEIETDKATIGFEVQEEGYLAKILVPEGTRDVPLGTPLCIIVEKEADI

SAFADYRPTEVTDLKPQVPPPTPPPVAAVPPTPQPLAPTPSAPCPATPAGPKGRVFVSPL

AKKLAVEKGIDLTQVKGTGPDGRITKKDIDSFVPSKVAPAPAAVVPPTGPGMAPVPTGVF

TDIPISNIRRVIAQRLMQSKQTIPHYYLSIDVNMGEVLLVRKELNKILEGRSKISVNDFI

IKASALACLKVPEANSSWMDTVIRQNHVVDVSVAVSTPAGLITPIVFNAHIKGVETIAND

VVSLATKAREGKLQPHEFQGGTFTISNLGMFGIKNFSAIINPPQACILAIGASEDKLVPA

DNEKGFDVASMMSVTLSCDHRVVDGAVGAQWLAEFRKYLEKPITMLL

>sp|P06959|ODP2_ECOLI Dihydrolipoyllysine-residue acetyltransferase component of pyruvate dehydrogenase complex OS=Escherichia coli (strain K12) GN=aceF PE=1 SV=3

MAIEIKVPDIGADEVEITEILVKVGDKVEAEQSLITVEGDKASMEVPSPQAGIVKEIKVS

VGDKTQTGALIMIFDSADGAADAAPAQAEEKKEAAPAAAPAAAAAKDVNVPDIGSDEVEV

TEILVKVGDKVEAEQSLITVEGDKASMEVPAPFAGTVKEIKVNVGDKVSTGSLIMVFEVA

GEAGAAAPAAKQEAAPAAAPAPAAGVKEVNVPDIGGDEVEVTEVMVKVGDKVAAEQSLIT

VEGDKASMEVPAPFAGVVKELKVNVGDKVKTGSLIMIFEVEGAAPAAAPAKQEAAAPAPA

AKAEAPAAAPAAKAEGKSEFAENDAYVHATPLIRRLAREFGVNLAKVKGTGRKGRILRED

VQAYVKEAIKRAEAAPAATGGGIPGMLPWPKVDFSKFGEIEEVELGRIQKISGANLSRNW

VMIPHVTHFDKTDITELEAFRKQQNEEAAKRKLDVKITPVVFIMKAVAAALEQMPRFNSS

LSEDGQRLTLKKYINIGVAVDTPNGLVVPVFKDVNKKGIIELSRELMTISKKARDGKLTA

GEMQGGCFTISSIGGLGTTHFAPIVNAPEVAILGVSKSAMEPVWNGKEFVPRLMLPISLS

FDHRVIDGADGARFITIINNTLSDIRRLVM

>sp|P12695|ODP2_YEAST Dihydrolipoyllysine-residue acetyltransferase component of pyruvate dehydrogenase complex, mitochondrial OS=Saccharomyces cerevisiae (strain ATCC 204508 / S288c) GN=LAT1 PE=1 SV=1

MSAFVRVVPRISRSSVLTRSLRLQLRCYASYPEHTIIGMPALSPTMTQGNLAAWTKKEGD

QLSPGEVIAEIETDKAQMDFEFQEDGYLAKILVPEGTKDIPVNKPIAVYVEDKADVPAFK

DFKLEDSGSDSKTSTKAQPAEPQAEKKQEAPAEETKTSAPEAKKSDVAAPQGRIFASPLA

KTIALEKGISLKDVHGTGPRGRITKADIESYLEKSSKQSSQTSGAAAATPAAATSSTTAG

SAPSPSSTASYEDVPISTMRSIIGERLLQSTQGIPSYIVSSKISISKLLKLRQSLNATAN

DKYKLSINDLLVKAITVAAKRVPDANAYWLPNENVIRKFKNVDVSVAVATPTGLLTPIVK

NCEAKGLSQISNEIKELVKRARINKLAPEEFQGGTICISNMGMNNAVNMFTSIINPPQST

ILAIATVERVAVEDAAAENGFSFDNQVTITGTFDHRTIDGAKGAEFMKELKTVIENPLEM

LL

>sp|Q8NNJ2|ODP2_CORGL Dihydrolipoyllysine-residue acetyltransferase component of pyruvate dehydrogenase complex OS=Corynebacterium glutamicum (strain ATCC 13032 / DSM 20300 / JCM 1318 / LMG 3730 / NCIMB 10025) GN=aceF PE=1 SV=1

MAFSVEMPELGESVTEGTITQWLKSVGDTVEVDEPLLEVSTDKVDTEIPSPVAGVILEIK

AEEDDTVDVGGVIAIIGDADETPANEAPADEAPAPAEEEEPVKEEPKKEAAPEAPAATGA

ATDVEMPELGESVTEGTITQWLKAVGDTVEVDEPLLEVSTDKVDTEIPSPVAGTIVEILA

DEDDTVDVGAVIARIGDANAAAAPAEEEAAPAEEEEPVKEEPKKEAAPEAPAATGAATDV

EMPELGESVTEGTITQWLKAVGDTVEVDEPLLEVSTDKVDTEIPSPVAGTIVEILADEDD

TVDVGAVIARIGDANAAAAPAEEEAAPAEEEEPVKEEPKKEEPKKEEPKKEAATTPAAAS

ATVSASGDNVPYVTPLVRKLAEKHGVDLNTVTGTGIGGRIRKQDVLAAANGEAAPAEAAA

PVSAWSTKSVDPEKAKLRGTTQKVNRIREITAMKTVEALQISAQLTQLHEVDMTRVAELR

KKNKPAFIEKHGVNLTYLPFFVKAVVEALVSHPNVNASFNAKTKEMTYHSSVNLSIAVDT

PAGLLTPVIHDAQDLSIPEIAKAIVDLADRSRNNKLKPNDLSGGTFTITNIGSEGALSDT

PILVPPQAGILGTGAIVKRPVVITEDGIDSIAIRQMVFLPLTYDHQVVDGADAGRFLTTI

KDRLETANFEGDLQL

>sp|P10802|ODP2_AZOVI Dihydrolipoyllysine-residue acetyltransferase component of pyruvate dehydrogenase complex OS=Azotobacter vinelandii PE=1 SV=3

MSEIIRVPDIGGDGEVIELLVKTGDLIEVEQGLVVLESAKASMEVPSPKAGVVKSVSVKL

GDKLKEGDAIIELEPAAGAAAAPAEAAAVPAAPTQAVDEAEAPSPGASATPAPAAASQEV

RVPDIGSAGKARVIEVLVKAGDQVQAEQSLIVLESDKASMEIPSPASGVVESVAIQLNAE

VGTGDLILTLRTTGAQAQPTAPAAAAAASPAPAPLAPAAAGPQEVKVPDIGSAGKARVIE

VLVKAGDQVQAEQSLIVLESDKASMEIPSPAAGVVESVAVQLNAEVGTGDQILTLRVAGA

APSGPRARGSPGQAAAAPGAAPAPAPVGAPSRNGAKVHAGPAVRQLAREFGVELAAINST

GPRGRILKEDVQAYVKAMMQKAKEAPAAGAASGAGIPPIPPVDFAKYGEIEEVPMTRLMQ

IGATNLHRSWLNVPHVTQFESADITELEAFRVAQKAVAEKAGVKLTVLPLLLKACAYLLK

ELPDFNSSLAPSGQALIRKKYVHIGFAVDTPDGLLVPVIRNVDQKSLLQLAAEAAELAEK

ARSKKLGADAMQGACFTISSLGHIGGTAFTPIVNAPEVAILGVSKASMQPVWDGKAFQPR

LMLPLSLSYDHRVINGAAAARFTKRLGDLLADIRAILL

>sp|Q8BMF4|ODP2_MOUSE Dihydrolipoyllysine-residue acetyltransferase component of pyruvate dehydrogenase complex, mitochondrial OS=Mus musculus GN=Dlat PE=1 SV=2

MWRVCARRARSAVPRDGFRARWAALKEGPGAPCGSPRIGPAAVRCGSGIPRYGVRSLCGW

SSGSGTVPRNRLLRQLLGSPSRRSYSLPPHQKVPLPSLSPTMQAGTIARWEKKEGEKISE

GDLIAEVETDKATVGFESLEECYMAKILVPEGTRDVPVGSIICITVEKPQDIEAFKNYTL

DLAAAAAPQAAPAAAPAPAAAPAAPSASAPGSSYPTHMQIVLPALSPTMTMGTVQRWEKK

VGEKLSEGDLLAEIETDKATIGFEVQEEGYLAKILVPEGTRDVPLGAPLCIIVEKQEDIA

AFADYRPTEVTSLKPQAAPPAPPPVAAVPPTPQPVAPTPSAAPAGPKGRVFVSPLAKKLA

AEKGIDLTQVKGTGPEGRIIKKDIDSFVPSKAAPAAAAAMAPPGPRVAPAPAGVFTDIPI

SNIRRVIAQRLMQSKQTIPHYYLSVDVNMGEVLLVRKELNKMLEGKGKISVNDFIIKASA

LACLKVPEANSSWMDTVIRQNHVVDVSVAVSTPAGLITPIVFNAHIKGLETIASDVVSLA

SKAREGKLQPHEFQGGTFTISNLGMFGIKNFSAIINPPQACILAIGASEDKLIPADNEKG

FDVASVMSVTLSCDHRVVDGAVGAQWLAEFKKYLEKPITMLL

>sp|P9WIS7|ODP2_MYCTU Dihydrolipoyllysine-residue acetyltransferase component of pyruvate dehydrogenase complex OS=Mycobacterium tuberculosis (strain ATCC 25618 / H37Rv) GN=dlaT PE=1 SV=1

MAFSVQMPALGESVTEGTVTRWLKQEGDTVELDEPLVEVSTDKVDTEIPSPAAGVLTKII

AQEDDTVEVGGELAVIGDAKDAGEAAAPAPEKVPAAQPESKPAPEPPPVQPTSGAPAGGD

AKPVLMPELGESVTEGTVIRWLKKIGDSVQVDEPLVEVSTDKVDTEIPSPVAGVLVSISA

DEDATVPVGGELARIGVAADIGAAPAPKPAPKPVPEPAPTPKAEPAPSPPAAQPAGAAEG

APYVTPLVRKLASENNIDLAGVTGTGVGGRIRKQDVLAAAEQKKRAKAPAPAAQAAAAPA

PKAPPAPAPALAHLRGTTQKASRIRQITANKTRESLQATAQLTQTHEVDMTKIVGLRARA

KAAFAEREGVNLTFLPFFAKAVIDALKIHPNINASYNEDTKEITYYDAEHLGFAVDTEQG

LLSPVIHDAGDLSLAGLARAIADIAARARSGNLKPDELSGGTFTITNIGSQGALFDTPIL

VPPQAAMLGTGAIVKRPRVVVDASGNESIGVRSVCYLPLTYDHRLIDGADAGRFLTTIKH

RLEEGAFEADLGL

>sp|P11961|ODP2_GEOSE Dihydrolipoyllysine-residue acetyltransferase component of pyruvate dehydrogenase complex OS=Geobacillus stearothermophilus GN=pdhC PE=1 SV=3

MAFEFKLPDIGEGIHEGEIVKWFVKPGDEVNEDDVLCEVQNDKAVVEIPSPVKGKVLEIL

VPEGTVATVGQTLITLDAPGYENMTFKGQEQEEAKKEEKTETVSKEEKVDAVAPNAPAAE

AEAGPNRRVIAMPSVRKYAREKGVDIRLVQGTGKNGRVLKEDIDAFLAGGAKPAPAAAEE

KAAPAAAKPATTEGEFPETREKMSGIRRAIAKAMVHSKHTAPHVTLMDEADVTKLVAHRK

KFKAIAAEKGIKLTFLPYVVKALVSALREYPVLNTSIDDETEEIIQKHYYNIGIAADTDR

GLLVPVIKHADRKPIFALAQEINELAEKARDGKLTPGEMKGASCTITNIGSAGGQWFTPV

INHPEVAILGIGRIAEKPIVRDGEIVAAPMLALSLSFDHRMIDGATAQKALNHIKRLLSD

PELLLMEA

>sp|P08461|ODP2_RAT Dihydrolipoyllysine-residue acetyltransferase component of pyruvate dehydrogenase complex, mitochondrial OS=Rattus norvegicus GN=Dlat PE=1 SV=3

MWRVCARRVQSAVPRAGFRARWATLKGPRTGPAAVRCGSGIPSYGVRSLCGWSYGSATVP

RNRILQQLLGSPSRRSYSLPPHQKVPLPSLSPTMQAGTIARWEKKEGEKISEGDLIAEVE

TDKATVGFESLEECYMAKILVPEGTRDVPVGSIICITVEKPQDIEAFKNYTLDSATAATQ

AAPAPAAAPAAAPAAPSASAPGSSYPVHMQIVLPALSPTMTMGTVQRWEKKVGEKLSEGD

LLAEIETDKATIGFEVQEEGYLAKILVPEGTRDVPLGTPLCIIVEKQEDIAAFADYRPTE

VTSLKPQAPPPVPPPVAAVPPIPQPLAPTPSAAPAGPKGRVFVSPLAKKLAAEKGIDLTQ

VKGTGPEGRIIKKDIDSFVPTKAAPAAAAAAPPGPRVAPTPAGVFIDIPISNIRRVIAQR

LMQSKQTIPHYYLSVDVNMGEVLLVRKELNKMLEGKGKISVNDFIIKASALACLKVPEAN

SSWMDTVIRQNHVVDVSVAVSTPAGLITPIVFNAHIKGLETIASDVVSLASKAREGKLQP

HEFQGGTFTISNLGMFGIKNFSAIINPPQACILAIGASEDKLIPADNEKGFDVASVMSVT

LSCDHRVVDGAVGAQWLAEFKKYLEKPVTMLL

>sp|P21883|ODP2_BACSU Dihydrolipoyllysine-residue acetyltransferase component of pyruvate dehydrogenase complex OS=Bacillus subtilis (strain 168) GN=pdhC PE=1 SV=2

MAFEFKLPDIGEGIHEGEIVKWFVKPNDEVDEDDVLAEVQNDKAVVEIPSPVKGKVLELK

VEEGTVATVGQTIITFDAPGYEDLQFKGSDESDDAKTEAQVQSTAEAGQDVAKEEQAQEP

AKATGAGQQDQAEVDPNKRVIAMPSVRKYAREKGVDIRKVTGSGNNGRVVKEDIDSFVNG

GAQEAAPQETAAPQETAAKPAAAPAPEGEFPETREKMSGIRKAIAKAMVNSKHTAPHVTL

MDEVDVTNLVAHRKQFKQVAADQGIKLTYLPYVVKALTSALKKFPVLNTSIDDKTDEVIQ

KHYFNIGIAADTEKGLLVPVVKNADRKSVFEISDEINGLATKAREGKLAPAEMKGASCTI

TNIGSAGGQWFTPVINHPEVAILGIGRIAEKAIVRDGEIVAAPVLALSLSFDHRMIDGAT

AQNALNHIKRLLNDPQLILMEA

>sp|P20285|ODP2_NEUCR Dihydrolipoyllysine-residue acetyltransferase component of pyruvate dehydrogenase complex, mitochondrial OS=Neurospora crassa (strain ATCC 24698 / 74-OR23-1A / CBS 708.71 / DSM 1257 / FGSC 987) GN=mrp-3 PE=1 SV=2

MIVPVLSRQALRHASVARVALPSLTRWYASYPPHTVVKMPALSPTMTSGGIGAWQKKPGD

KIEPGEVLVEIETDKAQMDFEFQEEGVLAKILKDSGEKDVAVGNPIAILVEEGTDVNAFK

DFTLKDAGGETSPAVPKDEPKNESTASAPTPAPTPAPEPENTSFTGRFQTALEREPNALP

AAKRLAREKGIDLRNVKGSGPGGKITEEDVKKALASAPAAGAAAAAYTDVPISGMRKTIA

ARLKESVTENPHFFVSTNLSVSKLLKLRQALNSSADGRYKLSVNDFLIKAMGIASKRVPT

VNSSWRDGVIRQFETVDVSVAVATPNGLITPIVKGVEGKGLESISAAVKELAKKARDGKL

KPEEYQGGSISISNMGMNPAVQSFTAIINPPQAAILAVGAPQKVAVPVENEDGTTGVSWD

EQIIVTASFDHKVVDGAVGAEWIRELKKVIENPLELLL

>sp|P27747|ACOC_CUPNH Dihydrolipoyllysine-residue acetyltransferase component of acetoin cleaving system OS=Cupriavidus necator (strain ATCC 17699 / H16 / DSM 428 / Stanier 337) GN=acoC PE=1 SV=3

MATEISPTIIPIVMPKWGLSMKEGTVNAWLVDEGTEITVGLPILDVETDKIANAVEAPDA

GTLRRKVAQAGDVLPVKALLGVLAPAEVSDAQIDDYVAAYETPADDAGEEDAAAAYQFAD

VDGIRVRYARKGGGAETVLFIHGFGGDLDNWLFNLDPLADAYTVVALDLPGHGQSSPRLA

GTTLAQMAGFVARFMDETGIEAAHVVGHSMGGGVAAQLAVDAPQRVLSVALVSPVGFGDA

VNSGYTEGFVSAQSRRELKPVVELLFADAGLVSRQMLDDLLRYKRLDGVTEALTALGQGL

FGGGRQSEQPGQRLANSGKRVLVVWGGQDQIIPAAHAEAAPPGATVKVFADAGHMSQMEK

ANDFNALLKKHLGG

>sp|P35489|ODP2_ACHLA Dihydrolipoyllysine-residue acetyltransferase component of pyruvate dehydrogenase complex OS=Acholeplasma laidlawii GN=pdhC PE=1 SV=1

MYEFKFADIGEGIHEGTVLQWNFKVGDKVKEGETLVIVETDKVNAELPSPVDGTIVSLGA

KEGEEIHVGQIIVTIDDGTGTPAAAPAPAQVSAPTPAPAAAPQVAAPAASGDIYDFKFAD

IGEGIHEGTILQWNFKVGDKVKEGETLVVVETDKVNAELPSPVDGTILKLGKAEGEVIHV

GETVVLIGQNGATLEQAQAPKAEAPVSEPKKGAGVVGEIEVSDDIIGGSEEVHVVATTGK

VLASPVARKLASDLGVDIATIKGSGEQGRVMKDDVQNSKAPAEAQAPVQQTQAPAQAAAS

VAPSFAAAGKPQGDVEVVKITRLRKAVSNAMTRSKSIIPETVLMDEINVDALVNFRNEAK

GLAESKGIKLTYMAFIAKAVLIALKEFPMFNASFNHDTDEVYIKKFINLGMAVDTPDGLI

VPNIKNADRLSVFELASQVRSLADDTIARKISMDQQTGGTFTITNFGSAGIAFGTPVINY

PELAILGIGKIDRKPWVVGNEIKIAHTLPLSLAVDHRIIDGADGGRFLMRVKELLTNPTL

LLLS

>sp|Q19749|ODP2_CAEEL Dihydrolipoyllysine-residue acetyltransferase component of pyruvate dehydrogenase complex, mitochondrial OS=Caenorhabditis elegans GN=F23B12.5 PE=1 SV=1

MSKFPVPLRTIGGLRPSTTAAISAANIGFTQSSRALSTGAAAKSSGLVGQVARQYPNAAA

FSIKQVRLYSSGNLPKHNRVALPALSPTMELGTVVSWQKKEGDQLSEGDLLCEIETDKAT

MGFETPEEGYLAKILIQEGSKDVPIGKLLCIIVDNEADVAAFKDFKDDGASSGGSAPAAE

KAPEPAKPAASSQPSPPAQMYQAPSVPKSAPIPHSSSGRVSASPFAKKLAAENGLDLSGV

SGSGPGGRILASDLSQAPAKGATSTTTQAVSGQDYTDIPLSNMRKTIAKRLTESKSTIPH

YYLTSEIQLDTLLQVREKLNGLLAKGTSGQATKISINDFIIKASALACQRVPEANSYWMD

SFIRENHHVDVSVAVSTPAGLITPIIFNAHAKGLATIASEIVELAQRAREGKLQPHEFQG

GTFTVSNLGMFGSVSDFTAIINPPQSCILAIGGASDKLVPDEAEGYKKIKTMKVTLSCDH

RTVDGAVGAVWLRHFKEFLEKPHTMLL

>sp|P36413|ODP2_DICDI Dihydrolipoyllysine-residue acetyltransferase component of pyruvate dehydrogenase complex, mitochondrial OS=Dictyostelium discoideum GN=pdhC PE=1 SV=2

MLRAINQNSAKVVKSLKQQLVVLEATNVVAYTGTKSFTTTKTFNNTQTKPKIFTSSNVLS

FSSPSSSNVFSEILNKRSYSSKGKEITMPALSPSMTEGNIVQWKKKEGDQIKAGDVIAEV

ETDKATMDFQYEDGNGYLAKILIPEGTKGIEINKPIAIIVSKKEDIESAVKNYKPSSQAS

STPVQEEAPKPKQEAPKKSTKTYPAHKVVGMPALSPSMETGGIASWTKKEGDQIKAGDAI

AEVETDKATMDFQYEDGNGYLAKILVPGGTSGIQINQPVCIIVKNKEDCDKFADYSVEEQ

SSSSSSSSQESTPSSSSSSSQESTPSQSSSQQTTRKSGERIFATPAARFEASSKGYDLSA

INGTGPNNRILKADVLEFVPQKQEVAQQQQQQTTTTTKKPTTPTSSGEFTDIPHSNIRKV

TAARLTESKQTIPHYYLTMECRVDKLLKLRSELNAMNTVKISVNDFIVKASAAALRDNPV

VNSTWTDQFIRRYHNIDINVAVNTPQGLFTPIVRGVDMKGLNSISTSVKQLAEKAQNGKL

HPSEFESGTFTISNLGMLGIKQFAAVINPPQAAILAVGTTETRVVLSNKPDSPYETATIL

SVTLSCDHRVIDGAVGAEWLKSFKDYVENPIKLIL

>sp|P65636|ODP2_STAAN Dihydrolipoyllysine-residue acetyltransferase component of pyruvate dehydrogenase complex OS=Staphylococcus aureus (strain N315) GN=pdhC PE=1 SV=1

MAFEFRLPDIGEGIHEGEIVKWFVKAGDTIEEDDVLAEVQNDKSVVEIPSPVSGTVEEVM

VEEGTVAVVGDVIVKIDAPDAEDMQFKGHDDDSSSKEEPAKEEAPAEQAPVATQTEEVDE

NRTVKAMPSVRKYAREKGVNIKAVSGSGKNGRITKEDVDAYLNGGAPTASNESAASATSE

EVAETPAAPAAVSLEGDFPETTEKIPAMRRAIAKAMVNSKHTAPHVTLMDEIDVQALWDH

RKKFKEIAAEQGTKLTFLPYVVKALVSALKKYPALNTSFNEEAGEIVHKHYWNIGIAADT

DRGLLVPVVKHADRKSIFQISDEINELAVKARDGKLTADEMKGATCTISNIGSAGGQWFT

PVINHPEVAILGIGRIAQKPIVKDGEIVAAPVLALSLSFDHRQIDGATGQNAMNHIKRLL

NNPELLLMEG

>sp|P86197|ODP2_MESAU Dihydrolipoyllysine-residue acetyltransferase component of pyruvate dehydrogenase complex, mitochondrial (Fragments) OS=Mesocricetus auratus GN=DLAT PE=1 SV=1

VPLPSLSPTMQAGTIARDVPLGAPLCIIVEKGRVFVSPLAKGIDLTQVKGTGPEGDIDSF

VPSKVPEANSSWMDTVIRQNHVVDVSVAVSTPAGLITPIVFNAHIKGLETIASDVVSLAS

KEGKLQPHEFQGGTFTISNLGMFGIKNFSAIINPPQACILAIGASEDKLIPADNEKGFDV

ASVMSVTLSCDHRVVDGAVGAQWLAEFKKYLEKPITMLL

>sp|P11180|ODP2_BOVIN Dihydrolipoyllysine-residue acetyltransferase component of pyruvate dehydrogenase complex (Fragment) OS=Bos taurus GN=DLAT PE=1 SV=1

VETDKATVGF

>sp|P81421|ODP2_SOLTU 78 kDa dihydrolipoyllysine-residue acetyltransferase component of pyruvate dehydrogenase complex (Fragment) OS=Solanum tuberosum PE=1 SV=1

ISAEAPLYAEVGMPALSPTMT

>sp|P81420|ODP3_SOLTU 55 kDa dihydrolipoyllysine-residue acetyltransferase component of pyruvate dehydrogenase complex (Fragment) OS=Solanum tuberosum PE=1 SV=1

SSADSLPXHGAGXMP

>sp|P09622|DLDH_HUMAN Dihydrolipoyl dehydrogenase, mitochondrial OS=Homo sapiens GN=DLD PE=1 SV=2

MQSWSRVYCSLAKRGHFNRISHGLQGLSAVPLRTYADQPIDADVTVIGSGPGGYVAAIKA

AQLGFKTVCIEKNETLGGTCLNVGCIPSKALLNNSHYYHMAHGKDFASRGIEMSEVRLNL

DKMMEQKSTAVKALTGGIAHLFKQNKVVHVNGYGKITGKNQVTATKADGGTQVIDTKNIL

IATGSEVTPFPGITIDEDTIVSSTGALSLKKVPEKMVVIGAGVIGVELGSVWQRLGADVT

AVEFLGHVGGVGIDMEISKNFQRILQKQGFKFKLNTKVTGATKKSDGKIDVSIEAASGGK

AEVITCDVLLVCIGRRPFTKNLGLEELGIELDPRGRIPVNTRFQTKIPNIYAIGDVVAGP

MLAHKAEDEGIICVEGMAGGAVHIDYNCVPSVIYTHPEVAWVGKSEEQLKEEGIEYKVGK

FPFAANSRAKTNADTDGMVKILGQKSTDRVLGAHILGPGAGEMVNEAALALEYGASCEDI

ARVCHAHPTLSEAFREANLAASFGKSINF

>sp|Q9M5K2|DLDH2_ARATH Dihydrolipoyl dehydrogenase 2, mitochondrial OS=Arabidopsis thaliana GN=LPD2 PE=1 SV=1

MAMASLARRKAYFLTRNISNSPTDAFRFSFSLTRGFASSGSDDNDVVIIGGGPGGYVAAI

KAAQLGLKTTCIEKRGALGGTCLNVGCIPSKALLHSSHMYHEAKHVFANHGVKVSSVEVD

LPAMLAQKDTAVKNLTRGVEGLFKKNKVNYVKGYGKFLSPSEVSVDTIDGENVVVKGKHI

IVATGSDVKSLPGITIDEKKIVSSTGALSLTEIPKKLIVIGAGYIGLEMGSVWGRLGSEV

TVVEFAADIVPAMDGEIRKQFQRSLEKQKMKFMLKTKVVGVDSSGDGVKLIVEPAEGGEQ

TTLEADVVLVSAGRTPFTSGLDLEKIGVETDKGGRILVNERFSTNVSGVYAIGDVIPGPM

LAHKAEEDGVACVEFIAGKHGHVDYDKVPGVVYTYPEVASVGKTEEQLKKEGVSYNVGKF

PFMANSRAKAIDTAEGMVKILADKETDKILGVHIMSPNAGELIHEAVLAINYDASSEDIA

RVCHAHPTMSEAIKEAAMATYDKPIHM

>sp|P0A9P0|DLDH_ECOLI Dihydrolipoyl dehydrogenase OS=Escherichia coli (strain K12) GN=lpdA PE=1 SV=2

MSTEIKTQVVVLGAGPAGYSAAFRCADLGLETVIVERYNTLGGVCLNVGCIPSKALLHVA

KVIEEAKALAEHGIVFGEPKTDIDKIRTWKEKVINQLTGGLAGMAKGRKVKVVNGLGKFT

GANTLEVEGENGKTVINFDNAIIAAGSRPIQLPFIPHEDPRIWDSTDALELKEVPERLLV

MGGGIIGLEMGTVYHALGSQIDVVEMFDQVIPAADKDIVKVFTKRISKKFNLMLETKVTA

VEAKEDGIYVTMEGKKAPAEPQRYDAVLVAIGRVPNGKNLDAGKAGVEVDDRGFIRVDKQ

LRTNVPHIFAIGDIVGQPMLAHKGVHEGHVAAEVIAGKKHYFDPKVIPSIAYTEPEVAWV

GLTEKEAKEKGISYETATFPWAASGRAIASDCADGMTKLIFDKESHRVIGGAIVGTNGGE

LLGEIGLAIEMGCDAEDIALTIHAHPTLHESVGLAAEVFEGSITDLPNPKAKKK

>sp|P9WHH9|DLDH_MYCTU Dihydrolipoyl dehydrogenase OS=Mycobacterium tuberculosis (strain ATCC 25618 / H37Rv) GN=lpdC PE=1 SV=1

MTHYDVVVLGAGPGGYVAAIRAAQLGLSTAIVEPKYWGGVCLNVGCIPSKALLRNAELVH

IFTKDAKAFGISGEVTFDYGIAYDRSRKVAEGRVAGVHFLMKKNKITEIHGYGTFADANT

LLVDLNDGGTESVTFDNAIIATGSSTRLVPGTSLSANVVTYEEQILSRELPKSIIIAGAG

AIGMEFGYVLKNYGVDVTIVEFLPRALPNEDADVSKEIEKQFKKLGVTILTATKVESIAD

GGSQVTVTVTKDGVAQELKAEKVLQAIGFAPNVEGYGLDKAGVALTDRKAIGVDDYMRTN

VGHIYAIGDVNGLLQLAHVAEAQGVVAAETIAGAETLTLGDHRMLPRATFCQPNVASFGL

TEQQARNEGYDVVVAKFPFTANAKAHGVGDPSGFVKLVADAKHGELLGGHLVGHDVAELL

PELTLAQRWDLTASELARNVHTHPTMSEALQECFHGLVGHMINF

>sp|P09624|DLDH_YEAST Dihydrolipoyl dehydrogenase, mitochondrial OS=Saccharomyces cerevisiae (strain ATCC 204508 / S288c) GN=LPD1 PE=1 SV=1

MLRIRSLLNNKRAFSSTVRTLTINKSHDVVIIGGGPAGYVAAIKAAQLGFNTACVEKRGK

LGGTCLNVGCIPSKALLNNSHLFHQMHTEAQKRGIDVNGDIKINVANFQKAKDDAVKQLT

GGIELLFKKNKVTYYKGNGSFEDETKIRVTPVDGLEGTVKEDHILDVKNIIVATGSEVTP

FPGIEIDEEKIVSSTGALSLKEIPKRLTIIGGGIIGLEMGSVYSRLGSKVTVVEFQPQIG

ASMDGEVAKATQKFLKKQGLDFKLSTKVISAKRNDDKNVVEIVVEDTKTNKQENLEAEVL

LVAVGRRPYIAGLGAEKIGLEVDKRGRLVIDDQFNSKFPHIKVVGDVTFGPMLAHKAEEE

GIAAVEMLKTGHGHVNYNNIPSVMYSHPEVAWVGKTEEQLKEAGIDYKIGKFPFAANSRA

KTNQDTEGFVKILIDSKTERILGAHIIGPNAGEMIAEAGLALEYGASAEDVARVCHAHPT

LSEAFKEANMAAYDKAIHC

>sp|Q9M5K3|DLDH1_ARATH Dihydrolipoyl dehydrogenase 1, mitochondrial OS=Arabidopsis thaliana GN=LPD1 PE=1 SV=2

MAMASLARRKAYFLTRNLSNSPTDALRFSFSLSRGFASSGSDENDVVIIGGGPGGYVAAI

KASQLGLKTTCIEKRGALGGTCLNVGCIPSKALLHSSHMYHEAKHSFANHGIKVSSVEVD

LPAMLAQKDNAVKNLTRGIEGLFKKNKVTYVKGYGKFISPNEVSVETIDGGNTIVKGKHI

IVATGSDVKSLPGITIDEKKIVSSTGALSLSEVPKKLIVIGAGYIGLEMGSVWGRLGSEV

TVVEFAGDIVPSMDGEIRKQFQRSLEKQKMKFMLKTKVVSVDSSSDGVKLTVEPAEGGEQ

SILEADVVLVSAGRTPFTSGLDLEKIGVETDKAGRILVNDRFLSNVPGVYAIGDVIPGPM

LAHKAEEDGVACVEFIAGKHGHVDYDKVPGVVYTHPEVASVGKTEEQLKKEGVSYRVGKF

PFMANSRAKAIDNAEGLVKILADKETDKILGVHIMAPNAGELIHEAVLAINYDASSEDIA

RVCHAHPTMSEALKEAAMATYDKPIHI

>sp|Q8NTE1|DLDH_CORGL Dihydrolipoyl dehydrogenase OS=Corynebacterium glutamicum (strain ATCC 13032 / DSM 20300 / JCM 1318 / LMG 3730 / NCIMB 10025) GN=lpd PE=1 SV=1

MTEHYDVVVLGAGPGGYVSAIRAAQLGKKVAVIEKQYWGGVCLNVGCIPSKSLIKNAEVA

HTFTHEKKTFGINGEVTFNYEDAHKRSRGVSDKIVGGVHYLMKKNKIIEIHGLGNFKDAK

TLEVTDGKDAGKTITFDDCIIATGSVVNTLRGVDFSENVVSFEEQILNPVAPKKMVIVGA

GAIGMEFAYVLGNYGVDVTVIEFMDRVLPNEDAEVSKVIAKAYKKMGVKLLPGHATTAVR

DNGDFVEVDYQKKGSDKTETLTVDRVMVSVGFRPRVEGFGLENTGVKLTERGAIEIDDYM

RTNVDGIYAIGDVTAKLQLAHVAEAQGIVAAETIAGAETQTLGDYMMMPRATFCNPQVSS

FGYTEEQAKEKWPDREIKVASFPFSANGKAVGLAETDGFAKIVADAEFGELLGAHLVGAN

ASELINELVLAQNWDLTTEEISRSVHIHPTLSEAVKEAAHGISGHMINF

>sp|O08749|DLDH_MOUSE Dihydrolipoyl dehydrogenase, mitochondrial OS=Mus musculus GN=Dld PE=1 SV=2

MQSWSRVYRSLAKKGHFNRISHGLQGVSSVPLRTYADQPIEADVTVIGSGPGGYVAAIKS

AQLGFKTVCIEKNETLGGTCLNVGCIPSKALLNNSHYYHMAHGKDFASRGIEIPEVRLNL

EKMMEQKHSAVKALTGGIAHLFKQNKVVHVNGFGKITGKNQVTATKADGSTQVIDTKNIL

VATGSEVTPFPGITIDEDTIVSSTGALSLKKVPEKLVVIGAGVIGVELGSVWQRLGADVT

AVEFLGHVGGIGIDMEISKNFQRILQRQGFKFKLNTKVTGATKKSDGKIDVSVEAASGGK

AEVITCDVLLVCIGRRPFTQNLGLEELGIELDPKGRIPVNNRFQTKIPNIYAIGDVVAGP

MLAHKAEDEGIICVEGMAGGAVHIDYNCVPSVIYTHPEVAWVGKSEEQLKEEGIEFKIGK

FPFAANSRAKTNADTDGMVKILGHKSTDRVLGAHILGPGAGEMVNEAALALEYGASCEDI

ARVCHAHPTLSEAFREANLAAAFGKPINF

>sp|P11959|DLDH1_GEOSE Dihydrolipoyl dehydrogenase OS=Geobacillus stearothermophilus GN=pdhD PE=1 SV=2

MVVGDFAIETETLVVGAGPGGYVAAIRAAQLGQKVTIVEKGNLGGVCLNVGCIPSKALIS

ASHRYEQAKHSEEMGIKAENVTIDFAKVQEWKASVVKKLTGGVEGLLKGNKVEIVKGEAY

FVDANTVRVVNGDSAQTYTFKNAIIATGSRPIELPNFKFSNRILDSTGALNLGEVPKSLV

VIGGGYIGIELGTAYANFGTKVTILEGAGEILSGFEKQMAAIIKKRLKKKGVEVVTNALA

KGAEEREDGVTVTYEANGETKTIDADYVLVTVGRRPNTDELGLEQIGIKMTNRGLIEVDQ

QCRTSVPNIFAIGDIVPGPALAHKASYEGKVAAEAIAGHPSAVDYVAIPAVVFSDPECAS

VGYFEQQAKDEGIDVIAAKFPFAANGRALALNDTDGFLKLVVRKEDGVIIGAQIIGPNAS

DMIAELGLAIEAGMTAEDIALTIHAHPTLGEIAMEAAEVALGTPIHIITK

>sp|P09063|DLDH1_PSEPU Dihydrolipoyl dehydrogenase OS=Pseudomonas putida GN=lpdV PE=1 SV=1

MQQTIQTTLLIIGGGPGGYVAAIRAGQLGIPTVLVEGQALGGTCLNIGCIPSKALIHVAE

QFHQASRFTEPSPLGISVASPRLDIGQSVAWKDGIVDRLTTGVAALLKKHGVKVVHGWAK

VLDGKQVEVDGQRIQCEHLLLATGSSSVELPMLPLGGPVISSTEALAPKALPQHLVVVGG

GYIGLELGIAYRKLGAQVSVVEARERILPTYDSELTAPVAESLKKLGIALHLGHSVEGYE

NGCLLANDGKGGQLRLEADRVLVAVGRRPRTKGFNLECLDLKMNGAAIAIDERCQTSMHN

VWAIGDVAGEPMLAHRAMAQGEMVAEIIAGKARRFEPAAIAAVCFTDPEVVVVGKTPEQA

SQQGLDCIVAQFPFAANGRAMSLESKSGFVRVVARRDNHLILGWQAVGVAVSELSTAFAQ

SLEMGACLEDVAGTIHAHPTLGEAVQEAALRALGHALHI

>sp|P18925|DLDH_AZOVI Dihydrolipoyl dehydrogenase OS=Azotobacter vinelandii PE=1 SV=1

MSQKFDVIVIGAGPGGYVAAIKSAQLGLKTALIEKYKGKEGKTALGGTCLNVGCIPSKAL

LDSSYKFHEAHESFKLHGISTGEVAIDVPTMIARKDQIVRNLTGGVASLIKANGVTLFEG

HGKLLAGKKVEVTAADGSSQVLDTENVILASGSKPVEIPPAPVDQDVIVDSTGALDFQNV

PGKLGVIGAGVIGLELGSVWARLGAEVTVLEAMDKFLPAVDEQVAKEAQKILTKQGLKIL

LGARVTGTEVKNKQVTVKFVDAEGEKSQAFDKLIVAVGRRPVTTDLLAADSGVTLDERGF

IYVDDYCATSVPGVYAIGDVVRGAMLAHKASEEGVVVAERIAGHKAQMNYDLIPAVIYTH

PEIAGVGKTEQALKAEGVAINVGVFPFAASGRAMAANDTAGFVKVIADAKTDRVLGVHVI

GPSAAELVQQGAIAMEFGTSAEDLGMMVFAHPALSEALHEAALAVSGHAIHVANRKK

>sp|P49819|DLDH_CANFA Dihydrolipoyl dehydrogenase, mitochondrial OS=Canis familiaris GN=DLD PE=1 SV=1

MQSWSRVYCSLAKRGHFSRISHGLQAVSAVPLRTYADQPIDADVTVIGSGPGGYVAAIKA

AQLGFKTVCVEKNETLGGTCLNVGCIPSKALLNNSHYYHMAHGKDFASRGIEMSEVRLNL

EKMMEQKSTAVKALTGGIAHLFKQNKVVHVNGYGKITGKNQVTAKKADGSTQVIDTKNIL

IATGSEVTPFPGITIDEDTIVSSTGALSLKKVPEKMVVIGAGVIGVELGSVWQRLGADVT

AVEFLGHVGGVGIDMEISKNFQRILQKQGFKFKLNTKVTGATKKSDGKIDVSIEGASGGK

AEVITCDVLLVCIGRRPFTQNLGLEELGIELDPRGRIPVNTRFQTKIPNIYAIGDVVAGP

MLAHKAEDEGIICVEGMAGGAVPIDYNCVPSVIYTHPEVAWVGKSEEQLKEEGIEYKVGK

FPFAANSRAKTNADTDGMVKILGQKSTDRVLGAHILGPGAGEMVNEAALALEYGASCEDI

ARVCHAHPTLSEAFREANLAASFGKSINF

>sp|Q04829|DLDH_HALVD Dihydrolipoyl dehydrogenase OS=Haloferax volcanii (strain ATCC 29605 / DSM 3757 / JCM 8879 / NBRC 14742 / NCIMB 2012 / VKM B-1768 / DS2) GN=lpdA PE=1 SV=3

MVVGDIATGTELLVIGAGPGGYVAAIRAAQNGIDTTLVEKDAYGGTCLNYGCIPSKALIT

GANLAHEAGNAEEMGIHADPVVDMSQLRDWKSGVVDQLTGGVEKLCKANGVNLVEGTARF

KDENAVRIAHGGEGQGSETIEFEHCIIATGSRVIQIPGFDFGDEPVWSSRDALEADTVPE

RLVVVGGGYIGMELSTTFAKLGADVTVVEMLDDILPGYESDVARVVRKRAEELGIDMHLG

EGASGWREEDDGIMVTTETEDGEENEYRADKVLVAVGRSPVTDTMDIENAGLEADDRGFL

SVDDRRRTDVEHIYAVGDVVEDTPMLAHVASKEGIVAAEHVAGEPVAFDSQAVPAAVFTD

PEIGTVGMTEADAEEAGFTPVVGQMPFRASGRALTTNHADGFVRVVADEESGFVLGAQIV

GPEASELIAELAFAIEMGATLEDVASTIHTHPTLAEAVMEAAENALGQAIHTLNR

>sp|P31023|DLDH_PEA Dihydrolipoyl dehydrogenase, mitochondrial OS=Pisum sativum GN=LPD PE=1 SV=2

MAMANLARRKGYSLLSSETLRYSFSLRSRAFASGSDENDVVIIGGGPGGYVAAIKAAQLG

FKTTCIEKRGALGGTCLNVGCIPSKALLHSSHMYHEAKHSFANHGVKVSNVEIDLAAMMG

QKDKAVSNLTRGIEGLFKKNKVTYVKGYGKFVSPSEISVDTIEGENTVVKGKHIIIATGS

DVKSLPGVTIDEKKIVSSTGALALSEIPKKLVVIGAGYIGLEMGSVWGRIGSEVTVVEFA

SEIVPTMDAEIRKQFQRSLEKQGMKFKLKTKVVGVDTSGDGVKLTVEPSAGGEQTIIEAD

VVLVSAGRTPFTSGLNLDKIGVETDKLGRILVNERFSTNVSGVYAIGDVIPGPMLAHKAE

EDGVACVEYLAGKVGHVDYDKVPGVVYTNPEVASVGKTEEQVKETGVEYRVGKFPFMANS

RAKAIDNAEGLVKIIAEKETDKILGVHIMAPNAGELIHEAAIALQYDASSEDIARVCHAH

PTMSEAIKEAAMATYDKPIHI

>sp|P09623|DLDH_PIG Dihydrolipoyl dehydrogenase, mitochondrial OS=Sus scrofa GN=DLD PE=1 SV=1

MQSWSRVYCTLAKRGHFNRIAHGLQGVSAVPLRTYADQPIDADVTVIGSGPGGYVAAIKA

AQLGFKTVCIEKNETLGGTCLNVGCIPSKALLNNSHYYHMAHGKDFASRGIEMSEVRLNL

EKMMEQKSNAVKALTGGIAHLFKQNKVVRVNGYGKITGKNQVTATKADGSTEVINTKNIL

IATGSEVTPFPGITIDEDTVVSSTGALSLKKVPEKMVVIGAGVIGVELGSVWQRLGADVT

AVELLGHVGGIGIDMEVSKNFQRILQKQGFKFKLNTKVIGATKKSDGNIDVSIEAASGGK

AEVITCDVLLVCIGRRPFTQNLGLEELGIELDPRGRIPVNTRFQTKIPNIYAIGDVVAGP

MLAHKAEDEGIICVEGMAGGAVHIDYNCVPSVIYTHPEVAWVGKSEEQLKEEGIEYKVGK

FPFAANSRAKTNADTDGMVKILGQKSTDRVLGAHIIGPGAGEMINEAALALEYGASCEDI

ARVCHAHPTLSEAFREANLAASFGKAINF

>sp|Q9I1L9|DLDH1_PSEAE Dihydrolipoyl dehydrogenase OS=Pseudomonas aeruginosa (strain ATCC 15692 / PAO1 / 1C / PRS 101 / LMG 12228) GN=lpdV PE=1 SV=1

MSQILKTSLLIVGGGPGGYVAAIRAGQLGIPTVLVEGAALGGTCLNVGCIPSKALIHAAE

EYLKARHYASRSALGIQVQAPSIDIARTVEWKDAIVDRLTSGVAALLKKHGVDVVQGWAR

ILDGKSVAVELAGGGSQRIECEHLLLAAGSQSVELPILPLGGKVISSTEALAPGSLPKRL

VVVGGGYIGLELGTAYRKLGVEVAVVEAQPRILPGYDEELTKPVAQALRRLGVELYLGHS

LLGPSENGVRVRDGAGEEREIAADQVLVAVGRKPRSEGWNLESLGLDMNGRAVKVDDQCR

TSMRNVWAIGDLAGEPMLAHRAMAQGEMVAELIAGKRRQFAPVAIPAVCFTDPEVVVAGL

SPEQAKDAGLDCLVASFPFAANGRAMTLEANEGFVRVVARRDNHLVVGWQAVGKAVSELS

TAFAQSLEMGARLEDIAGTIHAHPTLGEAVQEAALRALGHALHI

>sp|P31046|DLDH3_PSEPU Dihydrolipoyl dehydrogenase 3 OS=Pseudomonas putida GN=lpd3 PE=1 SV=1

MKSYDVVIIGGGPGGYNAAIRAGQLGLTVACVEGRSTLGGTCLNVGCMPSKALLHASELY

EAASGDEFAHLGIEVKPTLNLAQMMKQKDESVTGLTKGIEYLFRKNKVDWIKGWGRLDGV

GKVVVKAEDGSETALQAKDIVIATGSEPTPLPGVTIDNQRIIDSTGALSLPQVPKHLVVI

GAGVIGLELGSVWRRLGSQVTVIEYLDRICPGTDTETAKTLQKALAKQGMVFKLGSKVTQ

ATASADGVSLVLEPAAGGTAESLQADYVLVAIGRRPYTKGLNLESVGLETDKRGMLAQRT

PPTSVPGVWVIGDVTSGPMLAHKAEDEAVACIERIAGKPHEVNYNLIPGVIYTRPELATV

GKTEEQLKAEGRAYKVGKFPFTANSRAKINHETEGFAKVIADAETDEVLGVHLVGPSVSE

MIGEFCVAMEFSASAEDIALTCHPHPTRSEALRQAAMNVDGMAMQI

>sp|P31052|DLDH2_PSEPU Dihydrolipoyl dehydrogenase OS=Pseudomonas putida GN=lpdG PE=1 SV=4

MTQKFDVVVIGAGPGGYVAAIKAAQLGLKTACIEKYTDAEGKLALGGTCLNVGCIPSKAL

LDSSWKYKEAKESFNVHGISTGEVKMDVAAMVGRKAGIVKNLTGGVATLFKANGVTSIQG

HGKLLAGKKVEVTKADGTTEVIEAENVILASGSRPIDIPPAPVDQNVIVDSTGALEFQAV

PKRLGVIGAGVIGLELGSVWARLGAEVTVLEALDTFLMAADTAVSKEAQKTLTKQGLDIK

LGARVTGSKVNGNEVEVTYTNAEGEQKITFDKLIVAVGRRPVTTDLLAADSGVTIDERGY

IFVDDYCATSVPGVYAIGDVVRGMMLAHKASEEGIMVVERIKGHKAQMNYDLIPSVIYTH

PEIAWVGKTEQALKAEGVEVNVGTFPFAASGRAMAANDTGGFVKVIADAKTDRVLGVHVI

GPSAAELVQQGAIAMEFGTSAEDLGMMVFSHPTLSEALHEAALAVNGGAIHVANRKKR

>sp|Q811C4|DLDH_MESAU Dihydrolipoyl dehydrogenase, mitochondrial (Fragment) OS=Mesocricetus auratus GN=DLD PE=1 SV=1

FNRXSPGLQGVSSVPLRTYADQPIDADVTVIGSGPGGYVAAIKAAQLGFKTVCIEKNETL

GGTCLNVGCIPSKALLNNSHYYHLAHGKDFASRGIELSEVRLNLEKMMEQKSSAVKALTG

GIAHLFKQNKVVHVNGFGNITGKNQVTATKADGSSQVIGTKNILIATGSEVTPFPGITID

EDTIVSSTGALSLKKVPEKLVVIGAGVIGVELGSVWQRLGAEVTAVEFLGHVGGIGIDME

ISKKFQRILQKQGFKFKLNPKVPGATKRSDGKIDVSVEAAPGGKAEVIPCDVLLVCIGRR

PFTQNLGLEELGIELDPRGRIPVNTRFQTKIPNIYAIGDVVAGPMLAHKAEDEGIICVEG

MAGGAVHIDYNCVPSVIYTHPEVAWVGKSEEQLKEEGIEYKVGKFPFAANSRAKTNADTD

GMVKILGQKSTDRVLGAHILGPGAGEMVNEAALALEYGASCEDIARVCHAHPTLSEAFR

>sp|P14218|DLDH_PSEFL Dihydrolipoyl dehydrogenase OS=Pseudomonas fluorescens GN=lpd PE=1 SV=3

MSQKFDVVVIGAGPGGYVAAIRAAQLGLKTACIEKYIGKEGKVALGGTCLNVGCIPSKAL

LDSSYKYHEAKEAFKVHGIEAKGVTIDVPAMVARKANIVKNLTGGIATLFKANGVTSFEG

HGKLLANKQVEVTGLDGKTQVLEAENVIIASGSRPVEIPPAPLSDDIIVDSTGALEFQAV

PKKLGVIGAGVIGLELGSVWARLGAEVTVLEALDKFLPAADEQIAKEALKVLTKQGLNIR

LGARVTASEVKKKQVTVTFTDANGEQKETFDKLIVAVGRRPVTTDLLAADSGVTLDERGF

IYVDDHCKTSVPGVFAIGDVVRGAMLAHKASEEGVMVAERIAGHKAQMNYDLIPSVIYTH

PEIAWVGKTEQTLKAEGVEVNVGTFPFAASGRAMAANDTTGLVKVIADAKTDRVLGVHVI

GPSAAELVQQGAIGMEFGTSAEDLGMMVFSHPTLSEALHEAALAVNGHAIHIANRKKR

>sp|Q6P6R2|DLDH_RAT Dihydrolipoyl dehydrogenase, mitochondrial OS=Rattus norvegicus GN=Dld PE=1 SV=1

MQSWSRVYCSLAKKGHFNRLSHGLQGASSVPLRTYSDQPIDADVTVIGSGPGGYVAAIKA

AQLGFKTVCIEKNETLGGTCLNVGCIPSKALLNNSHYYHLAHGKDFASRGIEIPEVRLNL

EKMMEQKRSAVKALTGGIAHLFKQNKVVHVNGFGKITGKNQVTATTADGSTQVIGTKNIL

IATGSEVTPFPGITIDEDTIVSSTGALSLKKVPEKLVVIGAGVIGVELGSVWQRLGADVT

AVEFLGHVGGIGIDMEISKNFQRILQKQGFKFKLNTKVTGATKKSDGKIDVSVEAASGGK

AEVITCDVLLVCIGRRPFTQNLGLEELGIELDPKGRIPVNTRFQTKIPNIFAIGDVVAGP

MLAHKAEDEGIICVEGMAGGAVHIDYNCVPSVIYTHPEVAWVGKSEEQLKEEGVEFKVGK

FPFAANSRAKTNADTDGMVKILGHKSTDRILGAHILGPGAGEMVNEAALALEYGASCEDV

ARVCHAHPTLSEAFREANLAASFGKPINF

>sp|P99084|DLDH_STAAN Dihydrolipoyl dehydrogenase OS=Staphylococcus aureus (strain N315) GN=pdhD PE=1 SV=1

MVVGDFPIETDTIVIGAGPGGYVAAIRAAQLGQKVTIVEKGNLGGVCLNVGCIPSKALLH

ASHRFVEAQHSENLGVIAESVSLNFQKVQEFKSSVVNKLTGGVEGLLKGNKVNIVKGEAY

FVDNNSLRVMDEKSAQTYNFKNAIIATGSRPIEIPNFKFGKRVIDSTGALNLQEVPGKLV

VVGGGYIGSELGTAFANFGSEVTILEGAKDILGGFEKQMTQPVKKGMKEKGVEIVTEAMA

KSAEETDNGVKVTYEAKGEEKTIEADYVLVTVGRRPNTDELGLEELGVKFADRGLLEVDK

QSRTSISNIYAIGDIVPGLPLAHKASYEAKVAAEAIDGQAAEVDYIGMPAVCFTEPELAT

VGYSEAQAKEEGLAIKASKFPYAANGRALSLDDTNGFVKLITLKEDDTLIGAQVVGTGAS

DIISELGLAIEAGMNAEDIALTIHAHPTLGEMTMEAAEKAIGYPIHTM

>sp|P72740|DLDH_SYNY3 Dihydrolipoyl dehydrogenase OS=Synechocystis sp. (strain PCC 6803 / Kazusa) GN=lpdA PE=1 SV=3

MSQDFDYDLVIIGAGVGGHGAALHAVKCGLKTAIIEAKDMGGTCVNRGCIPSKALLAASG

RVREMSDQDHLQQLGIQINGVTFTREAIAAHANDLVSKIQSDLTNSLTRLKVDTIRGWGK

VSGPQEVTVIGDNETRILKAKEIMLCPGSVPFVPPGIEIDHKTVFTSDEAVKLETLPQWI

AIIGSGYIGLEFSDVYTALGCEVTMIEALPDLMPGFDPEIAKIAERVLIKSRDIETYTGV

FATKIKAGSPVEIELTDAKTKEVIDTLEVDACLVATGRIPATKNLGLETVGVETDRRGFI

EVNDQMQVIKDGKPVPHLWAVGDATGKMMLAHAASGQGVVAVENICGRKTEVDYRAIPAA

AFTHPEISYVGLTEAQAKELGEKEGFVVSTAKTYFKGNSKALAEKETDGIAKVVYRQDTG

ELLGAHIIGIHASDLIQEAAQAIADRKSVRELAFHVHAHPTLSEVLDEAYKRAV

>sp|P85207|DLDH_THESS Dihydrolipoyl dehydrogenase OS=Thermus scotoductus (strain ATCC 700910 / SA-01) GN=lpd PE=1 SV=2

MKTYDLIVIGTGPGGYPAAIRGAQLGLKVLAVEAAEVGGVCLNVGCIPTKALLHAAETVH

HLKGAEGFGLKAKPELDLKKLGAWRDGVVKKLTGGVAGLLKGNKVELLRGFARFKGPREI

EVNGETYGAQSFIIATGSEPMPLKGFPFGEDVWDSTRALRVEEGIPKRLLVIGGGAVGLE

LGQIYHRLGSEVTLIEYMPEILPAGDRETAALLRKALEKEGLKVRTGTKAVGYEKKQDGL

HVLLEAAQGGSQEEIVVDKILVAVGRRPRTEGLGLEKAGVKVDERGFIQVNARMETSAPG

VYAIGDVARPPLLAHKAMKEGLVAAENAAGKNALFDFQVPSVVYTGPEWAGVGLTEEEAR

KAGYNVKVGKFPFSASGRALTLGGAEGLIKVVGDAETDLLLGVFVVGPQAGELIAEATLA

LEMGATVSDLGLTIHPHPTLSEGLMEAAEALHKQAIHILNR

>sp|P90597|DLDH_TRYCR Dihydrolipoyl dehydrogenase OS=Trypanosoma cruzi GN=LPD PE=1 SV=1

MFRRCAVKLNPYDVVVIGGGPGGYVASIKAAQLGMKTACVEKRGALGGTCLNVGCIPSKA

LLHATHVYHDAHANFARYGLMGGEGVTMDSAKMQQQKERAVKGLTGGVEYLFKKNKVTYY

KGEGSFETAHSIRVNGLDGKQEMFETKKTIIATGSEPTELPFLPFDEKVVLSSTGALALP

RVPKTMVVIGGGVIGLELGSVWARLGAKVTVVEFAPRCAPTLDEDVTNALVGALAKNEKM

KFMTSTKVVGGTNNGDSVSLEVEGKNGKRETVTCEALLVSVGRRPFTGGLGLDKINVAKN

ERGFVKIGDHFETSIPDVYAIGDVVDKGPMLAHKAEDEGVACAEILAGKPGHVNYGVIPA

VIYTMPEVASVGKSEEELKKEGVAYKVGKFPFNANSRAKAVSTEDGFVKVLVDKATDRIL

GVHIVCTTAGELIGEACLAMEYGASSEDVGRTCHAHPTMSEALKEACMALVAKTINF

>sp|P80503|DLDH_SOLTU Dihydrolipoyl dehydrogenase (Fragment) OS=Solanum tuberosum PE=1 SV=1

ASGSDENDVVVIGGGPGGYVAAIKAAQLGLKTTXIEKRGT

>sp|P80647|DLDH_HYMDI Dihydrolipoyl dehydrogenase (Fragment) OS=Hymenolepis diminuta PE=1 SV=1

LSSGEKDLVVIGSGPGGYVAAIKAAQLGMLTVCIEKYPTFGGTCLNVGCIPSK

>sp|P84545|DLDH_POPEU Dihydrolipoyl dehydrogenase (Fragment) OS=Populus euphratica PE=1 SV=1

VGKFPLLANSR

>sp|P0A9M8|PTA_ECOLI Phosphate acetyltransferase OS=Escherichia coli (strain K12) GN=pta PE=1 SV=2

MSRIIMLIPTGTSVGLTSVSLGVIRAMERKGVRLSVFKPIAQPRTGGDAPDQTTTIVRAN

SSTTTAAEPLKMSYVEGLLSSNQKDVLMEEIVANYHANTKDAEVVLVEGLVPTRKHQFAQ

SLNYEIAKTLNAEIVFVMSQGTDTPEQLKERIELTRNSFGGAKNTNITGVIVNKLNAPVD

EQGRTRPDLSEIFDDSSKAKVNNVDPAKLQESSPLPVLGAVPWSFDLIATRAIDMARHLN

ATIINEGDINTRRVKSVTFCARSIPHMLEHFRAGSLLVTSADRPDVLVAACLAAMNGVEI

GALLLTGGYEMDARISKLCERAFATGLPVFMVNTNTWQTSLSLQSFNLEVPVDDHERIEK

VQEYVANYINADWIESLTATSERSRRLSPPAFRYQLTELARKAGKRIVLPEGDEPRTVKA

AAICAERGIATCVLLGNPAEINRVAASQGVELGAGIEIVDPEVVRESYVGRLVELRKNKG

MTETVAREQLEDNVVLGTLMLEQDEVDGLVSGAVHTTANTIRPPLQLIKTAPGSSLVSSV

FFMLLPEQVYVYGDCAINPDPTAEQLAEIAIQSADSAAAFGIEPRVAMLSYSTGTSGAGS

DVEKVREATRLAQEKRPDLMIDGPLQYDAAVMADVAKSKAPNSPVAGRATVFIFPDLNTG

NTTYKAVQRSADLISIGPMLQGMRKPVNDLSRGALVDDIVYTIALTAIQSAQQQ

>sp|P38503|PTAS_METTE Phosphate acetyltransferase OS=Methanosarcina thermophila GN=pta PE=1 SV=3

MVTFLEKISERAKKLNKTIALPETEDIRTLQAAAKILERGIADIVLVGNEADIKALAGDL

DLSKAKIVDPKTYEKKDEYINAFYELRKHKGITLENAAEIMSDYVYFAVMMAKLGEVDGV

VSGAAHSSSDTLRPAVQIVKTAKGAALASAFFIISVPDCEYGSDGTFLFADSGMVEMPSV

EDVANIAVISAKTFELLVQDVPKVAMLSYSTKGSAKSKLTEATIASTKLAQELAPDIAID

GELQVDAAIVPKVAASKAPGSPVAGKANVFIFPDLNCGNIAYKIAQRLAKAEAYGPITQG

LAKPINDLSRGCSDEDIVGAVAITCVQAAAQDK

>sp|P39646|PTAS_BACSU Phosphate acetyltransferase OS=Bacillus subtilis (strain 168) GN=pta PE=1 SV=3

MADLFSTVQEKVAGKDVKIVFPEGLDERILEAVSKLAGNKVLNPIVIGNENEIQAKAKEL

NLTLGGVKIYDPHTYEGMEDLVQAFVERRKGKATEEQARKALLDENYFGTMLVYKGLADG

LVSGAAHSTADTVRPALQIIKTKEGVKKTSGVFIMARGEEQYVFADCAINIAPDSQDLAE

IAIESANTAKMFDIEPRVAMLSFSTKGSAKSDETEKVADAVKIAKEKAPELTLDGEFQFD

AAFVPSVAEKKAPDSEIKGDANVFVFPSLEAGNIGYKIAQRLGNFEAVGPILQGLNMPVN

DLSRGCNAEDVYNLALITAAQAL

>sp|Q8ZND6|PTA_SALTY Phosphate acetyltransferase OS=Salmonella typhimurium (strain LT2 / SGSC1412 / ATCC 700720) GN=pta PE=1 SV=1

MSRIIMLIPTGTSVGLTSVSLGVIRAMERKGVRLSVFKPIAQPRAGGDAPDQTTTIVRAN

STLPAAEPLKMSHVESLLSSNQKDVLMEEIIANYHANTKDAEVVLVEGLVPTRKHQFAQS

LNYEIAKTLNAEIVFVMSQGTDTPEQLNERIELTRSSFGGAKNTNITGVIINKLNAPVDE

QGRTRPDLSEIFDDSSKAQVIKIDPAKLQESSPLPVLGAVPWSFDLIATRAIDMARHLNA

TIINEGDIKTRRVKSVTFCARSIPHMLEHFRAGSLLVTSADRPDVLVAACLAAMNGVEIG

ALLLTGGYEMDARISKLCERAFATGLPVFMVNTNTWQTSLSLQSFNLEVPVDDHERIEKV

QEYVANYVNAEWIESLTATSERSRRLSPPAFRYQLTELARKAGKRVVLPEGDEPRTVKAA

AICAERGIATCVLLGNPDEINRVAASQGVELGAGIEIVDPEVVRESYVARLVELRKSKGM

TEPVAREQLEDNVVLGTLMLEQDEVDGLVSGAVHTTANTIRPPLQLIKTAPGSSLVSSVF

FMLLPEQVYVYGDCAINPDPTAEQLAEIAIQSADSAIAFGIEPRVAMLSYSTGTSGAGSD

VEKVREATRLAQEKRPDLMIDGPLQYDAAVMADVAKSKAPNSPVAGRATVFIFPDLNTGN

TTYKAVQRSADLISIGPMLQGMRKPVNDLSRGALVDDIVYTIALTAIQASQQQQ

>sp|P99092|PTAS_STAAN Phosphate acetyltransferase OS=Staphylococcus aureus (strain N315) GN=pta PE=1 SV=1

MADLLNVLKDKLSGKNVKIVLPEGEDERVLTAATQLQATDYVTPIVLGDETKVQSLAQKL

DLDISNIELINPATSELKAELVQSFVERRKGKATEEQAQELLNNVNYFGTMLVYAGKADG

LVSGAAHSTGDTVRPALQIIKTKPGVSRTSGIFFMIKGDEQYIFGDCAINPELDSQGLAE

IAVESAKSALSFGMDPKVAMLSFSTKGSAKSDDVTKVQEAVKLAQQKAEEEKLEAIIDGE

FQFDAAIVPGVAEKKAPGAKLQGDANVFVFPSLEAGNIGYKIAQRLGGYDAVGPVLQGLN

SPVNDLSRGCSIEDVYNLSIITAAQALQ

>sp|Q6GJ80|PTAS_STAAR Phosphate acetyltransferase OS=Staphylococcus aureus (strain MRSA252) GN=pta PE=1 SV=1

MADLLNVLKDKLSGKNVKIVLPEGEDERVLTAATQLQATDYVTPIVLGDETKVQSLAQKL

NLDISNIELINPATSELKAELVQSFVERRKGKTTEEQAQELLNNVNYFGTMLVYAGKADG

LVSGAAHSTGDTVRPALQIIKTKPGVSRTSGIFFMIKGDEQYIFGDCAINPELDSQGLAE

IAVESAKSALSFGMDPKVAMLSFSTKGSAKSDDVTKVQEAVKLAQQKAEEEKLEAIIDGE

FQFDAAIVPGVAEKKAPGAKLQGDANVFVFPSLEAGNIGYKIAQRLGGYDAVGPVLQGLN

SPVNDLSRGCSIEDVYNLSFITAAQALQ

>sp|Q9X0L4|PTAS_THEMA Phosphate acetyltransferase OS=Thermotoga maritima (strain ATCC 43589 / MSB8 / DSM 3109 / JCM 10099) GN=pta PE=1 SV=1

MFLEKLVEMARGKGKKLAVAAANDDHVIEAVYRAWRERVCEPVLFGPEEEITRIIEELVP

EWKNPQIIDCPPEEAGRLAVEAVSKGECDFLMKGKIKTGDLMKIYLDERYGLRTGKTMAM

VSVMEIPDFPRPLIISDPGMLISPTLEQKVDMIEHCVRVANVMGLETPKVAVVGAIEVVN

PKMPITMEAAILSKMNQRGQIKGCIVDGPFALDNVVSEEAAKKKGIQSPVAGKADILILP

DIEAANILYKALVFLAKAKSASTILGGKVPVVLTSRADSEETKFYSIALSAVFA

>sp|P9WHP1|PTA_MYCTU Phosphate acetyltransferase OS=Mycobacterium tuberculosis (strain ATCC 25618 / H37Rv) GN=pta PE=1 SV=1

MADSSAIYLAAPESQTGKSTIALGLLHRLTAMVAKVGVFRPITRLSAERDYILELLLAHT

SAGLPYERCVGVTYQQLHADRDDAIAEIVDSYHAMADECDAVVVVGSDYTDVTSPTELSV

NGRIAVNLGAPVLLTVRAKDRTPDQVASVVEVCLAELDTQRAHTAAVVANRCELSAIPAV

TDALRRFTPPSYVVPEEPLLSAPTVAELTQAVNGAVVSGDVALREREVMGVLAAGMTADH

VLERLTDGMAVITPGDRSDVVLAVASAHAAEGFPSLSCIVLNGGFQLHPAIAALVSGLRL

RLPVIATALGTYDTASAAASARGLVTATSQRKIDTALELMDRHVDVAGLLAQLTIPIPTV

TTPQMFTYRLLQQARSDLMRIVLPEGDDDRILKSAGRLLQRGIVDLTILGDEAKVRLRAA

ELGVDLDGATVIEPCASELHDQFADQYAQLRKAKGITVEHAREIMNDATYFGTMLVHNCH

ADGMVSGAAHTTAHTVRPALEIIKTVPGISTVSSIFLMCLPDRVLAYGDCAIIPNPTVEQ

LADIAICSARTAAQFGIEPRVAMLSYSTGDSGKGADVDKVRAATELVRAREPQLPVEGPI

QYDAAVEPSVAATKLRDSPVAGRATVLIFPDLNTGNNTYKAVQRSAGAIAIGPVLQGLRK

PVNDLSRGALVDDIVNTVAITAIQAQGVHE

>sp|Q9I5A5|PTA_PSEAE Phosphate acetyltransferase OS=Pseudomonas aeruginosa (strain ATCC 15692 / PAO1 / 1C / PRS 101 / LMG 12228) GN=pta PE=1 SV=1

MHTFFIAPTGFGVGLTSISLGLLRALERAGLKVGFFKPIAQLHPGDLGPERSSELVARTH

GLDTPKPLPLAQVERMLGDGQLDELLEEIISLYQRAAADKDVVIVEGMVPTRHASYAARV

NFHLAKSLDAEVILVSAPENETLTELTDRIEIQAQLFGGPRDPKVLGVILNKVRGEADAA

NAEDGVADFARRLTEHSPLLRDDFRLIGCIPWQDELNAARTRDIADLLSARVINAGDYEQ

RRVQKIVLCARAVPNTVQLLKPGVLVVTPGDRDDIILAASLAAMNGVPLAGLLLCSDFPP

DPRIMELCRGALQGGLPVLSVATGSYDTATNLNRMNKEIPVDDRERAERVTEFVAGHIDF

EWLKQRCGTPRELRLSPPAFRYQVVQRAQKAGKRIVLPEGSEPRTVQAAAICQARGIARC

VLLAKPEEVQAVAQAQGIVLPEGLEIIDPDLVRQRYVEPMVELRKGKGLNAPMAEQQLED

SVVLATMMLALDEVDGLVSGAIHTTASTIRPALQLIKTAPGYNLVSSVFFMLLPDQVLVY

GDCAVNPDPSASDLAEIAVQSAASAQAFGIPARVAMISYSTGDSGSGVDVDKVREATRLA

REQRPDLLIDGPLQYDAAAIASVGRQKAPNSPVAGQATVFIFPDLNTGNTTYKAVQRSAD

CVSVGPMLQGLRKPVNDLSRGALVEDIVYTIALTAIQADAQAPA

>sp|P0A6A3|ACKA_ECOLI Acetate kinase OS=Escherichia coli (strain K12) GN=ackA PE=1 SV=1

MSSKLVLVLNCGSSSLKFAIIDAVNGEEYLSGLAECFHLPEARIKWKMDGNKQEAALGAG

AAHSEALNFIVNTILAQKPELSAQLTAIGHRIVHGGEKYTSSVVIDESVIQGIKDAASFA

PLHNPAHLIGIEEALKSFPQLKDKNVAVFDTAFHQTMPEESYLYALPYNLYKEHGIRRYG

AHGTSHFYVTQEAAKMLNKPVEELNIITCHLGNGGSVSAIRNGKCVDTSMGLTPLEGLVM

GTRSGDIDPAIIFHLHDTLGMSVDAINKLLTKESGLLGLTEVTSDCRYVEDNYATKEDAK

RAMDVYCHRLAKYIGAYTALMDGRLDAVVFTGGIGENAAMVRELSLGKLGVLGFEVDHER

NLAARFGKSGFINKEGTRPAVVIPTNEELVIAQDASRLTA

>sp|P38502|ACKA_METTE Acetate kinase OS=Methanosarcina thermophila GN=ackA PE=1 SV=1

MKVLVINAGSSSLKYQLIDMTNESALAVGLCERIGIDNSIITQKKFDGKKLEKLTDLPTH

KDALEEVVKALTDDEFGVIKDMGEINAVGHRVVHGGEKFTTSALYDEGVEKAIKDCFELA

PLHNPPNMMGISACAEIMPGTPMVIVFDTAFHQTMPPYAYMYALPYDLYEKHGVRKYGFH

GTSHKYVAERAALMLGKPAEETKIITCHLGNGSSITAVEGGKSVETSMGFTPLEGLAMGT

RCGSIDPAIVPFLMEKEGLTTREIDTLMNKKSGVLGVSGLSNDFRDLDEAASKGNRKAEL

ALEIFAYKVKKFIGEYSAVLNGADAVVFTAGIGENSASIRKRILTGLDGIGIKIDDEKNK

IRGQEIDISTPDAKVRVFVIPTNEELAIARETKEIVETEVKLRSSIPV

>sp|P37877|ACKA_BACSU Acetate kinase OS=Bacillus subtilis (strain 168) GN=ackA PE=1 SV=1

MSKIIAINAGSSSLKFQLFEMPSETVLTKGLVERIGIADSVFTISVNGEKNTEVTDIPDH

AVAVKMLLNKLTEFGIIKDLNEIDGIGHRVVHGGEKFSDSVLLTDETIKEIEDISELAPL

HNPANIVGIKAFKEVLPNVPAVAVFDTAFHQTMPEQSYLYSLPYEYYEKFGIRKYGFHGT

SHKYVTERAAELLGRPLKDLRLISCHLGNGASIAAVEGGKSIDTSMGFTPLAGVAMGTRS

GNIDPALIPYIMEKTGQTADEVLNTLNKKSGLLGISGFSSDLRDIVEATKEGNERAETAL

EVFASRIHKYIGSYAARMSGVDAIIFTAGIGENSVEVRERVLRGLEFMGVYWDPALNNVR

GEEAFISYPHSPVKVMIIPTDEEVMIARDVVRLAK

>sp|A0QQK1|ACKA_MYCS2 Acetate kinase OS=Mycobacterium smegmatis (strain ATCC 700084 / mc(2)155) GN=ackA PE=1 SV=1

MTVLVVNSGSSSLKYAVVRPASGEFLADGIIEEIGSGAVPDHDAALRAAFDELAAAGLHL

EDLDLKAVGHRMVHGGKTFYKPSVVDDELIAKARELSPLAPLHNPPAIKGIEVARKLLPD

LPHIAVFDTAFFHDLPAPASTYAIDRELAETWHIKRYGFHGTSHEYVSQQAAIFLDRPLE

SLNQIVLHLGNGASASAVAGGKAVDTSMGLTPMEGLVMGTRSGDIDPGVIMYLWRTAGMS

VDDIESMLNRRSGVLGLGGASDFRKLRELIESGDEHAKLAYDVYIHRLRKYIGAYMAVLG

RTDVISFTAGVGENVPPVRRDALAGLGGLGIEIDDALNSAKSDEPRLISTPDSRVTVLVV

PTNEELAIARACVGVV

>sp|P63411|ACKA_SALTY Acetate kinase OS=Salmonella typhimurium (strain LT2 / SGSC1412 / ATCC 700720) GN=ackA PE=1 SV=1

MSSKLVLVLNCGSSSLKFAIIDAVNGDEYLSGLAECFHLPEARIKWKMDGSKQEAALGAG

AAHSEALNFIVNTILAQKPELSAQLTAIGHRIVHGGEKYTSSVVIDESVIQGIKDSASFA

PLHNPAHLIGIAEALKSFPQLKDKNVAVFDTAFHQTMPEESYLYALPYSLYKEHGVRRYG

AHGTSHFYVTQEAAKMLNKPVEELNIITCHLGNGGSVSAIRNGKCVDTSMGLTPLEGLVM

GTRSGDIDPAIIFHLHDTLGMSVDQINKMLTKESGLLGLTEVTSDCRYVEDNYATKEDAK

RAMDVYCHRLAKYIGSYTALMDGRLDAVVFTGGIGENAAMVRELSLGKLGVLGFEVDHER

NLAARFGKSGFINKEGTRPAVVIPTNEELVIAQDASRLTA

>sp|Q9WYB1|ACKA_THEMA Acetate kinase OS=Thermotoga maritima (strain ATCC 43589 / MSB8 / DSM 3109 / JCM 10099) GN=ackA PE=1 SV=1

MRVLVINSGSSSIKYQLIEMEGEKVLCKGIAERIGIEGSRLVHRVGDEKHVIERELPDHE

EALKLILNTLVDEKLGVIKDLKEIDAVGHRVVHGGERFKESVLVDEEVLKAIEEVSPLAP

LHNPANLMGIKAAMKLLPGVPNVAVFDTAFHQTIPQKAYLYAIPYEYYEKYKIRRYGFHG

TSHRYVSKRAAEILGKKLEELKIITCHIGNGASVAAVKYGKCVDTSMGFTPLEGLVMGTR

SGDLDPAIPFFIMEKEGISPQEMYDILNKKSGVYGLSKGFSSDMRDIEEAALKGDEWCKL

VLEIYDYRIAKYIGAYAAAMNGVDAIVFTAGVGENSPITREDVCSYLEFLGVKLDKQKNE

ETIRGKEGIISTPDSRVKVLVVPTNEELMIARDTKEIVEKIGR

>sp|A0QLU8|ACKA_MYCA1 Acetate kinase OS=Mycobacterium avium (strain 104) GN=ackA PE=1 SV=1

MDGSDGARRVLVINSGSSSLKFQLVDPESGVAASTGIVERIGEESSPVPDHDAALRRAFD

MLAGDGVDLNTAGLVAVGHRVVHGGNTFYRPTVLDDAVIARLHELSELAPLHNPPALLGI

EVARRLLPGIAHVAVFDTGFFHDLPPAAATYAIDRELADRWQIRRYGFHGTSHRYVSEQA

AAFLDRPLRGLKQIVLHLGNGCSASAIAGTRPLDTSMGLTPLEGLVMGTRSGDIDPSVVS

YLCHTAGMGVDDVESMLNHRSGVVGLSGVRDFRRLRELIESGDGAAQLAYSVFTHRLRKY

IGAYLAVLGHTDVISFTAGIGENDAAVRRDAVSGMEELGIVLDERRNLPGAKGARQISAD

DSPITVLVVPTNEELAIARDCVRVLGG

>sp|B2HPZ3|ACKA_MYCMM Acetate kinase OS=Mycobacterium marinum (strain ATCC BAA-535 / M) GN=ackA PE=1 SV=1

MSASRPNRVVLVLNSGSSSLKFQLVEPDSGMSRATGNIERIGEESSSVPDHDAALRRVFE

ILAEDDIDLQSCGLVAVGHRVVHGGKDFYEPTLLNDAVIGKLDELSPLAPLHNPPAVLCI

RVARALLPDVPHIAVFDTAFFHQLPPAAATYAIDRELADVWKIRRYGFHGTSHEYVSQQA

AEFLGKPIGDLNQIVLHLGNGASASAVAGGRPVETSMGLTPLEGLVMGTRSGDLDPGVIG

YLWRTAKLGVDEIESMLNHRSGMLGLAGERDFRRLRAMIDDGDPAAELAYDVFIHRLRKY

VGAYLAVLGHTDVVSFTAGIGEHDAAVRRDTLAGMAELGISLDERRNACPSGGARRISAD

DSPVTVLVIPTNEELAIARHCCSVLVAV

>sp|Q73T33|ACKA_MYCPA Acetate kinase OS=Mycobacterium paratuberculosis (strain ATCC BAA-968 / K-10) GN=ackA PE=1 SV=1

MDGSDGARRVLVINSGSSSLKFQLVDPEFGVAASTGIVERIGEESSPVPDHDAALRRAFD

MLAGDGVDLNTAGLVAVGHRVVHGGNTFYRPTVLDDAVIARLHELSELAPLHNPPALQGI

EVARRLLPDIAHVAVFDTGFFHDLPPAAATYAIDRELADRWQIRRYGFHGTSHRYVSEQA

AAFLDRPLRGLKQIVLHLGNGCSASAIAGTRPLDTSMGLTPLEGLVMGTRSGDIDPSIVS

YLCHTAGMGVDDVESMLNHRSGVVGLSGVRDFRRLRELIESGDGAAQLAYSVFTHRLRKY

IGAYLAVLGHTDVISFTAGIGENDAAVRRDAVSGMEELGIVLDERRNLAGGKGARQISAD

DSPITVLVVPTNEELAIARDCVRVLGG

>sp|P75245|ACKA_MYCPN Acetate kinase OS=Mycoplasma pneumoniae (strain ATCC 29342 / M129) GN=ackA PE=1 SV=1

MNDNKILVVNAGSSSIKFQLFDYHKKVLAKALCERIFVDGFFKLEFNEQKVEEKVAFPDH

HAAVTHFLNTLKKHKIIQELSDIILVGHRVVQGANYFKDSVIVDAEALAKIKEFIKLAPL

HNKPEADVIEIFFKEVPSAKNVAVFDTTFHTTIPQENYLYAVPRSWEQKHLVRRYGFHGT

SYKFINNYLEKHLNKQNLNLIVCHLGNGASVCAIKNGKSFNTSMGFTPLEGLIMGTRSGD

LDPAIIGYVAEQENMSASDVVNALNKKSGMLALTGASDMRDVFAKPQENAVAIKMYVNRV

ADYIAKYLNQLEGNIDGLVFTGGIGENASDCVELFINAVKSLGFATDLKLFVKYGDSCVV

STPQSKYKIYRVRTNEELMIVEDSIRLTQK

>sp|P9WQH1|ACKA_MYCTU Acetate kinase OS=Mycobacterium tuberculosis (strain ATCC 25618 / H37Rv) GN=ackA PE=1 SV=1

MSSTVLVINSGSSSLKFQLVEPVAGMSRAAGIVERIGERSSPVADHAQALHRAFKMLAED

GIDLQTCGLVAVGHRVVHGGTEFHQPTLLDDTVIGKLEELSALAPLHNPPAVLGIKVARR

LLANVAHVAVFDTAFFHDLPPAAATYAIDRDVADRWHIRRYGFHGTSHQYVSERAAAFLG

RPLDGLNQIVLHLGNGASASAIARGRPVETSMGLTPLEGLVMGTRSGDLDPGVISYLWRT

ARMGVEDIESMLNHRSGMLGLAGERDFRRLRLVIETGDRSAQLAYEVFIHRLRKYLGAYL

AVLGHTDVVSFTAGIGENDAAVRRDALAGLQGLGIALDQDRNLGPGHGARRISSDDSPIA

VLVVPTNEELAIARDCLRVLGGRRA

>sp|Q99TF2|ACKA_STAAN Acetate kinase OS=Staphylococcus aureus (strain N315) GN=ackA PE=1 SV=1

MSKLILAINAGSSSLKFQLIRMPEEELVTKGLIERIGLKDSIFTIEVNGEKVKTVQDIKD

HVEAVDIMLDAFKAHNIINDINDIDGTGHRVVHGGEKFPESVAITDEVEKEIEELSELAP

LHNPANLMGIRAFRKLLPNIPHVAIFDTAFHQTMPEKAYLYSLPYHYYKDYGIRKYGFHG

TSHKFVSQRAAEMLDKPIEDLRIISCHIGNGASIAAIDGGKSIDTSMGFTPLAGVTMGTR

SGNIDPALIPFIMEKTGKTAEQVLEILNKESGLLGLSGTSSDLRDLSEEAESGKARSQMA

LDVFASKIHKYIGSYAARMHGVDVIVFTAGIGENSVEIRAKVLEGLEFMGVYWDPKKNEN

LLRGKEGFINYPHSPVKVVVIPTDEESMIARDVMTFGGLK

*3. Acetate formation pathway IV.*

>sp|P09373|PFLB_ECOLI Formate acetyltransferase 1 OS=Escherichia coli (strain K12) GN=pflB PE=1 SV=2

MSELNEKLATAWEGFTKGDWQNEVNVRDFIQKNYTPYEGDESFLAGATEATTTLWDKVME

GVKLENRTHAPVDFDTAVASTITSHDAGYINKQLEKIVGLQTEAPLKRALIPFGGIKMIE

GSCKAYNRELDPMIKKIFTEYRKTHNQGVFDVYTPDILRCRKSGVLTGLPDAYGRGRIIG

DYRRVALYGIDYLMKDKLAQFTSLQADLENGVNLEQTIRLREEIAEQHRALGQMKEMAAK

YGYDISGPATNAQEAIQWTYFGYLAAVKSQNGAAMSFGRTSTFLDVYIERDLKAGKITEQ

EAQEMVDHLVMKLRMVRFLRTPEYDELFSGDPIWATESIGGMGLDGRTLVTKNSFRFLNT

LYTMGPSPEPNMTILWSEKLPLNFKKFAAKVSIDTSSLQYENDDLMRPDFNNDDYAIACC

VSPMIVGKQMQFFGARANLAKTMLYAINGGVDEKLKMQVGPKSEPIKGDVLNYDEVMERM

DHFMDWLAKQYITALNIIHYMHDKYSYEASLMALHDRDVIRTMACGIAGLSVAADSLSAI

KYAKVKPIRDEDGLAIDFEIEGEYPQFGNNDPRVDDLAVDLVERFMKKIQKLHTYRDAIP

TQSVLTITSNVVYGKKTGNTPDGRRAGAPFGPGANPMHGRDQKGAVASLTSVAKLPFAYA

KDGISYTFSIVPNALGKDDEVRKTNLAGLMDGYFHHEASIEGGQHLNVNVMNREMLLDAM

ENPEKYPQLTIRVSGYAVRFNSLTKEQQQDVITRTFTQSM

>sp|P42632|TDCE_ECOLI PFL-like enzyme TdcE OS=Escherichia coli (strain K12) GN=tdcE PE=1 SV=2

MKVDIDTSDKLYADAWLGFKGTDWKNEINVRDFIQHNYTPYEGDESFLAEATPATTELWE

KVMEGIRIENATHAPVDFDTNIATTITAHDAGYINQPLEKIVGLQTDAPLKRALHPFGGI

NMIKSSFHAYGREMDSEFEYLFTDLRKTHNQGVFDVYSPDMLRCRKSGVLTGLPDGYGRG

RIIGDYRRVALYGISYLVRERELQFADLQSRLEKGEDLEATIRLREELAEHRHALLQIQE

MAAKYGFDISRPAQNAQEAVQWLYFAYLAAVKSQNGGAMSLGRTASFLDIYIERDFKAGV

LNEQQAQELIDHFIMKIRMVRFLRTPEFDSLFSGDPIWATEVIGGMGLDGRTLVTKNSFR

YLHTLHTMGPAPEPNLTILWSEELPIAFKKYAAQVSIVTSSLQYENDDLMRTDFNSDDYA

IACCVSPMVIGKQMQFFGARANLAKTLLYAINGGVDEKLKIQVGPKTAPLMDDVLDYDKV

MDSLDHFMDWLAVQYISALNIIHYMHDKYSYEASLMALHDRDVYRTMACGIAGLSVATDS

LSAIKYARVKPIRDENGLAVDFEIDGEYPQYGNNDERVDSIACDLVERFMKKIKALPTYR

NAVPTQSILTITSNVVYGQKTGNTPDGRRAGTPFAPGANPMHGRDRKGAVASLTSVAKLP

FTYAKDGISYTFSIVPAALGKEDPVRKTNLVGLLDGYFHHEADVEGGQHLNVNVMNREML

LDAIEHPEKYPNLTIRVSGYAVRFNALTREQQQDVISRTFTQAL

>sp|Q5HJF4|PFLB_STAAC Formate acetyltransferase OS=Staphylococcus aureus (strain COL) GN=pflB PE=1 SV=1

MLETNKNHATAWQGFKNGRWNRHVDVREFIQLNYTLYEGNDSFLAGPTEATSKLWEQVMQ

LSKEERERGGMWDMDTKVASTITSHDAGYLDKDLETIVGVQTEKPFKRSMQPFGGIRMAK

AACEAYGYELDEETEKIFTDYRKTHNQGVFDAYSREMLNCRKAGVITGLPDAYGRGRIIG

DYRRVALYGVDFLMEEKMHDFNTMSTEMSEDVIRLREELSEQYRALKELKELGQKYGFDL

SRPAENFKEAVQWLYLAYLAAIKEQNGAAMSLGRTSTFLDIYAERDLKAGVITESEVQEI

IDHFIMKLRIVKFARTPDYNELFSGDPTWVTESIGGVGIDGRPLVTKNSFRFLHSLDNLG

PAPEPNLTVLWSVRLPDNFKTYCAKMSIKTSSIQYENDDIMRESYGDDYGIACCVSAMTI

GKQMQFFGARANLAKTLLYAINGGKDEKSGAQVGPNFEGINSEVLEYDEVFKKFDQMMDW

LAGVYINSLNVIHYMHDKYSYERIEMALHDTEIVRTMATGIAGLSVAADSLSAIKYAQVK

PIRNEEGLVVDFEIEGDFPKYGNNDDRVDDIAVDLVERFMTKLRSHKTYRDSEHTMSVLT

ITSNVVYGKKTGNTPDGRKAGEPFAPGANPMHGRDQKGALSSLSSVAKIPYDCCKDGISN

TFSIVPKSLGKEPEDQNRNLTSMLDGYAMQCGHHLNINVFNRETLIDAMEHPEEYPQLTI

RVSGYAVNFIKLTREQQLDVISRTFHESM

>sp|Q7A7X6|PFLB_STAAN Formate acetyltransferase OS=Staphylococcus aureus (strain N315) GN=pflB PE=1 SV=1

MLETNKNHATAWQGFKNGRWNRHVDVREFIQLNYTLYEGNDSFLAGPTEATSKLWEQVMQ

LSKEERERGGMWDMDTKVASTITSHDAGYLDKDLETIVGVQTEKPFKRSMQPFGGIRMAK

AACEAYGYELDEETEKIFTDYRKTHNQGVFDAYSREMLNCRKAGVITGLPDAYGRGRIIG

DYRRVALYGVDFLMEEKMHDFNTMSTEMSEDVIRLREELSEQYRALKELKELGQKYGFDL

SRPAENFKEAVQWLYLAYLAAIKEQNGAAMSLGRTSTFLDIYAERDLKAGVITESEVQEI

IDHFIMKLRIVKFARTPDYNELFSGDPTWVTESIGGVGIDGRPLVTKNSFRFLHSLDNLG

PAPEPNLTVLWSVRLPDNFKTYCAKMSIKTSSIQYENDDIMRESYGDDYGIACCVSAMTI

GKQMQFFGARANLAKTLLYAINGGKDEKSGAQVGPNFEGINSEVLEYDEVFKKFDQMMDW

LAGVYINSLNVIHYMHDKYSYERIEMALHDTEIVRTMATGIAGLSVAADSLSAIKYAQVK

PIRNEEGLVVDFEIEGDFPKYGNNDDRVDDIAVDLVERFMTKLRSHKTYRDSEHTMSVLT

ITSNVVYGKKTGNTPDGRKAGEPFAPGANPMHGRDQKGALSSLSSVAKIPYDCCKDGISN

TFSIVPKSLGKEPEDQNRNLTSMLDGYAMQCGHHLNINVFNRETLIDAMEHPEEYPQLTI

RVSGYAVNFIKLTREQQLDVISRTFHESM

>sp|P0A9M8|PTA_ECOLI Phosphate acetyltransferase OS=Escherichia coli (strain K12) GN=pta PE=1 SV=2

MSRIIMLIPTGTSVGLTSVSLGVIRAMERKGVRLSVFKPIAQPRTGGDAPDQTTTIVRAN

SSTTTAAEPLKMSYVEGLLSSNQKDVLMEEIVANYHANTKDAEVVLVEGLVPTRKHQFAQ

SLNYEIAKTLNAEIVFVMSQGTDTPEQLKERIELTRNSFGGAKNTNITGVIVNKLNAPVD

EQGRTRPDLSEIFDDSSKAKVNNVDPAKLQESSPLPVLGAVPWSFDLIATRAIDMARHLN

ATIINEGDINTRRVKSVTFCARSIPHMLEHFRAGSLLVTSADRPDVLVAACLAAMNGVEI

GALLLTGGYEMDARISKLCERAFATGLPVFMVNTNTWQTSLSLQSFNLEVPVDDHERIEK

VQEYVANYINADWIESLTATSERSRRLSPPAFRYQLTELARKAGKRIVLPEGDEPRTVKA

AAICAERGIATCVLLGNPAEINRVAASQGVELGAGIEIVDPEVVRESYVGRLVELRKNKG

MTETVAREQLEDNVVLGTLMLEQDEVDGLVSGAVHTTANTIRPPLQLIKTAPGSSLVSSV

FFMLLPEQVYVYGDCAINPDPTAEQLAEIAIQSADSAAAFGIEPRVAMLSYSTGTSGAGS

DVEKVREATRLAQEKRPDLMIDGPLQYDAAVMADVAKSKAPNSPVAGRATVFIFPDLNTG

NTTYKAVQRSADLISIGPMLQGMRKPVNDLSRGALVDDIVYTIALTAIQSAQQQ

>sp|P38503|PTAS_METTE Phosphate acetyltransferase OS=Methanosarcina thermophila GN=pta PE=1 SV=3

MVTFLEKISERAKKLNKTIALPETEDIRTLQAAAKILERGIADIVLVGNEADIKALAGDL

DLSKAKIVDPKTYEKKDEYINAFYELRKHKGITLENAAEIMSDYVYFAVMMAKLGEVDGV

VSGAAHSSSDTLRPAVQIVKTAKGAALASAFFIISVPDCEYGSDGTFLFADSGMVEMPSV

EDVANIAVISAKTFELLVQDVPKVAMLSYSTKGSAKSKLTEATIASTKLAQELAPDIAID

GELQVDAAIVPKVAASKAPGSPVAGKANVFIFPDLNCGNIAYKIAQRLAKAEAYGPITQG

LAKPINDLSRGCSDEDIVGAVAITCVQAAAQDK

>sp|P39646|PTAS_BACSU Phosphate acetyltransferase OS=Bacillus subtilis (strain 168) GN=pta PE=1 SV=3

MADLFSTVQEKVAGKDVKIVFPEGLDERILEAVSKLAGNKVLNPIVIGNENEIQAKAKEL

NLTLGGVKIYDPHTYEGMEDLVQAFVERRKGKATEEQARKALLDENYFGTMLVYKGLADG

LVSGAAHSTADTVRPALQIIKTKEGVKKTSGVFIMARGEEQYVFADCAINIAPDSQDLAE

IAIESANTAKMFDIEPRVAMLSFSTKGSAKSDETEKVADAVKIAKEKAPELTLDGEFQFD

AAFVPSVAEKKAPDSEIKGDANVFVFPSLEAGNIGYKIAQRLGNFEAVGPILQGLNMPVN

DLSRGCNAEDVYNLALITAAQAL

>sp|Q8ZND6|PTA_SALTY Phosphate acetyltransferase OS=Salmonella typhimurium (strain LT2 / SGSC1412 / ATCC 700720) GN=pta PE=1 SV=1

MSRIIMLIPTGTSVGLTSVSLGVIRAMERKGVRLSVFKPIAQPRAGGDAPDQTTTIVRAN

STLPAAEPLKMSHVESLLSSNQKDVLMEEIIANYHANTKDAEVVLVEGLVPTRKHQFAQS

LNYEIAKTLNAEIVFVMSQGTDTPEQLNERIELTRSSFGGAKNTNITGVIINKLNAPVDE

QGRTRPDLSEIFDDSSKAQVIKIDPAKLQESSPLPVLGAVPWSFDLIATRAIDMARHLNA

TIINEGDIKTRRVKSVTFCARSIPHMLEHFRAGSLLVTSADRPDVLVAACLAAMNGVEIG

ALLLTGGYEMDARISKLCERAFATGLPVFMVNTNTWQTSLSLQSFNLEVPVDDHERIEKV

QEYVANYVNAEWIESLTATSERSRRLSPPAFRYQLTELARKAGKRVVLPEGDEPRTVKAA

AICAERGIATCVLLGNPDEINRVAASQGVELGAGIEIVDPEVVRESYVARLVELRKSKGM

TEPVAREQLEDNVVLGTLMLEQDEVDGLVSGAVHTTANTIRPPLQLIKTAPGSSLVSSVF

FMLLPEQVYVYGDCAINPDPTAEQLAEIAIQSADSAIAFGIEPRVAMLSYSTGTSGAGSD

VEKVREATRLAQEKRPDLMIDGPLQYDAAVMADVAKSKAPNSPVAGRATVFIFPDLNTGN

TTYKAVQRSADLISIGPMLQGMRKPVNDLSRGALVDDIVYTIALTAIQASQQQQ

>sp|P99092|PTAS_STAAN Phosphate acetyltransferase OS=Staphylococcus aureus (strain N315) GN=pta PE=1 SV=1

MADLLNVLKDKLSGKNVKIVLPEGEDERVLTAATQLQATDYVTPIVLGDETKVQSLAQKL

DLDISNIELINPATSELKAELVQSFVERRKGKATEEQAQELLNNVNYFGTMLVYAGKADG

LVSGAAHSTGDTVRPALQIIKTKPGVSRTSGIFFMIKGDEQYIFGDCAINPELDSQGLAE

IAVESAKSALSFGMDPKVAMLSFSTKGSAKSDDVTKVQEAVKLAQQKAEEEKLEAIIDGE

FQFDAAIVPGVAEKKAPGAKLQGDANVFVFPSLEAGNIGYKIAQRLGGYDAVGPVLQGLN

SPVNDLSRGCSIEDVYNLSIITAAQALQ

>sp|Q6GJ80|PTAS_STAAR Phosphate acetyltransferase OS=Staphylococcus aureus (strain MRSA252) GN=pta PE=1 SV=1

MADLLNVLKDKLSGKNVKIVLPEGEDERVLTAATQLQATDYVTPIVLGDETKVQSLAQKL

NLDISNIELINPATSELKAELVQSFVERRKGKTTEEQAQELLNNVNYFGTMLVYAGKADG

LVSGAAHSTGDTVRPALQIIKTKPGVSRTSGIFFMIKGDEQYIFGDCAINPELDSQGLAE

IAVESAKSALSFGMDPKVAMLSFSTKGSAKSDDVTKVQEAVKLAQQKAEEEKLEAIIDGE

FQFDAAIVPGVAEKKAPGAKLQGDANVFVFPSLEAGNIGYKIAQRLGGYDAVGPVLQGLN

SPVNDLSRGCSIEDVYNLSFITAAQALQ

>sp|Q9X0L4|PTAS_THEMA Phosphate acetyltransferase OS=Thermotoga maritima (strain ATCC 43589 / MSB8 / DSM 3109 / JCM 10099) GN=pta PE=1 SV=1

MFLEKLVEMARGKGKKLAVAAANDDHVIEAVYRAWRERVCEPVLFGPEEEITRIIEELVP

EWKNPQIIDCPPEEAGRLAVEAVSKGECDFLMKGKIKTGDLMKIYLDERYGLRTGKTMAM

VSVMEIPDFPRPLIISDPGMLISPTLEQKVDMIEHCVRVANVMGLETPKVAVVGAIEVVN

PKMPITMEAAILSKMNQRGQIKGCIVDGPFALDNVVSEEAAKKKGIQSPVAGKADILILP

DIEAANILYKALVFLAKAKSASTILGGKVPVVLTSRADSEETKFYSIALSAVFA

>sp|P9WHP1|PTA_MYCTU Phosphate acetyltransferase OS=Mycobacterium tuberculosis (strain ATCC 25618 / H37Rv) GN=pta PE=1 SV=1

MADSSAIYLAAPESQTGKSTIALGLLHRLTAMVAKVGVFRPITRLSAERDYILELLLAHT

SAGLPYERCVGVTYQQLHADRDDAIAEIVDSYHAMADECDAVVVVGSDYTDVTSPTELSV

NGRIAVNLGAPVLLTVRAKDRTPDQVASVVEVCLAELDTQRAHTAAVVANRCELSAIPAV

TDALRRFTPPSYVVPEEPLLSAPTVAELTQAVNGAVVSGDVALREREVMGVLAAGMTADH

VLERLTDGMAVITPGDRSDVVLAVASAHAAEGFPSLSCIVLNGGFQLHPAIAALVSGLRL

RLPVIATALGTYDTASAAASARGLVTATSQRKIDTALELMDRHVDVAGLLAQLTIPIPTV

TTPQMFTYRLLQQARSDLMRIVLPEGDDDRILKSAGRLLQRGIVDLTILGDEAKVRLRAA

ELGVDLDGATVIEPCASELHDQFADQYAQLRKAKGITVEHAREIMNDATYFGTMLVHNCH

ADGMVSGAAHTTAHTVRPALEIIKTVPGISTVSSIFLMCLPDRVLAYGDCAIIPNPTVEQ

LADIAICSARTAAQFGIEPRVAMLSYSTGDSGKGADVDKVRAATELVRAREPQLPVEGPI

QYDAAVEPSVAATKLRDSPVAGRATVLIFPDLNTGNNTYKAVQRSAGAIAIGPVLQGLRK

PVNDLSRGALVDDIVNTVAITAIQAQGVHE

>sp|Q9I5A5|PTA_PSEAE Phosphate acetyltransferase OS=Pseudomonas aeruginosa (strain ATCC 15692 / PAO1 / 1C / PRS 101 / LMG 12228) GN=pta PE=1 SV=1

MHTFFIAPTGFGVGLTSISLGLLRALERAGLKVGFFKPIAQLHPGDLGPERSSELVARTH

GLDTPKPLPLAQVERMLGDGQLDELLEEIISLYQRAAADKDVVIVEGMVPTRHASYAARV

NFHLAKSLDAEVILVSAPENETLTELTDRIEIQAQLFGGPRDPKVLGVILNKVRGEADAA

NAEDGVADFARRLTEHSPLLRDDFRLIGCIPWQDELNAARTRDIADLLSARVINAGDYEQ

RRVQKIVLCARAVPNTVQLLKPGVLVVTPGDRDDIILAASLAAMNGVPLAGLLLCSDFPP

DPRIMELCRGALQGGLPVLSVATGSYDTATNLNRMNKEIPVDDRERAERVTEFVAGHIDF

EWLKQRCGTPRELRLSPPAFRYQVVQRAQKAGKRIVLPEGSEPRTVQAAAICQARGIARC

VLLAKPEEVQAVAQAQGIVLPEGLEIIDPDLVRQRYVEPMVELRKGKGLNAPMAEQQLED

SVVLATMMLALDEVDGLVSGAIHTTASTIRPALQLIKTAPGYNLVSSVFFMLLPDQVLVY

GDCAVNPDPSASDLAEIAVQSAASAQAFGIPARVAMISYSTGDSGSGVDVDKVREATRLA

REQRPDLLIDGPLQYDAAAIASVGRQKAPNSPVAGQATVFIFPDLNTGNTTYKAVQRSAD

CVSVGPMLQGLRKPVNDLSRGALVEDIVYTIALTAIQADAQAPA

>sp|P0A6A3|ACKA_ECOLI Acetate kinase OS=Escherichia coli (strain K12) GN=ackA PE=1 SV=1

MSSKLVLVLNCGSSSLKFAIIDAVNGEEYLSGLAECFHLPEARIKWKMDGNKQEAALGAG

AAHSEALNFIVNTILAQKPELSAQLTAIGHRIVHGGEKYTSSVVIDESVIQGIKDAASFA

PLHNPAHLIGIEEALKSFPQLKDKNVAVFDTAFHQTMPEESYLYALPYNLYKEHGIRRYG

AHGTSHFYVTQEAAKMLNKPVEELNIITCHLGNGGSVSAIRNGKCVDTSMGLTPLEGLVM

GTRSGDIDPAIIFHLHDTLGMSVDAINKLLTKESGLLGLTEVTSDCRYVEDNYATKEDAK

RAMDVYCHRLAKYIGAYTALMDGRLDAVVFTGGIGENAAMVRELSLGKLGVLGFEVDHER

NLAARFGKSGFINKEGTRPAVVIPTNEELVIAQDASRLTA

>sp|P38502|ACKA_METTE Acetate kinase OS=Methanosarcina thermophila GN=ackA PE=1 SV=1

MKVLVINAGSSSLKYQLIDMTNESALAVGLCERIGIDNSIITQKKFDGKKLEKLTDLPTH

KDALEEVVKALTDDEFGVIKDMGEINAVGHRVVHGGEKFTTSALYDEGVEKAIKDCFELA

PLHNPPNMMGISACAEIMPGTPMVIVFDTAFHQTMPPYAYMYALPYDLYEKHGVRKYGFH

GTSHKYVAERAALMLGKPAEETKIITCHLGNGSSITAVEGGKSVETSMGFTPLEGLAMGT

RCGSIDPAIVPFLMEKEGLTTREIDTLMNKKSGVLGVSGLSNDFRDLDEAASKGNRKAEL

ALEIFAYKVKKFIGEYSAVLNGADAVVFTAGIGENSASIRKRILTGLDGIGIKIDDEKNK

IRGQEIDISTPDAKVRVFVIPTNEELAIARETKEIVETEVKLRSSIPV

>sp|P37877|ACKA_BACSU Acetate kinase OS=Bacillus subtilis (strain 168) GN=ackA PE=1 SV=1

MSKIIAINAGSSSLKFQLFEMPSETVLTKGLVERIGIADSVFTISVNGEKNTEVTDIPDH

AVAVKMLLNKLTEFGIIKDLNEIDGIGHRVVHGGEKFSDSVLLTDETIKEIEDISELAPL

HNPANIVGIKAFKEVLPNVPAVAVFDTAFHQTMPEQSYLYSLPYEYYEKFGIRKYGFHGT

SHKYVTERAAELLGRPLKDLRLISCHLGNGASIAAVEGGKSIDTSMGFTPLAGVAMGTRS

GNIDPALIPYIMEKTGQTADEVLNTLNKKSGLLGISGFSSDLRDIVEATKEGNERAETAL

EVFASRIHKYIGSYAARMSGVDAIIFTAGIGENSVEVRERVLRGLEFMGVYWDPALNNVR

GEEAFISYPHSPVKVMIIPTDEEVMIARDVVRLAK

>sp|A0QQK1|ACKA_MYCS2 Acetate kinase OS=Mycobacterium smegmatis (strain ATCC 700084 / mc(2)155) GN=ackA PE=1 SV=1

MTVLVVNSGSSSLKYAVVRPASGEFLADGIIEEIGSGAVPDHDAALRAAFDELAAAGLHL

EDLDLKAVGHRMVHGGKTFYKPSVVDDELIAKARELSPLAPLHNPPAIKGIEVARKLLPD

LPHIAVFDTAFFHDLPAPASTYAIDRELAETWHIKRYGFHGTSHEYVSQQAAIFLDRPLE

SLNQIVLHLGNGASASAVAGGKAVDTSMGLTPMEGLVMGTRSGDIDPGVIMYLWRTAGMS

VDDIESMLNRRSGVLGLGGASDFRKLRELIESGDEHAKLAYDVYIHRLRKYIGAYMAVLG

RTDVISFTAGVGENVPPVRRDALAGLGGLGIEIDDALNSAKSDEPRLISTPDSRVTVLVV

PTNEELAIARACVGVV

>sp|P63411|ACKA_SALTY Acetate kinase OS=Salmonella typhimurium (strain LT2 / SGSC1412 / ATCC 700720) GN=ackA PE=1 SV=1

MSSKLVLVLNCGSSSLKFAIIDAVNGDEYLSGLAECFHLPEARIKWKMDGSKQEAALGAG

AAHSEALNFIVNTILAQKPELSAQLTAIGHRIVHGGEKYTSSVVIDESVIQGIKDSASFA

PLHNPAHLIGIAEALKSFPQLKDKNVAVFDTAFHQTMPEESYLYALPYSLYKEHGVRRYG

AHGTSHFYVTQEAAKMLNKPVEELNIITCHLGNGGSVSAIRNGKCVDTSMGLTPLEGLVM

GTRSGDIDPAIIFHLHDTLGMSVDQINKMLTKESGLLGLTEVTSDCRYVEDNYATKEDAK

RAMDVYCHRLAKYIGSYTALMDGRLDAVVFTGGIGENAAMVRELSLGKLGVLGFEVDHER

NLAARFGKSGFINKEGTRPAVVIPTNEELVIAQDASRLTA

>sp|Q9WYB1|ACKA_THEMA Acetate kinase OS=Thermotoga maritima (strain ATCC 43589 / MSB8 / DSM 3109 / JCM 10099) GN=ackA PE=1 SV=1

MRVLVINSGSSSIKYQLIEMEGEKVLCKGIAERIGIEGSRLVHRVGDEKHVIERELPDHE

EALKLILNTLVDEKLGVIKDLKEIDAVGHRVVHGGERFKESVLVDEEVLKAIEEVSPLAP

LHNPANLMGIKAAMKLLPGVPNVAVFDTAFHQTIPQKAYLYAIPYEYYEKYKIRRYGFHG

TSHRYVSKRAAEILGKKLEELKIITCHIGNGASVAAVKYGKCVDTSMGFTPLEGLVMGTR

SGDLDPAIPFFIMEKEGISPQEMYDILNKKSGVYGLSKGFSSDMRDIEEAALKGDEWCKL

VLEIYDYRIAKYIGAYAAAMNGVDAIVFTAGVGENSPITREDVCSYLEFLGVKLDKQKNE

ETIRGKEGIISTPDSRVKVLVVPTNEELMIARDTKEIVEKIGR

>sp|A0QLU8|ACKA_MYCA1 Acetate kinase OS=Mycobacterium avium (strain 104) GN=ackA PE=1 SV=1

MDGSDGARRVLVINSGSSSLKFQLVDPESGVAASTGIVERIGEESSPVPDHDAALRRAFD

MLAGDGVDLNTAGLVAVGHRVVHGGNTFYRPTVLDDAVIARLHELSELAPLHNPPALLGI

EVARRLLPGIAHVAVFDTGFFHDLPPAAATYAIDRELADRWQIRRYGFHGTSHRYVSEQA

AAFLDRPLRGLKQIVLHLGNGCSASAIAGTRPLDTSMGLTPLEGLVMGTRSGDIDPSVVS

YLCHTAGMGVDDVESMLNHRSGVVGLSGVRDFRRLRELIESGDGAAQLAYSVFTHRLRKY

IGAYLAVLGHTDVISFTAGIGENDAAVRRDAVSGMEELGIVLDERRNLPGAKGARQISAD

DSPITVLVVPTNEELAIARDCVRVLGG

>sp|B2HPZ3|ACKA_MYCMM Acetate kinase OS=Mycobacterium marinum (strain ATCC BAA-535 / M) GN=ackA PE=1 SV=1

MSASRPNRVVLVLNSGSSSLKFQLVEPDSGMSRATGNIERIGEESSSVPDHDAALRRVFE

ILAEDDIDLQSCGLVAVGHRVVHGGKDFYEPTLLNDAVIGKLDELSPLAPLHNPPAVLCI

RVARALLPDVPHIAVFDTAFFHQLPPAAATYAIDRELADVWKIRRYGFHGTSHEYVSQQA

AEFLGKPIGDLNQIVLHLGNGASASAVAGGRPVETSMGLTPLEGLVMGTRSGDLDPGVIG

YLWRTAKLGVDEIESMLNHRSGMLGLAGERDFRRLRAMIDDGDPAAELAYDVFIHRLRKY

VGAYLAVLGHTDVVSFTAGIGEHDAAVRRDTLAGMAELGISLDERRNACPSGGARRISAD

DSPVTVLVIPTNEELAIARHCCSVLVAV

>sp|Q73T33|ACKA_MYCPA Acetate kinase OS=Mycobacterium paratuberculosis (strain ATCC BAA-968 / K-10) GN=ackA PE=1 SV=1

MDGSDGARRVLVINSGSSSLKFQLVDPEFGVAASTGIVERIGEESSPVPDHDAALRRAFD

MLAGDGVDLNTAGLVAVGHRVVHGGNTFYRPTVLDDAVIARLHELSELAPLHNPPALQGI

EVARRLLPDIAHVAVFDTGFFHDLPPAAATYAIDRELADRWQIRRYGFHGTSHRYVSEQA

AAFLDRPLRGLKQIVLHLGNGCSASAIAGTRPLDTSMGLTPLEGLVMGTRSGDIDPSIVS

YLCHTAGMGVDDVESMLNHRSGVVGLSGVRDFRRLRELIESGDGAAQLAYSVFTHRLRKY

IGAYLAVLGHTDVISFTAGIGENDAAVRRDAVSGMEELGIVLDERRNLAGGKGARQISAD

DSPITVLVVPTNEELAIARDCVRVLGG

>sp|P75245|ACKA_MYCPN Acetate kinase OS=Mycoplasma pneumoniae (strain ATCC 29342 / M129) GN=ackA PE=1 SV=1

MNDNKILVVNAGSSSIKFQLFDYHKKVLAKALCERIFVDGFFKLEFNEQKVEEKVAFPDH

HAAVTHFLNTLKKHKIIQELSDIILVGHRVVQGANYFKDSVIVDAEALAKIKEFIKLAPL

HNKPEADVIEIFFKEVPSAKNVAVFDTTFHTTIPQENYLYAVPRSWEQKHLVRRYGFHGT

SYKFINNYLEKHLNKQNLNLIVCHLGNGASVCAIKNGKSFNTSMGFTPLEGLIMGTRSGD

LDPAIIGYVAEQENMSASDVVNALNKKSGMLALTGASDMRDVFAKPQENAVAIKMYVNRV

ADYIAKYLNQLEGNIDGLVFTGGIGENASDCVELFINAVKSLGFATDLKLFVKYGDSCVV

STPQSKYKIYRVRTNEELMIVEDSIRLTQK

>sp|P9WQH1|ACKA_MYCTU Acetate kinase OS=Mycobacterium tuberculosis (strain ATCC 25618 / H37Rv) GN=ackA PE=1 SV=1

MSSTVLVINSGSSSLKFQLVEPVAGMSRAAGIVERIGERSSPVADHAQALHRAFKMLAED

GIDLQTCGLVAVGHRVVHGGTEFHQPTLLDDTVIGKLEELSALAPLHNPPAVLGIKVARR

LLANVAHVAVFDTAFFHDLPPAAATYAIDRDVADRWHIRRYGFHGTSHQYVSERAAAFLG

RPLDGLNQIVLHLGNGASASAIARGRPVETSMGLTPLEGLVMGTRSGDLDPGVISYLWRT

ARMGVEDIESMLNHRSGMLGLAGERDFRRLRLVIETGDRSAQLAYEVFIHRLRKYLGAYL

AVLGHTDVVSFTAGIGENDAAVRRDALAGLQGLGIALDQDRNLGPGHGARRISSDDSPIA

VLVVPTNEELAIARDCLRVLGGRRA

>sp|Q99TF2|ACKA_STAAN Acetate kinase OS=Staphylococcus aureus (strain N315) GN=ackA PE=1 SV=1

MSKLILAINAGSSSLKFQLIRMPEEELVTKGLIERIGLKDSIFTIEVNGEKVKTVQDIKD

HVEAVDIMLDAFKAHNIINDINDIDGTGHRVVHGGEKFPESVAITDEVEKEIEELSELAP

LHNPANLMGIRAFRKLLPNIPHVAIFDTAFHQTMPEKAYLYSLPYHYYKDYGIRKYGFHG

TSHKFVSQRAAEMLDKPIEDLRIISCHIGNGASIAAIDGGKSIDTSMGFTPLAGVTMGTR

SGNIDPALIPFIMEKTGKTAEQVLEILNKESGLLGLSGTSSDLRDLSEEAESGKARSQMA

LDVFASKIHKYIGSYAARMHGVDVIVFTAGIGENSVEIRAKVLEGLEFMGVYWDPKKNEN

LLRGKEGFINYPHSPVKVVVIPTDEESMIARDVMTFGGLK
