# Supplementary material for: Seasonal and algal diet-driven patterns of the digestive microbiota of the European abalone Haliotis tuberculata, a generalist marine herbivore
Source: Microbiome. 2018 Mar 27;6:60. doi: 10.1186/s40168-018-0430-7 (PMC5870069; doi:10.1186/s40168-018-0430-7)
Supplement: Supplementary file 8 — Supplementary text. Supplementary information on methods used and results analysed for the study. (DOCX 43 kb) [file 40168_2018_430_MOESM8_ESM.docx]

**Seasonal and algal diet-driven patterns of the digestive microbiota of a generalist marine herbivore**

Angélique Gobet^1*^, Laëtitia Mest^1^, Morgan Perennou^2^, Simon Dittami^1^, Claire Caralp^3^, Céline Coulombet^4^, Sylvain Huchette^4^, Sabine Roussel^3^, Gurvan Michel^1*^ and Catherine Leblanc^1*^

**Additional file 8**

**Results**

***Pyruvate fermentation to acetate MetaCyc pathways***

In *Bacteria*, pyruvate fermentation to acetate may follow different pathways: MetaCyc pathways I, II and IV, which differ in the enzyme acting in the first reaction. In pathway I, pyruvate is transformed by a pyruvate synthase to an acetyl-CoA and CO_2_ [1]. In pathway II, the multienzyme pyruvate dehydrogenase complex, composed of the dihydrolipoyllysine-residue acetyltransferase (*Mp-pdhC*), the pyruvate dehydrogenase acetyl-transferring (*Mp-pdhAB*) and the dihydrolipoyl dehydrogenase (*Mp-pdhD*), transforms pyruvate to acetyl-CoA and CO_2_ [2]. Finally, in pathway IV, a pyruvate formate-lyase catalyzes pyruvate to acetyl-CoA and formate [3]. Common to all 3 pathways is the catalysis of acetyl-CoA to acetyl phosphate by a phosphate acetyltransferase and then to acetate by an acetate kinase [4]. The 3 selected genomes contain putative enzymes allowing at least one of the 3 pathways: *P. atlanticus* DSM 19335, pathways I, II and IV; *V. halioticoli*, pathways II and IV and *M. mobile* 163K, pathway II (Table S4, Fig. 6).

**Materials and Methods**

***Abalone breeding and measurements of abalone characteristics and algal composition***

The current study was part of a larger experiment which tested the effect of a monoalgal diet on abalone growth and sexual maturation (S. Roussel, personal communication). Juveniles were bred for about a year on land in nursery tanks at the abalone farm France Haliotis and then transferred to sea-based experimental cages for about 6 months. In January 2012, 1,000 abalone were randomly put in each cage of Abblox structures (structure of four cages commonly used in abalone aquaculture, Fig. S6C). A total of nine Abblox structures were placed at 5-m depth on the seafloor so that they were constantly immersed and cages were supplied with one of nine algal diets (a mixed algal diet depending on seasonal availability, *Enteromorpha sp*, *Ulva lactuca*, *Palmaria palmata*, red filamentous algae such as *Asparagopsis armata*, stipes of *Laminaria hyperborea*, *Saccorhiza polyschides*, *Laminaria digitata*, or *Saccharina latissima*). To search for relationships between the abalone digestive microbiota and the algal diet, we selected abalone fed with four of these nine algal diets: *P. palmata*, *L. digitata*; *S. latissima*, *U. lactuca*.

From February 2012 to January 2013 every 2-3 months (in February, April, June, August and October 2012, and in January 2013), 30 individuals were taken from each cage for growth measurements and 12 individuals were taken in June 2012 for sexual maturation measurements. After weighing abalone and measuring shell length, abalone growth was determined by calculating several nutritional indices (Final weight-to-shell length ratio = W_tf_/L_tf_ (g.mm^-1^); Final daily growth rate in weight = (W_tf_-W_t0_)/day (g.day^-1^); Final daily growth rate in shell length = (L_tf_-L_t0_)/day (mm.day^-1^); with W_t0_: mean initial wet weight, W_tf_: mean final wet weight, L_t0_: mean initial length, L_tf_: mean final length, and day: number of days between the beginning and the end of the experiment). Abalone sexual maturation was determined by measuring relative gonad development, based on wet weight (gonado-digestive gland (GDG) ratio = GDG weight / total weight; Muscle ratio = muscle weight / total weight; Shell ratio = shell weight / total weight) and estimated gonad ratio (estimated gonad weight / total weight) was calculated based on the total area of the gonad estimated from sections of the GDG measured using the microscopy software ImageJ 1.45s (Wayne Rasband National Institutes of Health, USA) with estimated gonad weight = gonadal index * GDG weight and with gonadal index = number of pixels for the gonadal area / total number of pixels of the section [5]. Abalone mortality was measured by collecting empty shells before each feeding and it was calculated as the proportion of empty shells to the initial number of abalone put in the cage.

On each sampling date, before feeding the abalone, samples of 100 g of fresh algae were taken and frozen at -20°C for biochemical analyses. Dry matter content of each algal diet was determined by weighing before and after freeze-drying the samples. Soluble carbohydrate content was determined following the phenol-sulphuric acid colorimetric method [6]. Triplicates of dry algal powder were pooled in equal quantity for samples from February 2012, June 2012 and October 2012 and the Lowry method was applied to determine protein content [7]. Free and total amino acids (free and total: histidine, methionine, arginine, valine, leucine, isoleucine, lysine, threonine, phenylalanine, tryptophan) were analyzed using the AccQ-Tag Ultra derivatization kit (Waters, Milford, MA, USA). Derivatized amino acids were analysed using an Acquity UPLC-DAD system (Waters). Essential free and total amino acids for abalone, as described by [8], were then selected to further calculate correlations with the digestive microbiota data set.

***DNA extraction, metabarcoding and sequence processing***

*DNA extraction of the lyophilized gonado-digestive glands*

Ground lyophilized glands were put in MK28R tubes with an extraction buffer (composed of 2 M NaCl, 100 mM Tris-HCl pH 8, 2% CTAB, 50 mM EDTA pH 8, 2% polyvinylpyrrolidone (PVPP)) and ground using a Precellys 24 homogenizer at 6,800 rpm 3 times for 30 s (Bertin Technologies, France). The obtained mix was centrifuged at 11,000 g for 10 min and the retrieved supernatant was gently mixed to a final concentration of 1 mg.mL^-1^ of lysozyme. After 20 min at RT, the protocol of the NucleoSpin Plant II Midi kit (« 5.3 Genomic DNA from soil, compost, dung, and animal excrements », Macherey-Nagel GmbH & Co. KG, Germany) was followed from step 3 on. The extracted DNA was kept at -20°C until further processing.

*Library preparation for MiSeq sequencing of the abalone microbiota*

Library preparation for MiSeq paired-end sequencing was carried out following the 16S Metagenomic Sequencing Library Preparation protocol from Illumina (http://support.illumina.com/downloads/16s_metagenomic_sequencing_library_preparation.html, San Diego, CA, USA). We chose to amplify the V3-V4 variable region of the 16S rRNA gene (426 bp long) to target *Bacteria* (forward primer, S-D-Bact-0341-b-S-17: CCTACGGGNGGC-WGCAG, reverse primer, S-D-Bact-0785-a-A-21: GACTACHVGGGTATCTAATCC, following [9]). Each primer was attached to an Illumina adapter overhang nucleotide sequence following this scheme: adapter-primer. The reaction of 25 µL included 5 ng.µL^-1^ genomic DNA mixed with 1 µM of each primer and 12.5 µL of 2x KAPA HiFi HotStart ReadyMix. PCR amplification was performed using the following program: 95°C for 3 min, 25 cycles of 95°C for 30 s, 55°C for 30 s, 72°C for 30 s and a final elongation step of 5 min at 72°C. The length of the amplified region was then verified on a Bioanalyzer 2100 (Agilent, Santa Clara, USA) and purified from any remaining free primers or free dimer species using AMPure XP beads (Beckman-Coulter, Brea, USA). Amplicons from abalone digestive gland triplicates were then pooled together and attached to dual indices and Illumina sequencing adapters with the Nextera XT Index Kit. A reaction of 50 µL was prepared and included 5 µL of amplified DNA, 5µL of the Nextera XT Index Primers 1 and 2, 25 µL of 2x KAPA HiFi HotStart ReadyMix and 10 µL of PCR grade water. PCR amplification was performed using the following program: 95°C for 3 min, 8 cycles of 95°C for 30 s, 55°C for 30 s, 72°C for 30 s and a final elongation step of 5 min at 72°C. All but one sample (from the *Ulva lactuca* diet in cage 1 taken on January 2013) successfully amplified the V3-V4 region of the 16S rRNA gene. The resulting amplicons were then purified with AMPure XP beads. Length and quantification of the amplicons were then carried out on a Bioanalyzer 2100 and an equimolar pool of all samples was prepared and further mixed with 5% PhiX as internal standard. Sequencing on a MiSeq using a MiSeq Reagent kit v3 (2 x 300 cycles) resulted in about 10 million sequences.

*Sequence processing*

The quality of the sequences was checked from the fastq files by using the fastx_toolkit from the Hannon lab (http://hannonlab.cshl.edu/fastx_toolkit/). The fastq_quality_trimmer allowed trimming nucleotides from the end of the sequence with a quality lower than 25 and discarding sequences shorter than 200 bp, and the fastq_quality_filter allowed removing sequences with a quality score lower than 20 over 75 bp. Sequences were then checked for their corresponding pair in the corresponding sample using an in-house Python script (get_paired.py, http://abims.sb-roscoff.fr/galaxyproject). The obtained sequences were curated using mothur v.1.34.4 and following the mothur MiSeq SOP [10]. After forming the contigs, primers, ambiguous bases, and homopolymers of more than 8 bases were discarded. Sequences were then aligned against Silva release 119 and further denoised by applying a pre-clustering allowing for up to 4 differences between sequences. Chimeras were then removed with UCHIME [11]. Sequences were taxonomically classified with the RDP classifier [12] on the Silva release 119 [13] and unexpected lineages using the selected primers (*Archaea*, Eukarya) were removed. OTUs were clustered at 97% sequence identity using the average neighbor algorithm [10].

***Diversity analyses and ecological patterns of the digestive microbiota***

OTUs present in the negative control and the sample *Ulva lactuca* diet, cage U1, January 2013, from which the amplification did not succeed, were removed from the whole data set. Additionally, OTUs appearing only once in the dataset were removed before analyses. This resulted in 5.3 million sequences and 3,945 OTUs at 97% sequence identity.

After calculating the Bray-Curtis dissimilarity index, the analysis of similarity (ANOSIM) allowed testing the grouping of samples according to sampling date, season or algal diet. An R value above 0.75 indicates different groups of samples, between 0.25 and 0.75; groups of samples are different but with some overlap and below 0.25; there are almost no differences between groups of samples.

To investigate taxa-environment relationships, we worked on contextual parameters measured in a parallel study (Roussel, personal communication) and the best combinations of these to explain the variation in the microbial community matrix were determined by forward selection (based on a canonical redundancy analysis algorithm and 999 Monte Carlo permutation tests). Abalone characteristics included final daily growth rate in length (mm.day^-1^), ratio between abalone weight and length, and estimated gonad weight on total weight ratio (%). Algal composition included total exocarbohydrate content (% dry matter), % dry matter content, and total amino acid content (here: methionine, arginine, valine, leucine). Sampling dates were represented as binary data (1 indicating the date when a sample was collected, 0 otherwise) for April 2012, June 2012, October 2012 and January 2013. Water temperature was not included in the model as it was highly collinear to the three other arguments.

***Gene annotation of the three dominant core genera***

Pyruvate to acetate fermentation was investigated in the selected genomes using a manually created database including sequences of the enzymes involved in the according pathways. The database was created following the steps: (*i*) searching for information on pyruvate fermentation to acetate pathways in MetaCyc (https://metacyc.org/META/NEW-IMAGE?object=Super-Pathways&detail-level=3) and obtaining the summary of the reaction and the EC number of the enzymes, (*ii*) checking for enzyme information using the EC numbers on the EBI website at http://www.ebi.ac.uk/thornton-srv/databases/enzymes/; (*iii*) for each EC number, going to the ExPASy database and selecting “All UniProtKB/Swiss-Prot entries referenced in this entry, with possibility to download in different formats, align etc.”; (*iv*) going to UniprotKB to get experimentally identified enzyme sequences by selecting “protein existence [PE] and “Evidence at protein level” in the advanced filter at the top of the page; (*v*) downloading and saving the sequences in a fasta file (Table S6).

**References**

1. Meinecke B, Bertram J & Gottschalk G. Purification and characterization of the pyruvate-ferredoxin oxidoreductase from *Clostridium acetobutylicum*. Arch. Microbiol. 1989; 152: 244–250

2. Himmelreich R et al. Complete sequence analysis of the genome of the bacterium *Mycoplasma pneumoniae*. Nucleic Acids Res. 1996; 24: 4420–49

3. Heßlinger C, Fairhurst SA & Sawers G. Novel keto acid formate-lyase and propionate kinase enzymes are components of an anaerobic pathway in *Escherichia coli* that degrades L-threonine to propionate. Mol. Microbiol. 1998; 27: 477–492

4. Boynton ZL, Bennett GN & Rudolph FB. Cloning, sequencing, and expression of genes encoding phosphotransacetylase and acetate kinase from *Clostridium acetobutylicum* ATCC. 1996; 62: 2758–2766

5. Shepherd SA & Laws HM. Studies on Southern Australian Abalone (Genus *Haliotis*) II .* Reproduction of Five Species. 1974; 49–62

6. Dubois M, Gilles KA, Hamilton JK, Rebers PA & Smith F. Colorimetric Method for Determination of Sugars and Related Substances. Anal. Chem. 1956; 350–356

7. Lowry OH, Rosebrough NJ, Lewis Farr A & Randall RJ. Protein measurement with the folin Phenol reagent. J. Biol. Chem. 1951; 265–275

8. King RH, Rayner CJ, Kerr M, Gorfine HK & McShane PE. The composition and amino acid balance of abalone (*Haliotis rubra*) tissue. Aquaculture. 1996; 140: 109–113

9. Klindworth A et al. Evaluation of general 16S ribosomal RNA gene PCR primers for classical and next-generation sequencing-based diversity studies. Nucleic Acids Res. 2013; 41: e1

10. Kozich JJ, Westcott SL, Baxter NT, Highlander SK & Schloss PD. Development of a dual-index sequencing strategy and curation pipeline for analyzing amplicon sequence data on the MiSeq Illumina sequencing platform. Appl. Environ. Microbiol. 2013; 79: 5112–20

11. Edgar RC. UPARSE: highly accurate OTU sequences from microbial amplicon reads. Nat. Methods. 2013; 10: 996–8

12. Wang Q, Garrity GM, Tiedje JM & Cole JR. Naïve Bayesian classifier for rapid assignment of rRNA sequences into the new bacterial taxonomy. Appl. Environ. Microbiol. 2007; 73: 5261–5267

13. Quast C et al. The SILVA ribosomal RNA gene database project: Improved data processing and web-based tools. Nucleic Acids Res. 2013; 41: 590–596

14. Gower JC. Some Distance Properties of Latent Root and Vector Methods Used in Multivariate Analysis. Biometrika. 1966; 53: 325–338

15. Peres-Neto PR & Jackson DA. How well do multivariate data sets match? The advantages of a Procrustean superimposition approach over the Mantel test. Oecologia. 2001; 129: 169–178
